# Characterization of the Maize Chitinase Genes and Their Effect on *Aspergillus flavus* and Aflatoxin Accumulation Resistance

**DOI:** 10.1371/journal.pone.0126185

**Published:** 2015-06-19

**Authors:** Leigh K. Hawkins, J. Erik Mylroie, Dafne A. Oliveira, J. Spencer Smith, Seval Ozkan, Gary L. Windham, W. Paul Williams, Marilyn L. Warburton

**Affiliations:** 1 USDA-ARS Corn Host Plant Resistance Research Unit, Mississippi State, Starkville, MS, United States of America; 2 Department of Biochemistry, Molecular Biology, Entomology, and Plant Pathology, Mississippi State University, Starkville, MS, 39762, United States of America; Utah State University, UNITED STATES

## Abstract

Maize (*Zea mays* L.) is a crop of global importance, but prone to contamination by aflatoxins produced by fungi in the genus *Aspergillus*. The development of resistant germplasm and the identification of genes contributing to resistance would aid in the reduction of the problem with a minimal need for intervention by farmers. Chitinolytic enzymes respond to attack by potential pathogens and have been demonstrated to increase insect and fungal resistance in plants. Here, all chitinase genes in the maize genome were characterized via sequence diversity and expression patterns. Recent evolution within this gene family was noted. Markers from within each gene were developed and used to map the phenotypic effect on resistance of each gene in up to four QTL mapping populations and one association panel. Seven chitinase genes were identified that had alleles associated with increased resistance to aflatoxin accumulation and *A*. *flavus* infection in field grown maize. The chitinase in bin 1.05 identified a new and highly significant QTL, while chitinase genes in bins 2.04 and 5.03 fell directly beneath the peaks of previously published QTL. The expression patterns of these genes corroborate possible grain resistance mechanisms. Markers from within the gene sequences or very closely linked to them are presented to aid in the use of marker assisted selection to improve this trait.

## Introduction

Maize (*Zea mays* L.), a major industrial commodity, feed grain, and staple food for many people in the developing world, is especially prone to contamination by aflatoxins produced by the fungi in the genus *Aspergillus* [1]. Because of the established ability of aflatoxins to suppress human and animal immune systems, negatively impact growth and development, and induce liver cancer, aflatoxins are the most widely studied mycotoxins. Over 100 countries have established or proposed regulations for controlling aflatoxins in foods and feed [2–4]. The U.S. Food and Drug Administration [5] has set a tolerance level of 20 ng·g^-1^ for aflatoxin B_1_ for maize designated for human consumption; currently, no commercially available US maize hybrids show sufficient levels of resistance to aflatoxin accumulation when faced with high disease pressure.

A desirable approach for reducing *A*. *flavus* infection and/or aflatoxin production is the development of resistant germplasm. Several natural sources of resistance have been identified in maize, but transfer of resistance into elite breeding lines is hampered by the quantitative nature of the trait and high genotype by environment interactions [6]. The identification of specific genes with a significant effect on resistance to *A*. *flavus* or aflatoxin accumulation would enable more rapid production of resistant maize inbred lines and hybrids. Genes encoding pathogenesis-related (PR) proteins include several evolutionarily conserved families with individual family members differing widely in occurrence and activity [7]. Chitinolytic enzymes belong to four recognized families of PR proteins (PR-3, PR-4, PR-8, and PR-11) that respond to attack by potential pathogens or the presence of elicitor treatments and a variety of abiotic stresses [7–13].

Chitinolytic enzymes are found in microorganisms, plants, and animals, and hydrolyze chitin, a major component of fungal cell walls, to *N*-acetylglucosamine (GlcNAc) [9, 14]. The GlcNAc subunits serve as signal molecules in phytopathogenic responses and are also part of the sugar chain of glycoproteins and glycolipids in plants [10]. Several observations suggest that the primary function of the induced expression of plant chitinases, acting alone or in combination with β-1,3-glucanases, is defense against fungal pathogens [9, 13, 15]. Constitutively-expressed chitinases function in a range of physiological and morphological processes including embryogenesis, flowering, senescence, and seed germination [7, 11, 13, 16, 17]. Based on physiochemical and enzymatic properties, chitinases belong to the Glycoside Hydrolase (GH) superfamily (IPR017853) [13, 18]. The endochitinases (E.C. 3.2.1.14) cleave the chitin chain randomly and are found in GH families 18 and 19. Chitinases in GH family 18 are ubiquitous while those in GH family 19 are found in plants and *Streptomyces* [10, 13]. Exochitinases (*β*-hexosaminidase (EC 3.2.1.52)) cleave GlcNAc subunits from the non-reducing end of the chitin molecule, and are found in GH family 20 [10, 13, 18]. Previous proteomic studies of *A*. *flavus* and aflatoxin accumulation in maize have identified possible links between some chitinase sequences and resistance. Studies using susceptible and resistant maize inbred lines have identified several differentially regulated proteins, including the named chitinase genes PRm3 (S82314), chitinase I (Q6JBN0 and B6TFQ3) and chitinase A (Q6JBK8 and P29022) [19], and an unidentified endochitinase [20].

The effect of any gene sequence on the phenotype of an organism can be measured via linkage and association mapping. When both of these techniques are performed on the same sequence and phenotype, they greatly complement each other. Association mapping identifies DNA polymorphisms linked to the causal mutation of a phenotype and can provide resolution to within hundreds or a few thousand base pairs [21, 22]. Due to the diversity that can be screened in an association study, the most favorable alleles of a gene series can be identified simultaneously. Genetic or quantitative trait loci (QTL) mapping does not lead to such high levels of resolution, but can more precisely measure the effects of a genomic region because mapping populations usually represent a less genetically complex background, and alleles are present in balanced proportions, leading to stronger statistical power. While genome-wide association studies (GWAS) simultaneously measure association between trait(s) of interest and thousands of sequence polymorphisms, candidate gene based association mapping is a very powerful hypothesis-driven method for testing a few sequences at a time, and it does not suffer from multiple testing problems that plague GWAS analyses.

The Corn Host Plant Resistance Research Unit (CHPRRU) of the USDA ARS has developed resources for the association mapping and QTL mapping of aflatoxin accumulation and *A*. *flavus* resistance in maize [6, 23]. These include four QTL mapping populations created with diverse parents, ensuring the possibility of finding polymorphisms between the parents for many candidate gene sequences [24–27] and an association mapping panel of diverse maize lines [23]. All genetic resources have been phenotyped for aflatoxin accumulation and related traits in replicated, multi-year and location field trials. In light of the evidence that some maize chitinase genes may actively contribute to *A*. *flavus* resistance, the objectives of this study were to characterize all major chitinase genes in maize; test them for their ability to increase resistance to aflatoxin accumulation and *A*. *flavus* infection in field grown maize; and identify and design easy-to-use markers for the alleles that increase resistance for the marker-assisted improvement of this trait.

## Materials and Methods

The databases from MaizeGDB [28], Gramene (B73 Reference Sequence) [29], UniProt [30], InterPro [31], UniGene [32, 33] and Carbohydrate-Active enZYmes (CAZY) [34] were queried for any gene and/or protein with maize chitinolytic activity or sequence homology. The MaizeCyc database [35] was also searched for any gene member with chitinase activity (GO:0004568), chitin catabolic activity (GO:0006032), and/or chitin binding (GO:0008061) (S1 Table). In addition, a literature search was conducted to find published maize chitinase genes. A total of thirty-three unique genes were identified: 13 GH-18 family members; 17 GH-19 family members and 3 GH-20 family members (Table 1). These genes were found on every maize chromosome except chromosome 9. An attempt was made to avoid redundancy in this table, as there were often different Uniprot or Unigene designations for different transcripts and/or proteins of the same gene (S1 Table). Sequences physically very close together were aligned to ensure they were not the same gene being reported by different authors or databases. A table of all final gene and protein sequences used in this study can be found in S2 Table.

**Table 1:** List of maize chitinases (in order by chromosomal position).

#	Glycoside Hydrolase Family	Gramene	UniProt	Description	Bin	Position				References
						Chr	From	To	+/-	
1	GH-19	GRMZM2G099454	B6T6W1	Basic endochitinase C	1.01	1	7,403,531	7,408,184	+	10, 29, 30, 35, 38
2	GH-19	GRMZM2G312226		Chitinase family protein	1.02	1	22,893,595	22,594,905	-	30, 35
3	GH-20	GRMZM2G134251	B4F7Z2	Beta-hexosaminidase	1.02	1	27,303,824	27,306,170	-	10, 28, 30, 38
4	GH-19	GRMZM2G103668	B6SZN3	Putative uncharacterized protein	1.05	1	85,545,979	85,547,146	+	10, 29, 30, 35, 38
		GRMZM2G544531				1	85,544,822	85,546,118	-	
5	GH-18	GRMZM2G162505	B4G1L5	Chitinase 2	1.08	1	240,766,113	240,767,264	+	10, 29, 30, 35, 38
		GRMZM2G057093				1	240,756,899	240,757,825	+	29, 35
6	GH-19	GRMZM2G051921	C0PKN5	Putative uncharacterized protein	2.04	2	33,506,460	33,508,810	+	29, 30, 35, 38
7	GH-19	GRMZM2G051943	P29022	Endochitinase A	2.04	2	33,534,101	33,535,449	+	28, 30, 35, 38
8	GH-19	GRMZM2G052175	B6SZA3	Endochitinase A	2.04	2	33,537,139	33,539,360	+	29, 30, 35, 38
		GRMZM2G122708		Homolog of carrot EP3-3 Chitinase	2.04	2	33,558,850	33,559,772	-	30, 35, 38
9	GH-18	GRMZM2G403475	B6U1W5	Putative uncharacterized protein	3.04	3	118,806,302	118,807,527	+	30, 35
10	GH-18	GRMZM5G837822	B6TVA3	Hevamine-A	3.06	3	176,570,295	176,571,270	-	10, 29, 30, 35, 38
		GRMZM2G430936	none	Chitinase A	3.06	3	176,597,396	176,598,753	-	30, 35
		GRMZM2G430942	none	Chitinase A	3.06	3	176,599,800	176,601,118	-	30, 35, 38
		GRMZM2G023650	none	Chitinase A	3.06	3	176,676,857	176,678,050	-	30, 35, 38
11	GH-18	GRMZM2G453805	P93518	Prm3, chem5, chn1	3.08	3	212,009,199	212,001,553	-	10, 29, 30, 35, 38
12	GH-18	GRMZM2G133781	B4FR99	Putative uncharacterized protein	4.02	4	6,624,888	6,626,733	+	10, 35, 38
13	GH-18	GRMZM2G358153	B4FBN8	Chitinase 1	4.03	4	12,082,697	12,084,089	+	30, 35, 38
14	GH-19	GRMZM2G064360	B6TFQ3	Basic endochitinase 1	5.03	5	63,229,136	63,230,645	+	10, 30, 35, 38
15	GH-19	GRMZM2G389582	Q41795	Chitinase chn3	5.03	5	63,277,951	63,280,101	+	10, 29, 30, 35, 38
		GRMZM2G389557				5	63,277,675	63,280,116	-	
16	GH-19	GRMZM2G129189	B6TT00	Endochitinase PR4	5.05	5	182,518,442	182,519,605	+	10, 30, 35, 38
17	GH-18	GRMZM2G057766	B6SZG2	Chitinase 1	5.06	5	197,564,845	197,565,921	-	30, 35, 38
18	GH-18	GRMZM2G141456	B4F9H4	Chitinase	5.08	5	215,173,302	215,174,990	-	30, 35, 38
19	GH-20	GRMZM2G034598	B6ST04	Beta-hexosaminidase	6.01	6	34,442,195	34,456,096	+	10, 28, 38
20	GH-19	GRMZM2G412577	K7UKQ3	Uncharacterized protein	6.01	6	71,462,625	71,463,278	+	28, 29, 30, 38
21	GH-19	GRMZM2G145518	B8A247	Putative uncharacterized protein	6.01	6	82,813,531	82,815,309	+	10, 30, 35, 38
		GRMZM2G447967				6	82,813,748	82,814,906	-	
22	GH-19	GRMZM2G145461	B4G0Q3	Acidic class I chitinase	6.01	6	82,862,563	82,863,789	-	10, 30, 35, 38
23	GH-18	GRMZM2G447795	B6U2X8	Xylanase inhibitor protein 1	6.05	6	129,086,004	129,087,221	+	10, 35, 38
24	GH-18	GRMZM2G328171	B6SGT3	Xylanase inhibitor protein 1	7.01	7	10,575,103	10,576,227	-	38
		GRMZM2G162359	B4G1C2	Uncharacterized protein	7.01	7	10,651,271	10,653,006	+	10, 38
25	GH-19	GRMZM2G168364	B4G1H3	Endochitinase A2	7.03	7	134,135,706	134,137,488	+	10, 30, 35, 38
26	GH-19	GRMZM2G400497	K7VNG7	Uncharacterized protein	8.00	8	839,459	841,251	+	28,29,30, 38
27	GH-19	GRMZM2G062974	B6TR38	Basic Endochitinase A	8.03	8	88,812,713	88,814,117	+	10, 30, 35, 38
28	GH-19	GRMZM2G083292	KYVXP1	Putative uncharacterized protein	8.03	8	129,341,989	129,343,096	+	29, 30, 38
29	GH-18	GRMZM2G037694	B6TAN9	Hydrolase, hydrolyzing O-glycosyl compound	8.03	8	144,588,215	144,595,174		35
30	GH-20	GRMZM2G117405	C0PLT8	Beta-hexosaminidase	8.03	8	164,558,359	164,563,720	+	10, 27, 38
31	GH-18	GRMZM2G400999	B6U3V2	Xylanase inhibitor protein 1	10.00	10	1,980,938	1,982,242	+	35, 38
32	GH-18	GRMZM2G090441	C0PIX9	Uncharacterized protein	10.04	10	107,900,408	107,902,133	+	30, 38, 46
33	GH-19	GRMZM2G005633	P29023	Endochitinase B	10.04	10	127,370,239	127,371,829	-	10, 27, 30, 34, 38

Gramene genetic sequence identification and UniProt protein identification numbers were used as unique identifiers of each chitinase in the study. Bin location indicates genetic mapping location according to MaizeGDB, and Position indicates the physical interval in relation to the B73 maize reference genome (Maize B73 RefGen_V2). Position on the positive or negative DNA strand is also indicated.

Two of the genes with very long introns (GRMZM2G099454, intron length 1878 and GRMZM2G034598, intron length 8683), were sequenced directly to confirm the reported B73 reference sequence. Fragments were amplified to “walk” down the length of each gene across the intron and flanking exonic regions. For GRMZM2G099454, amplicons successfully amplified in B73 and three other genotypes for most of the fragments; these were sequenced and aligned (data not shown). Thus we believe that this intron is real and correctly reported in the B73 reference sequence. For GRMZM2034598, the amplicons for the flanking exonic regions did amplify and the sequences correctly aligned to the B73 reference sequence. However, most of the amplicons from within the intron or in the intron/exon borders did not amplify and could not be sequenced. Although this is not a conclusive test, we therefore suspect that the reported sequence is incorrect at this intron (data not shown). This will not invalidate mapping tests with this gene reported here.

**Fig 1:**
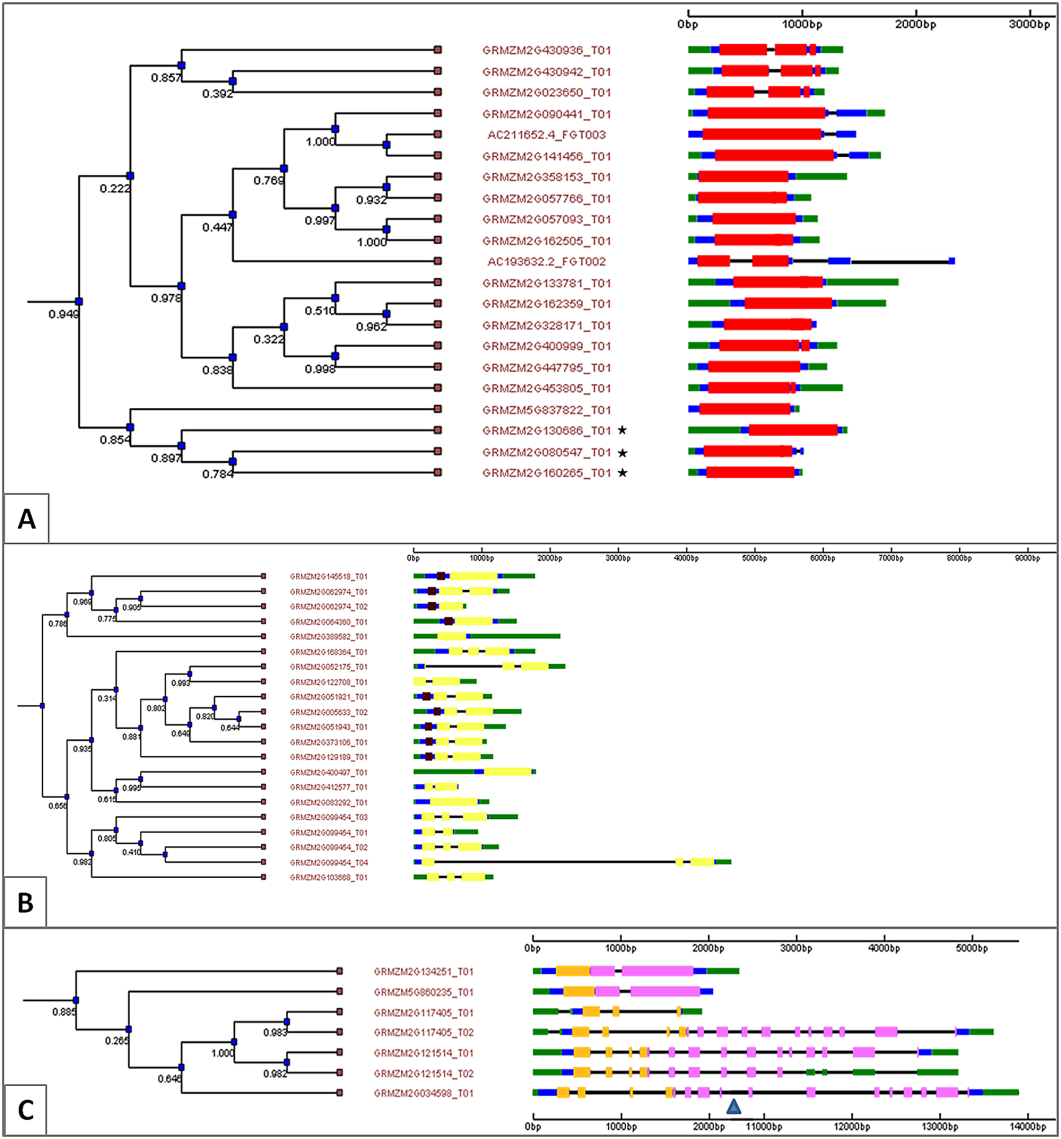
Dendrogram depicting relationships between gene sequences and gene transcripts within each glycoside hydrolase family (18, 19, and 20) and diagram of gene structure and protein motif for each chitinase using the PIECE database [38]. (A) GH-18 family chitinases; (B) GH-19 family chitinases; (C) GH-20 family chitinases, Intron (black); CDS (blue); untranslated region (UTR, Green); Glycoside Hydrolase protein family 18 domain (PF00704 (red)); FHIPEP protein family for type III secretions (PF00771 (purple)). Glycoside Hydrolase protein family 19 (PF00182 (yellow)); Carbohydrate-binding module protein family (PF00187 (brown)); Glycoside hydrolase protein family 20, domain 2 protein family (PF02838 (orange)), Glycoside Hydrolase protein family 20 PF00728 (pink). Blue triangle denotes large intron in GRMZM2G034598. Note change in scale.

Very similar sequences were explored further using NCBI BLAST tools [36] to provide clues to recent evolutionary history. By searching the protein family (Pfam) motif IDs [37] and viewing gene structure using the genomic sequence option in the PIECE database [38], the chitinases were grouped and exon sequence similarities were viewed by their GH family (Fig 1). The transcription level and tissue specificity of each gene was obtained from the B73 derived gene atlas available from MaizeGDB [39–41] (S1 Fig).

The QTL mapping populations used for verification of phenotypic effects of each candidate gene consisted of four F_2:3_ linkage mapping families. These mapping populations have been characterized and published previously. The mapping populations derived from initial crosses between the following pairs of parents: Mp313E (resistant to aflatoxin accumulation) and Va35 (susceptible) [27], Mp313E and B73 (susceptible) [24]; Mp715 (resistant) and T173 (susceptible) [6]; and Mp717 (resistant) and NC300 (susceptible) [25]. F_1_ plants were selfed to create F_2_ plants, individual seeds of which were grown and selfed to create F_2:3_ families. DNA from each F_2_ plant was used for genotyping using insertion/ deletion (InDel), single nucleotide polymorphism (SNP), simple sequence repeat (SSR) or restriction fragment length polymorphism (RFLP) markers, and linkage mapping. F_2:3_ families were grown in replicated field tests in multiple environments (see individual mapping references for details on the phenotyping of each population). Briefly, ten plants of each family were individually inoculated with a 3.4-ml suspension of 3x10^8^ conidia of *Aspergillus flavus* strain NRRL 3357 (ATCC #200026) using the side-needle technique 7 d after mid-silk [42, 43]. Bulked grain samples of each family from dried, shelled ears were ground and the VICAM AflaTest (VICAM, Watertown, MA) was used to determine aflatoxin concentration in 50g samples of ground grain from each plot, according to manufacturer’s instructions.

SNP or InDel markers were designed from the sequence of each chitinase candidate gene and tested for polymorphism between the parents of all mapping populations (Table 2). SNPs were converted from sequence data into KASP assays (LGC Genomics, Hertz, UK). When polymorphisms were found, the markers were run on the entire mapping population to test the effect of each gene on the phenotype. When polymorphisms could not be identified in any mapping population for a given chitinase gene sequence, SSR markers within 10,000 Kb were used to test the effect of the region. Flanking markers were used when possible; in some cases, a marker further than 10,000 Kb, or of unknown physical but close genetic location, was used as one of the flanking markers, and a second flanking marker within 10,000 Kb was always used in these cases. Some of the chitinase sequences were within 10,000 Kb of each other; in these cases, QTL mapping did not resolve which of the sequences (if either) affected the trait. Mapping was done using the JoinMap mapping software (version 4) [44]; linkage groups were constructed using the Maximum Likelihood (ML) mapping method. Composite Interval Mapping (CIM) was performed using QTL Cartographer version 2.5 [45] as described by [25]. To estimate the 0.05 significance threshold for QTL, 1000 permutations were performed with each data set and across all data sets [46]. Mapping was done for each environment, location in each year, and across both locations and years, where each population was phenotyped.

**Table 2:** List of SNPs, InDels, and SSRs used to map the phenotypic effect of each candidate gene in the linkage mapping populations.

			Marker	To test		Estimated start
#	Gene(s)	Marker	Type	Bin	Population tested	location of marker
**1**	GRMZM2G099454	umc1292	SSR	1.01	MpT	3,732,780
		umc1727	SSR	1.01	MpT	8,560,823
		umc1948	SSR	1.01	MpB	8,537,358
**2**	GRMZM2G312226	umc1166	SSR	1.02a	MpT	15,080,522
		bnlg1429	SSR	1.02a	MpT	15,444,049
**3**	GRMZM2G134251	S1_27303546	SNP	1.02b	MpB	27,303,546
		umc1070	SSR	1.02b	MpT	17,660,941
**4**	GRMZM2G103668	S1_85545046	SNP	1.05	MpB, MpT	85,545,046
	GRMZM2G544531	umc2025	SSR	1.05	MpB	91,415,861
**5**	GRMZM2G162505	S1_240766861	SNP	1.08	MpT	240,766,861
	GRMZM2G057093	S1_240766882	SNP	1.08	MpNC	240,766,882
		S1_241404296	SNP	1.08	MpVa	241,404,296
		csu164	SSR	1.08	MpVa	250,092,343
**6**	GRMZM2G051921	bnlg108	SSR	2.04	MpB	47,170,490
**7**	GRMZM2G051943	S2_33534181	SNP	2.04	MpB	33,534,181
**8**	GRMZM2G052175	umc1018	SSR	2.04	MpB	unknown
	GRMZM2G122708	chi1MPVa	InDel	2.04	MpT	33,534,101
		ChiAMpVa	InDel	2.04	MpVa	33,534,101
		S2_33534181	SNP	2.04	MpB	33,534,181
**9**	GRMZM2G403475	S3_117291815	SNP	3.04	MpT	117,291,815
		umc1527	SSR	3.04	MpB, MpT, MpVa	118,064,950
		umc1773	SSR	3.04	MpB, MpVa	119,647,741
**10**	GRMZM2G837822	bnlg1350	SSR	3.06	MpT	178,184,249
	GRMZM2G430936	umc2267	SSR	3.06	MpT	179,843,083
	GRMZM2G430942					
	GRMZM2G023650					
**11**	GRMZM2G453805	umc1320	SSR	3.08	MpT, MpVa	213,547,173
		umc1140	SSR	3.08	MpVa	209,639,614
**12**	GRMZM2G133781	umc1288	SSR	4.02	MpT	5,499,842
		umc1294	SSR	4.02	MpNC	6,602,430
		umc1757	SSR	4.02	MpNC, MpT	4,753,629
		php20725	SSR	4.02	MpVa	unknown
**13**	GRMZM2G358153	umc2082	SSR	4.03	MpB	12,044,070
		nc005	SSR	4.03	MpT	36,881,090
		bnlg1126	SSR	4.03	MpNC	11,310,311
**14**	GRMZM2G064360	S5_63229609	SNP	5.03	MpT	63,229,609
**15**	GRMZM2G389582	S5_63229636	SNP	5.03	MpB, MpT, MpVa	63,229,636
**15**	GRMZM2G389557	S5_63229609	SNP	5.03	MpT	63,229,609
**16**	GRMZM2G129189	umc1155	SSR	5.05	MpB, MpNC, MpT, MpVa	180,186,573
		umc1687	SSR	5.05	MpT, MpVa	180,186,573
**17**	GRMZM2G057766	chi1_C5	InDel	5.06	MpT	197,564,845
**18**	GRMZM2G141456	umc1153	SSR	5.08	MpT	205,552,861
		umc108	SSR	5.08	MpVa	204,605,587
		umc2136	SSR	5.08	MpNC	unknown
**19**	GRMZM2G034598	bnlg1165	SSR	6.01a	MpT	27,494,827
**20**	GRMZM2G412577					
**21**	GRMZM2G145518	S6_82813940	SNP	6.01b	MpT	82,813,940
**21**	GRMZM2G447967	csu183	SSR	6.01b	MpVa	89,127,346
**22**	GRMZM2G145461					
**23**	GRMZM2G447795	umc1250	SSR	6.05	MpVa	127,445,565
		umc2580	SSR	6.05	MpB, MpNC	123,776,890
**24**	GRMZM2G162359	S7_10547605	SNP	7.01	MpT	10,547,605
**24**	GRMZM2G162359	S7_10652252	SSR	7.01	MpB, MpT, MpVa	10,652,252
**25**	GRMZM2G168364	bnlg1070	SSR	7.03	MpT	132,596,480
		umc1001	SSR	7.03	MpT	147,539,143
		phi114	SSR	7.03	MpT	153,583,248
		asg49	SSR	7.03	MpVa	129,865,901
**26**	GRMZM2G400497					
**27**	GRMZM2G062974	S8_88812804	SNP	8.03a	MpNC, MpVa	88,812,804
		S8_88814131	SNP	8.03a	MpB	88,814,131
**28**	GRMZM2G083292	bnlg666	SSR	8.03b	MpT	133,561,516
		bnlg162	SSR	8.03b	MpB	133,561,516
**29**	GRMZM2G037694	umc8.03	SSR	8.03	MpNC	unknown
		umc1141	SSR	8.03c	MpB, MpT, MpVa	138,860,175
		umc2210	SSR	8.03c	MpB, MpVa	142,142,542
**30**	GRMZM2G117405	S8_164558329	SNP	8.03d	MpT	164,558,329
		S8_165558375	SNP	8.03d	MpT	165,558,375
		S8_164558387	SNP	8.03d	MpT	164,558,387
		umc2395	SSR	8.03d	MpB, MpT	164,963,371
		umc2606	SSR	8.03d	MpT	164,445,025
**31**	GRMZM2G400999	bnl3.04	SSR	10	MpVa	2,462,036
		umc1291	SSR	10	MpT	2,462,036
**32**	GRMZM2G090441	umc1246	SSR	10.04a	MpT	95,276,847
		umc64	SSR	10.04a	MpVa	88,334,564
		umc1589	SSR	10.04a	MpNC	102,366,287
**33**	GRMZM2G005633	umc1053	SSR	10.04b	MpT	114,288,173
		umc1506	SSR	10.04b	MpB	133,215,331

Bin location and population in which the marker was segregating (and thus possible to map) is indicated.

**Fig 2:**
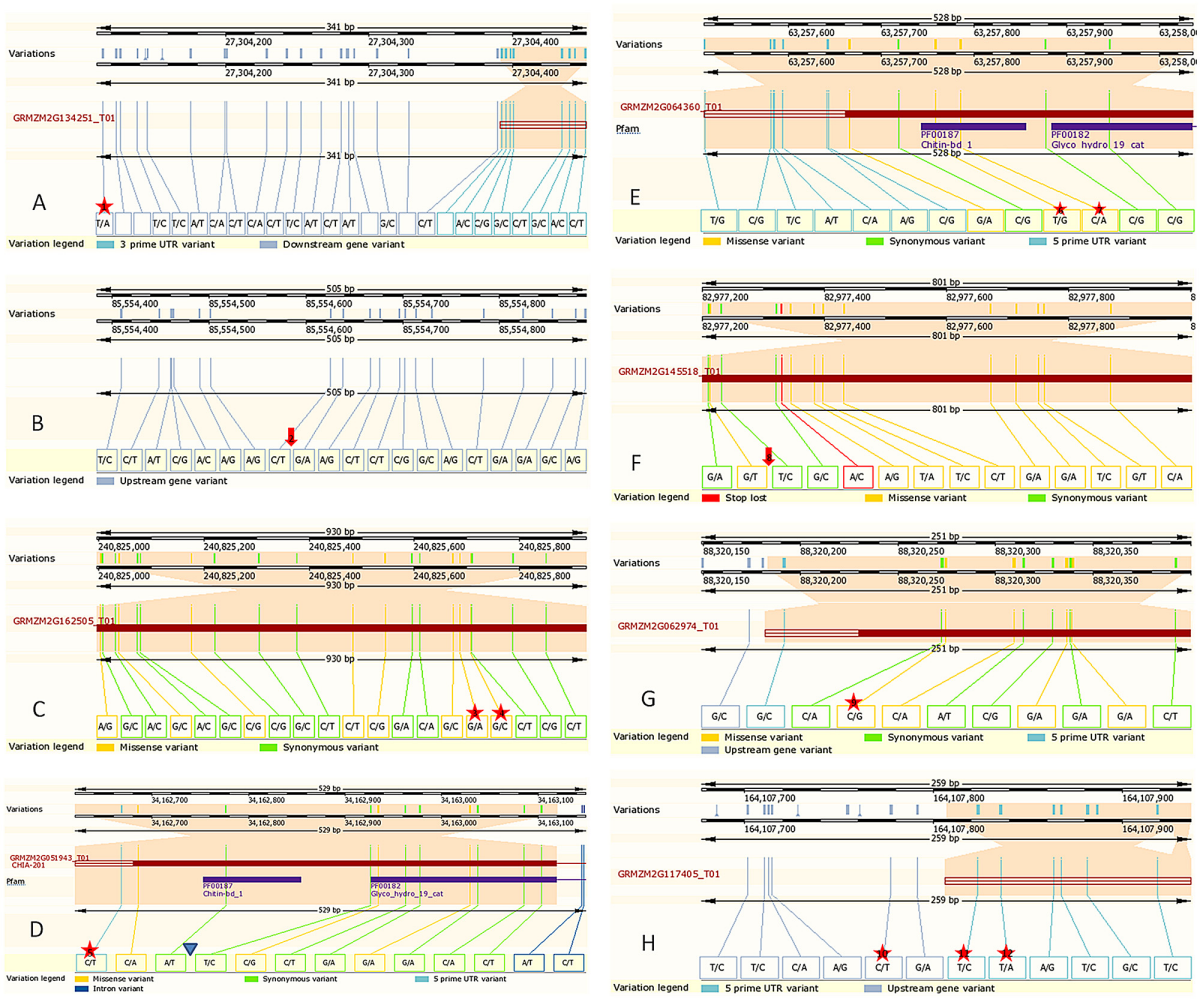
The position of single nucleotide and insertion/ deletion polymorphisms studied within the chitinase gene sequences or within 500 bp of the gene position (Adapted from gramene.org [29]). Schematics of each gene are drawn from information from the MaizeGDB [28]. Other genes in the study not shown here were mapped using further up-or down-stream markers. Footnotes: (A) GRMZM2G134251; (B) GRMZM2G103668; (C) GRMZM2G162505; (D) GRMZM2G051943; (E) GRMZM2G064360; (F) GRMZM2G145518; (G) GRMZM2G062974; (H) GRMZM2G117405. Orange section is the gene of interest. Red stars and/or arrows indicate position of SNP used. (1) S1_27303546, (2) S1_85545046, (3) S1_240766861, (4) S1_240766882, (5) S2_33534181, (6) S1_63229609, (7) S1_63229636, (8) S6_82813940, (9) S8_88812804, (10) S8_164558329; (11) S8_165558375; (12) S8_164558387. Blue Triangle indicates position of insertion/ deletion polymorphism ChiAMpVa. SNP positions provided as (Maize B73 RefGen_V2) adjusted to reflect correct position on image (Maize B73 RefGen_V3).

The aflatoxin association mapping panel consisted of 287 diverse inbred lines, which have been characterized as described previously [23]. Briefly, testcrosses were formed with Va35, a susceptible, southern adapted inbred line of the non-stiff stalk heterotic pattern, and grown in seven environments. Plants were inoculated and phenotyped as described in the QTL mapping populations, above. Proc GLIMMIX from the SAS statistical software package [47] was used to calculate LSMEANS of aflatoxin levels using a Generalized Linear Mixed Model (GLMM, [48]). Both log transformed and untransformed (but not quite normal) data were used in the association analysis. Genotyping of the 287 entries in the panel was done via Genotyping by Sequencing (GBS) according to [49]. A data subset consisting of 2000 SNPs was used to calculate population substructure using Structure 2.2 [50], and a kinship matrix using PowerMarker v. 3.25 [51] to correct for population substructure during association analysis using the mixed linear model (MLM) of TASSEL 3.0.1[52, 53]. SNPs within the reported genetic sequences of the chitinase candidate genes (Table 1), or when necessary, within a +/- 15 Kb window, were extracted from the GBS dataset for association analysis and are listed in S3 Table and Fig 2. SNPs were filtered to remove those with a minor allele frequency of less than 5% before association analysis.

## Results

### Identification and Characterization of Maize Chitinolytic Enzymes

The published literature and several online databases were searched to identify maize chitinase genes, and a total of 33 were identified. A brief description of each identified chitinase along with Gramene number, UniProt ID, and chromosomal position are found in Table 1. Additional information on gene ontology and other identifiers for each gene can be found in S1 Table. There were four instances where two genes appear in nearly identical chromosomal positions but lie on opposite strands of the DNA molecule: GRMZM2G103668 and GRMZM2G544531; GRMZM2G389582 and GRMZM2G359557; GRMZM2G145518 and GRMZM2G447967; and GRMZM2G052175 and GRMZM2G122708 (Table 1). There were also other discrepancies when examining information from different websites. For example, a chitinase polypeptide identified in the MaizeCYC database (AC211652.4_FG003) shares a Uniprot ID (B4F9H4) and a protein reference sequence (NP_00130521.1) with the chitinase gene GRMZM2G141456; however different chromosomal locations were reported for them (AC2116524_FGP003 at Chromosome 4:20,448,273–20,449,744 and GRMZM2G141456 at chromosome 5:215,173,407–215,174,878). The dendrogram in Fig 1 finds them nearly identical, and thus, one of the reported genetic locations may be in error.

Some other sequences appeared in Fig 1 but not in our study. Three gene identifiers (GRMZM2G130686, GRMZM2G160265, and GRMZM2G080547) that share the same gene structure in Fig 1 as the GH18 chitinases actually share no significant similarity with other chitinase genes at the level of the exon sequences. Another gene identified as a chitinase by the Plant Intron Exon Comparison and Evolution (PIECE) database (AC193632.2_FG002) has some similarity with the GH18 family chitinases, but the associated Uniprot ID K7TU58 appears to belong to the related protein kinase superfamily. GRMZM2G373106 is shown as a member of the GH-19 family; it may function differently as a lysozyme [28]. Though listed with the GH-20 family in Fig 1, GRMZM2G860235 does not appear in MaizeGDB database [28]. Also shown as a GH-20 family member is GRMZM2G121514, but different databases gave contradictory evidence regarding the function of this gene.

In some instances, several alleged chitinase genes with high percentages of homology were reported in the same region. On chromosome 3 from 176,597,396–176,678,080, 4 chitinase genes (GRMZM5G837822 (Hevamine A), GRMZM2G430936, GRMZM2G430942, and GRMZM2G023650) were reported by Monaco et al. [35]. Although they have slightly different gene structures (Fig 1), the exons were nearly identical, and thus only Hevamine A was included in this analysis. While there may actually be four tandemly arranged genes, if so, for the purposes of mapping they are all close enough together to include as one. In addition to the genes in Fig 1 that were not included in our study, there were six genes in our study that are not in Fig 1. This occurred because although the PIECE database did not identify them as chitinases, the Uniprot database did. These include GRMZM2G312226, GRMZM2G403475, GRMZM2389557, GRMZM2G447967, GRMZM2G145461, and GRMZM2G037694. The search for maize chitinases at Uniprot also returned entry Q4VQ82, but gene ontology indicates that it was an rRNA N-glycosidase, EC = 3.2.2.22 [30, 32, 33] and it is not included in the study.

### Mapping

Each candidate gene sequence was tested via QTL mapping in at least one and up to four mapping populations. Single nucleotide polymorphisms or InDel markers were identified within the sequence of 31 of the 33 chitinase genes (Fig 2; S3 Table). Where SNP or InDel polymorphisms were identified between the parents of a linkage population and within the sequence of the gene itself, these were tested directly; however, in the following chromosomal regions a paucity of polymorphisms were encountered, due to lack of diversity or difficulty in accurate sequencing: 4:12072697–12094089; 5:63252951–63305101; 5:182518442–215229990; 6:71,462,625–71,463,278; 6:82793531–82835309; 6:129061004–129115221; and 8:144588215–144595174. These regions may not be sufficiently surveyed to draw definitive conclusions from association analysis, and closely linked SSR markers were used instead for QTL mapping in these locations (Table 2).

**Fig 3:**
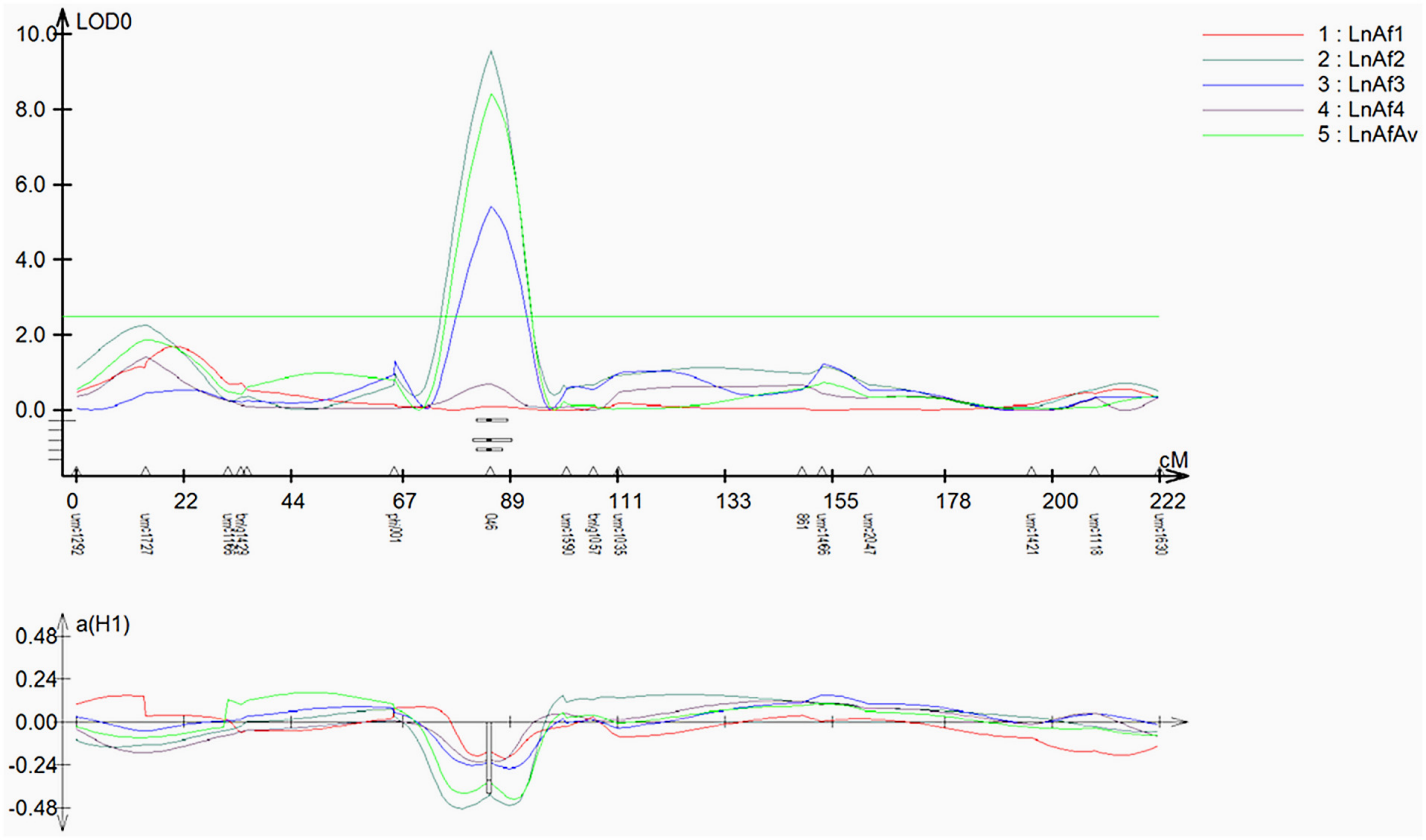
Linkage map from the F_2:3_ mapping population of Mp715 x T173 (n = 192) showing the position of a new QTL on chromosome 1 centered over the SNP within the sequence of GRMZM2G103668 in bin 1.05, a GH19 protein with chitinase activity. The mapping population was phenotyped and genotyped originally according to [25].

The results of the QTL mapping tests for each gene in each population in which they segregated are presented in Table 3, Fig 3, and S2 Fig. A SNP within the sequence of GRMZM2G103668 (in Bin 1.05) had the highest logarithm of the odds (LOD) score (9.2) in population Mp715 x T173. In this population, before this SNP was mapped, there was no significant peak at this location, and after the addition of this SNP, a tall and very well defined QTL appeared in two environments and in the average of the environments, directly over the SNP (Fig 3). The QTL was associated with additive gene action, and the percentage of phenotypic variation explained by the QTL (R^2^) was 17.8%, a large amount for a quantitative trait and comparable to many of the largest QTL identified for aflatoxin resistance to date [54]. The allele reducing aflatoxin levels came from the resistant parent (Mp715).

**Table 3:** Summary of QTL and candidate gene association mapping results.

#	Gramene	Description	Bin	# SNPs	# MLM	Allele effect		QTL pops.	LOD	Pheno.Effect	Gene action	Max R^2^
				tested	assos.	ave	max					
**1**	GRMZM2G099454	Basic Endochitinase C	1.01	10	2			MpB	ns	ns		
								MpT	ns	ns		
								MpVa	3.8	0.664	additive	0.131
**2**	GRMZM2G312226	Chitinase Family Protein	1.02	4	4	7.709	2.556	MpB	ns	ns		
					1*	7.709	2.556	MpT	ns	ns		
**3**	GRMZM2G134251	Beta-hexosaminidase beta chain	1.02	3	0			MpB	ns	ns		
								MpT	ns	ns		
**4**	GRMZM2G103668	Putative uncharacterized protein; CHIT14 precursor	1.05	4	1	-54.356	-358.606	MpB	ns	ns		
								MpT	9.2	-0.335	additive	0.178
**5**	GRMZM2G162505	Chitinase 2	1.08	27	18			MpT	ns	ns		
								MpNC	ns	ns		
								MpVa	2.6	-0.276	dominant	0.106
**6**	GRMZM2G051921	Putative uncharacterized protein; CHIT3 precursor	2.04	6	2	-9.459	-232.111	MpB	5.4	-0.313	additive	0.089
					1*	-9.459	-232.111	MpT	ns	ns		
								MpVa	4.8	-0.354	additive	0.241
**7**	GRMZM2G051943	Endochitinase A; SSR umc1783; CHIT3 precursor	2.04	7	10			MpB	5.4	-0.313	additive	0.089
								MpT	ns	ns		
								MpVa	4.8	-0.354	additive	0.241
**8**	GRMZM2G052175	Endochitinase A; CHIT2 precursor	2.04	7	10			MpB	5.4	-0.313	additive	0.089
								MpT	ns	ns		
								MpVa	4.8	-0.354	additive	0.241
**9**	GRMZM2G403475	Putative uncharacterized protein	3.04	4	1			MpB	4.8	-0.263	additive	0.067
								MpT	ns	ns		
								MpVa	ns	ns		
**10**	GRMZM5G837822	Hevamine-A	3.06	4	0			MpT	3.4	-0.198	additive	0.033
								MpVa	ns	ns		
**11**	GRMZM2G453805	Prm3, chem5, chn1	3.08	12	0			MpT	ns	ns		
								MpVa	ns	ns		
**12**	GRMZM2G133781	Putative uncharacterized protein	4.02	9	0			MpNC	ns	ns		
								MpT	ns	ns		
								MpVa	3.7	-0.190	additive	0.155
**13**	GRMZM2G358153	Chitinase 1; glycosyl hydrolase, putative	4.03	3	3	69.717	139.077	MpB	2.4	-0.184	additive	0.122
					1*	69.717	139.077	MpNC	ns	ns		
								MpT	ns	ns		
**14**	GRMZM2G064360	Basic endochitinase 1; CHIT4 precursor	5.03	3	1			MpB	2.4	-0.264	additive	0.048
								MpT	7.7	-0.542	additive	0.203
								MpVa	ns	ns		
**15**	GRMZM2G389582	Chitinase; chn3; CHIT5 precursor, SSR umc1492	5.03	3	0			MpB	2.4	-0.264	additive	0.048
								MpT	7.7	-0.542	additive	0.203
								MpVa	ns	ns		
**16**	GRMZM2G129189	Endochitinase PR4; Putative uncharacterized protein	5.05	2	0			MpB	2.6	-0.189	additive	0.091
								MpNC	ns	ns		
								MpT	ns	ns		
								MpVa	ns	ns		
**17**	GRMZM2G057766	Chitinase 1	5.06	28	2			MpT	2.3	ns		
**18**	GRMZM2G141456	Chitinase; Putative uncharacterized protein	5.08	3	2	-73.007	-387.263	MpNC	ns	ns		
					1*	-73.007	-387.263	MpT	2.3	ns		
								MpVa	ns	ns		
**19**	GRMZM2G034598	Beta-hexosaminidase beta chain	6.01	19	5	-29.092	-102.631	MpT	ns			
**20**	GRMZM2G412577	Uncharacterized protein	6.01	0	0			MpT	ns	ns		
								MpVa	3.8	-0.805	dominant	0.070
**21**	GRMZM2G145518	Putative uncharacterized protein	6.01	5	0			MpT	ns	ns		
								MpVa	3.8	-0.805	dominant	0.070
**23**	GRMZM2G145461	Chitinase 2; chitinase candidate L00973; chn*-L00973, pCh2, uiu5 (chn), MZECHITC	6.01	5	0			MpT	ns	ns		
								MpVa	3.8	-0.805	dominant	0.070
**23**	GRMZM2G447795	Xylanase inhibitor protein 1	6.05	5	0			MpB	3.4	0.130	additive	0.086
								MpNC	ns	ns		
								MpVa	2.7	0.858	additive	0.017
**24**	GRMZM2G328171	Xylanase inhibitor protein 1	7.01	24	1			MpB	ns	ns		
	GRMZM2G162359	Putative uncharacterized protein						MpT	ns	ns		
								MpVa	ns	ns		
**25**	GRMZM2G168364	Endochitinase A2; Putative uncharacterized protein	7.03	13	2			MpT	ns	ns		
								MpVa	ns	ns		
**26**	GRMZM2G400497	Uncharacterized protein	8.00	3	1	-5.65	-7.246	MpT	ns	ns		
								MpVa	ns	ns		
**27**	GRMZM2G062974	Basic Endochitinase A	8.03	10	0			MpB	ns	ns		
								MpNC	ns	ns		
								MpT	ns	ns		
								MpVa	ns	ns		
**28**	GRMZM2G083292	Putative uncharacterized protein	8.03	5	3			MpB	2.4	0.170	additive	0.074
								MpT	ns	ns		
**29**	GRMZM2G037694	Hydrolase, hydrolyzing O-glycosyl compound, Chitinase; Putative uncharacterized protein	8.03	0	0			MpB	2.4	0.170	additive	0.074
								MpNC	ns	ns		
								MpT	ns	ns		
								MpVa	ns	ns		
**30**	GRMZM2G117405	Beta-hexosaminidase	8.03	17	11	52.308	246.477	MpB	2.4	0.170	additive	0.074
					1*	52.308	246.477	MpNC	ns	ns		
								MpT	ns	ns		
								MpVa	ns	ns		
**31**	GRMZM2G400999	Xylanase inhibitor protein 1	10.0	11	0			MpT	ns	ns		
								MpVa	ns	ns		
**32**	GRMZM2G090441	Chitinase; Putative uncharacterized protein	10.04	4	0			MpNC	ns	ns		
								MpT	ns	ns		
								MpVa	ns	ns		
**33**	GRMZM2G005633	Endochitinase B Precursor; Fragment	10.04	12	0			MpNC	ns	ns		
								MpT	ns	ns		
								MpVa	ns	ns		

Number of markers tested, number of associations of p < 10^–3^ (and p < 10^–4^, indicated with an asterisk) as calculated by MLM, the effect of the beneficial allele based on association mapping, the mapping populations in which at least one marker segregated and was mapped, the significance of the linkage mapping (LOD), the effect of the beneficial allele based on QTL mapping, the gene action, and R^2^, the maximum percentage of phenotypic variation explained by the QTL mapped, are all noted.

Two other QTL were also identified with high LOD scores from the mapping of chitinase gene sequences (S2 Fig). GRMZM2G051921, GRMZM2G051943 and/or GRMZM2G052175 were associated with LOD scores up to 5.4 in two mapping populations. Although this QTL had been published previously [24, 27] it was not known that a chitinase gene may be the causative locus, and the current study allowed the QTL interval to be mapped to a smaller genetic window. The three genes, in bin 2.04, were only 30 Kb apart, and could not be distinguished in linkage mapping with populations of the size reported here (less than 300 individuals each). One or more of them may be responsible for increased aflatoxin accumulation resistance. The polymorphisms used to map the QTL included one SNP, two InDels, and one SSR, that fell within the sequences of GRMZM2G051943 and GRMZM2G052175. The resistance was associated with additive gene action in both populations and came from the resistant line Mp313E, and the QTL explained 24.1% of the phenotypic variation. The other QTL was centered over GRMZM2G064360, GRMZM2G389582 or GRMZM2G389557 in bin 5.03. This was also directly under the peak of a QTL published previously in two different mapping populations [25, 27], but again, it was not known that a chitinase could be the causative locus. This QTL was mapped with two SNPs within GRMZM2G064360 and one SSR from within the sequence of GRMZM2G389582, and because these 3 genes were all within a 48 Kb region, their genetic effect was indistinguishable. This QTL was found in population Mp715 x T173 with a LOD score of 7.7, an additive gene action, and explained 20.3% of the phenotypic variation. Resistance came from the resistant parent Mp715. A QTL in the same region was found in the Mp313E x B73 mapping population with a LOD of 2.4, also with additive gene action from the Mp313E resistant line, and explained 4.8% of the phenotypic variation.

Eight other QTL mapped within or very near other chitinase gene sequences in this study (S2 Fig). These QTL were not mapped with a LOD greater than 5, explained less than 5% of the phenotypic variation, were not reported in more than one mapping population, were not supported by association results, and/or were associated with dominant gene action, which is not as useful for plant breeding. These eight genes were GRMZM2G162505, GRMZM2G403475, GRMZM5G837822, GRMZM2G133781, GRMZM2G129189. GRMZM2G099454, GRMZM2G447795, and GRMZM2G117405. Resistance came from the susceptible parent in the last three cases. Information on all these additional QTL are reported in Table 3, and they may still be useful for plant breeding, especially if the three QTL of larger phenotypic effect and LOD scores have already been exploited in a breeding population.

**Fig 4:**
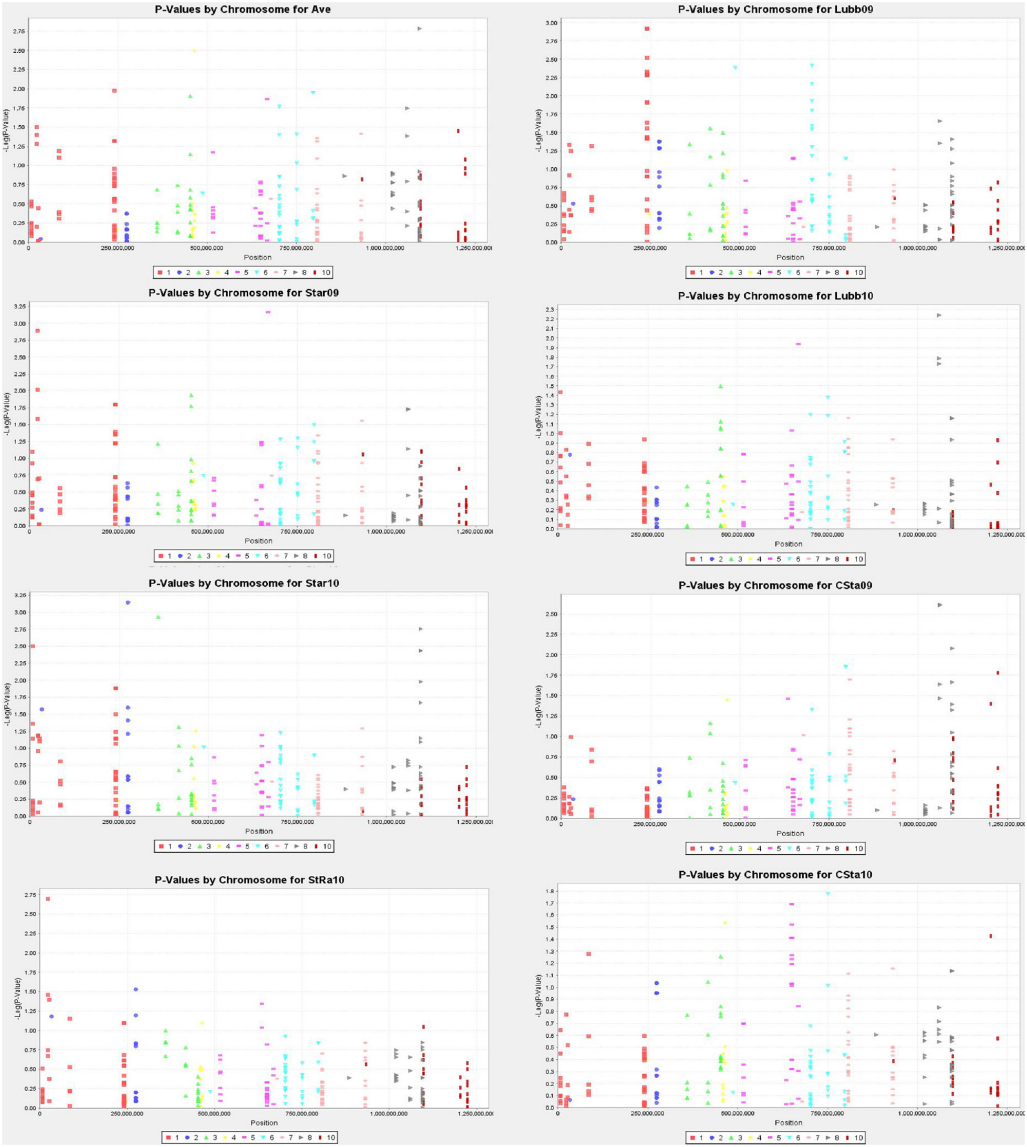
Manhattan graph generated from the association analysis of SNPs extracted from the genetic sequence of 30 chitinase genes via TASSEL. Each graph depicts the untransformed aflatoxin means in one environment, or averaged over all environments (Ave). Star09 = Starkville, 2009; Star10 = Starkville 2010; StRa10 = Starkville, Raymond site, 2010; Lubb09 = Lubbock, 2009; Lubb10 = Lubbock, 2010; CSta09 = College Station 2009; CSta10 = College Station 2010.

For association analysis, there were 66 SNP-trait associations involving 15 chitinase candidate genes identified with the mixed linear model (MLM, 8.98x10^-5^≤ p ≤ 9.60x10^-3^; Table 3; Fig 4). No SNPs could be found within 30 Kb of the sequence of GRMZM2G037694 or GRMZM2G412577, and only two SNPs within 30 Kb of GRMZM2G129189; therefore, conclusions cannot be made concerning these three genes via association analysis. At lower p values (below 1x10^-4^), five genes (GRMZM2G312226, GRMZM2G051921, GRMZM2G358153, GRMZM2G141456 and GRMZM2G117405) still show associations in one or more environments. The SNP in GRMZM2G051921 was supported by a QTL in two populations mapping at LOD scores of 5.0 and 4.8 from the resistant parent Mp313E. The associations with GRMZM2G358153 and GRMZM2G117405 were supported by QTL mapping at LOD scores of 2.4, both in MpB. The SNP from GRMZM2G141456 co-localized with a QTL in one population, but with a LOD of only 2.3, just below the critical threshold level. The SNP in GRMZM2G312226 was not supported by any QTL.

### Evolution of sequences

**Fig 5:**
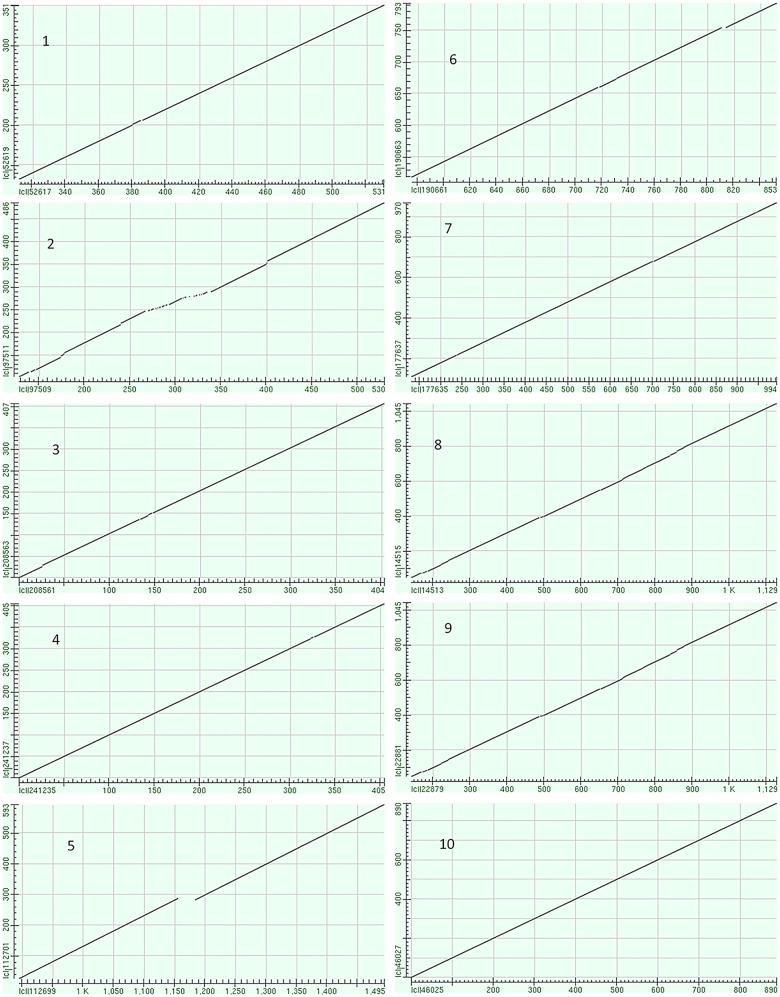
Dot matrix view of paired alignments of maize chitinase exon sequences with similar gene structures, based on the dendogram in Fig 1. 1 = GRMZM2G051943 (Exon 1) vs. GRMZM2G052175 (exons 1 and 2) (86% identical); 2 = GRMZM2G051943 (Exon 1) vs. GRMZM2G051921 (exon 1) (77% identical); 3 = GRMZM2G051943 (Exon 2) vs. GRMZM2G052175 (exon 3) (85% identical); 4 = GRMZM2G051943 (Exon 2) vs. GRMZM2G051921 (exon 2) (87% identical); 5 = Exons 1 and 2 GRMZM2G412577 vs. GRMZM2G400497 (95% identical); 6 = Exons 4 and 5 of GRMZM2G099454 vs. exons 1–3 of GRMZM2G103668 (78% identical); 7 = GRMZM2G057093 VS GRMZM2G162505 (88% identical); 8 = GRMZM2G162359 VS GRMZM2G328171 (76% identical); 9 = GRMZM2G400999 VS GRMZM2G447795 (81% identical); 10 = GRMZM2G080547 VS GRMZM2G160265 (98% identical).

Several of the chitinase genes that were very close to each other appeared to be recent duplication events. Genes GRMZM2G051921, GRMZM2G051943 (ChiA), GRMZM2G052175, and GRMZM2G122708 all lay within a range of 50 Kb on chromosome 2. The first three genes shared significant sequence similarity and were used in a comparison analysis. The exon sequences of the three genes were aligned in pair-wise comparisons to generate a dot matrix view of similarity using the bl2seq specialized BLAST option from NCBI [36] (Fig 5). GRMZM2G051921 and GRMZM2G051943 have 2 exons, while GRMZM2G052175 has 3. The first two exons of GRMZM2G052175 and the first exon of GRMZM2G051943 were 86% similar. The third exon of GRMZM2G052175 and the second exon of GRMZM2G051943 were over 85% similar. The intronic sequence between the first two exons of GRMZM2G052175 was blasted against the Maize Transposable Elements Database (TEDB, [55]) and showed significant alignment with the transposable element RLC_gudyeg_AC206942-9404 (Expect = e-102, Identities = 374/437 (85%)). It is therefore suggested that GRMZM2G052175 was a recent duplication of GRMZM2G051943 followed (or caused) by transposable element insertion into the first exon. The first exon of gene GRMZM2G051921 is over 75% similar to the first exon of GRMZM2G051943, and the second exons were over 85% similar. GRMZM2G051921 was thus also suspected to be a duplication of GRMZM2G051943, perhaps independently of the insertion of the TE that led to GRMZM2G052175. Both genes GRMZM2G051921 and GRMZM2G051943 have an identical expression pattern (S1 Fig); the expression data for GRMZM2G052175 appears in the coleoptiles at 6 days after silking versus expression in the germinating seed for GRMZM2G051921 and GRMZM2G051943.

The exons of other chitinase genes which appear to be closely related by the phylogenetic dendogram (Fig 1) were also compared by alignment using the bl2seq Blast option from NCBI [36]. GRMZM2G057093 AND GRMZM2G162505 are 88% identical (Fig 5). These two genes lie on opposite strands of the DNA in nearly the same position on chromosome 1, Bin 1.08 (Table 1). GRMZM2G162359 and GRMZM2G328171 are 76% identical and lie on opposite strands of chromosome 7, bin 7.01 (Table 1). These pairs of genes may be the result of recent duplication events, possibly mediated by transposable elements. Exons 4 and 5 of GRMZM2G099454 are 78% identical to exons 1–3 of GRMZM2G103668; these genes are both on chromosome 1 but in different bins 1.01 and 1.05, respectively. The exons of GRMZM2G412577 and GRMZM2G400497 are 95% identical (Fig 5); these genes lie on different chromosomes, (6 and 8, respectively). Because these two gene pairs are not tandemly arranged nor fall within known duplicated segments of the maize genome, it is unknown why they show such high similarities. Similarities between other chitinase family genes are less than 75%.

## Discussion

A genomewide atlas of chitinase transcription during maize development was generated using expression data from two different sources [39–41] (S1 Fig). Data from these expression studies may provide clues as to how the chitinase genes increase *A*. *flavus* resistance. For example, gene GRMZM2G145461 was highly expressed in silk and husk tissue, and may be a defense for the developing ear against corn ear worm and fall armyworm, which enter the developing ear through the silk channel or directly through the husk. At the same time, the feeding insects may introduce *A*. *flavus* into the ear and are known vectors of the fungus. This gene was not identified as highly associated with resistance to aflatoxin accumulation or *A*. *flavus*, and only moderately linked to resistance in one QTL mapping population; however, since all phenotyping of these populations involved the direct injection of spores into the developing ear, any silk or husk resistance against the fungus contributed by this gene would have been overcome and thus not measured in this study. Genes GRMZM2G057766 and GRMZM2G083292 were active primarily in roots, and may provide defense against corn root worm, nematodes, and/or soil borne pathogenic fungi. These two genes were also not associated nor linked to aflatoxin levels in corn grain, and based on the tissues in which they were expressed, were not expected to be.

On the other hand, gene GRMZM2G312226 was constitutively expressed at high levels in all maize tissues studied. A SNP in GRMZM2G312226 was the second most highly associated with aflatoxin levels in the grain. Maize lines with the resistant allele of this SNP had aflatoxin levels equal to the mean of all lines in the panel, while lines with the susceptible allele of the SNP had highly increased levels of toxin (Table 3). This suggested that the susceptible SNP corresponded to an allele that interfered with the gene function of GRMZM2G312226 and reduced the background protection provided by constitutive expression. Genes GRMZM2G103668, GRMZM2G051921/ GRMZM2G052175, and GRMZM2G064360/ GRMZM2G389582/ GRMZM2G389557 were associated or linked to increased resistance. These genes were expressed very little or not at all in any tissues measured in [39–41] and the beneficial allele may be increasing gene expression in resistant lines only (which were, unfortunately, not included in the expression atlas study) to increase resistance following inoculation by *A*. *flavus* spores. This possibility would have to be investigated in a new gene expression study of resistant lines. Genes GRMZM2G051943 (highly expressed in germinating seed but not developing seed), GRMZM2G141456 (somewhat expressed in germinating seed and immature leaves) and GRMZM2G117405 (highly expressed in anthers and tassels) may likewise owe their increased resistance to a mechanism specific to these tissues.

Some of the results observed in the present study agree with previous reports. Germinating maize embryos have shown an induction of two acidic chitinase isozymes (no specific gene or protein identifier reported) in response to infection by the fungus *F*. *moniliforme* [15]. A chitinase isolated from mature seeds of the *A*. *flavus* resistant line Tex6 inhibits the growth of *A*. *flavus* [56]. Although the exact identity of this chitinase was not specified, it was highly similar to the homologous chitinases A and B. Commercial hybrids have been shown to produce two different forms of the ChitA (GRMZM2G051943) and the Chit B (GRMZM2G005633) proteins, due to either difference in the genetic sequences or post-translational modifications [57, 58]. Both forms of ChitA and ChitB appear to be modified by proteases from the fungi *Bipolaris zeicola*, *Stenocarpella maydis* and *Fusarium verticillioides*; this leads to a reduction of chitinase function and allows the fungi to overcome host barriers [57–59]. A chitinase A gene in bin 2.04 (GRMZM2G051943 and/or GRMZM2G052175, thus possibly ChitA) was associated with a large aflatoxin reducing QTL in the present study.

The effect of the beneficial allele of some of the genes mapped in this study was large enough to justify the creation of near isogenic or transgenic lines, or knock-out mutants, to verify gene effect in an independent background. Validation of these genes in independent tests and genetic backgrounds is now underway. The QTL at GRMZM2G103668 in bin 1.05 (at LOD 9.2) explained 18% of the phenotypic variation for aflatoxin level in the mapping population. The QTL in bin 2.04 attributable to one of the linked genes (at LOD 5.5) explains 24% of the phenotypic variation in one population and 9% in a second, related population. Finally, the QTL in bin 5.03 attributable to one of three linked genes (at LOD 7.7) explains 20% of the phenotypic variation in one population and 5% in an unrelated population. The QTL at 2.04 and 5.03 have been seen before in related germplasm, and represent stable resistance factors that are useful across environments. The QTL at 1.05 is new, and must be further verified before stable resistance can be ensured. These QTL are certainly of large enough phenotypic effect to attempt to backcross into susceptible varieties, singly or together, in an attempt to increase resistance, and near isogenic lines are currently being created using the SNP and SSR markers within the genes (Table 2).

As measured by antifungal activity, endochitinases have greater activity than exochitinases; however, the enzymes are synergistic. Thus a combination of exo- and endochitinases is several-fold more active than any single enzyme [60]. The most significantly associated SNPs from QTL mapping and also from the association analysis may be pyramided via SNPs reported in Tables 2 and 3 and may boost resistance even higher than expected from the phenotypic effect calculated from single genes. Markers for marker assisted selection of genes identified via association analysis include the SNPs in the genes in bin 1.02 (p = 3.78E-04, R^2^ = 5%); 2.04 (p = 7.24E-04, R^2^ = 5%, already suggested above due to QTL information); 4.03 (p = 8.98E-05, R^2^ = 6%); 5.08 (p = 6.84E-04, R^2^ = 4%); and 8.03 (p = 9.22E-04, R^2^ = 4%). The frequency of the resistance-contributing allele can be seen in Table 3. This information can give some indication as to the utility of each of these SNPs in a general breeding program, as alleles already very common in the population may be less useful in general, although may help increase resistance in specific genetic backgrounds.

## Supporting Information

S1 Fig Genome wide atlas of chitinase transcription during maize development adapted from Sekhon et al. [40] and Qteller [39] sorted by chromosomal position for each gene listed in Table 1. a = Similar Expression information from Qteller; b = Qteller indicates greater expression in the undifferentiated ear, appears to be constitutively expressed; c = Expression levels are very low from the Qteller output; d = Very low expression in Qteller; e = Qteller indicates high expression levels in the ear, silks, tassel, and/ or roots; f = Conflicting results, appears to be constitutive in Sekhon, but more expressed in the seeds in Qteller. Expression levels are low for both; g = Not studied in Qteller; h = Not studied by Sekhon et al.
(DOCX)

S2 Fig Linkage maps of four QTL mapping populations showing the QTL reported previously and new QTL attributed to the chitinase genes characterized in this study. The mapping populations, and previous references for them, are: MpT = Mp715 x T173 (Warburton et al., 2011); MpB = Mp313E x B73 (Brooks et al., 2005); MpVa = Mp313E x Va35 (Willcox et al., 2013); MpNC = Mp717 x NC300 (Warburton et al., 2009). New markers used to test the chitinase genes are shown with a red circle.
(DOCX)

S1 Table Gene Ontology and other sequence identifiers and associated information, including those from protein, messenger RNA, and pathway databases. (XLSX)

S2 Table Full length genomic and protein sequences from the MaizeGDB [27] and Uniprot [31, 33] databases. (XLSX)

S3 Table All SNPs extracted from the sequence of the chitinase genes within the association mapping panel. (XLSX)
