# Supplementary material for: Characterization of the Maize Chitinase Genes and Their Effect on Aspergillus flavus and Aflatoxin Accumulation Resistance
Source: PLoS One. 2015 Jun 19;10(6):e0126185. doi: 10.1371/journal.pone.0126185 (PMC4475072; doi:10.1371/journal.pone.0126185)

## Supplemental Figure 1: Genome wide atlas of chitinase transcription during maize development adapted from Sekhon et al. (37) and Qteller (36) sorted by chromosomal position.

a= Similar Expression information from Qteller; b= Qteller indicates greater expression in the undifferentiated ear, appears to be constitutively expressed; c=Expression levels are very low from the Qteller output; d=Very low expression in Qteller; e=Qteller indicates high expression levels in the ear, silks, tassel, and/ or roots; f= Conflicting results, appears to be constitutive in Sekhon, but more expressed in the seeds in Qteller. Expression levels are low for both; g=Not studied in Qteller; h= Not studied by Sekhon et al.

## Chromosome 1

#### GRMZM2G099454^a^


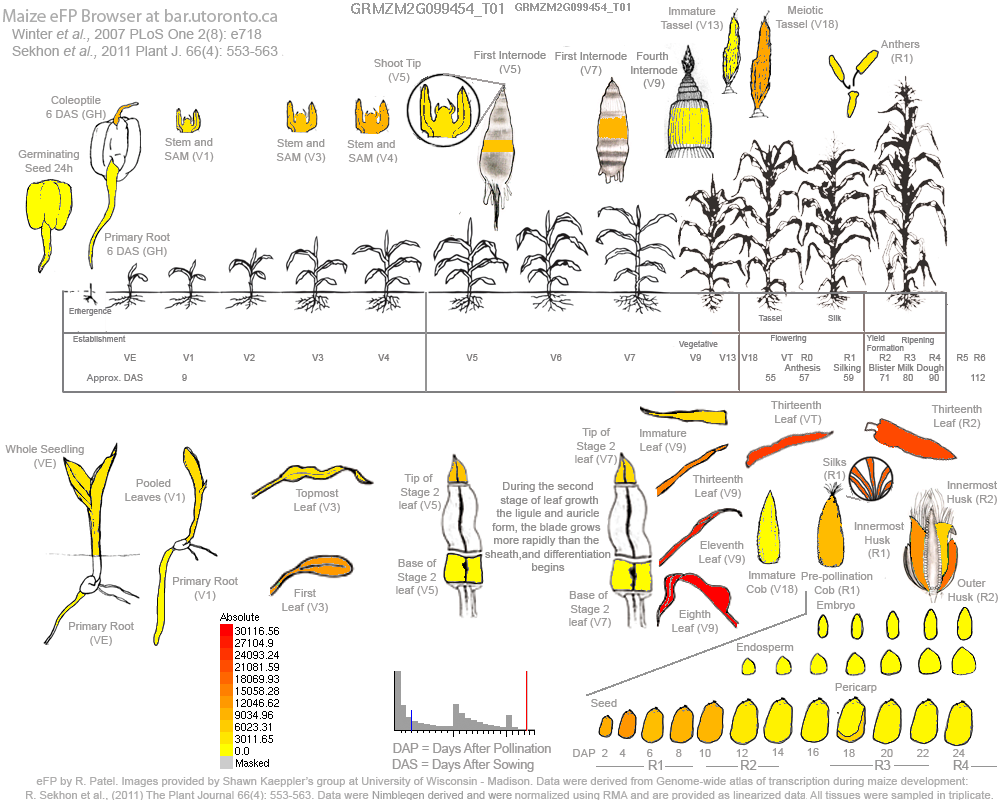


#### GRMZM2G312226^b^


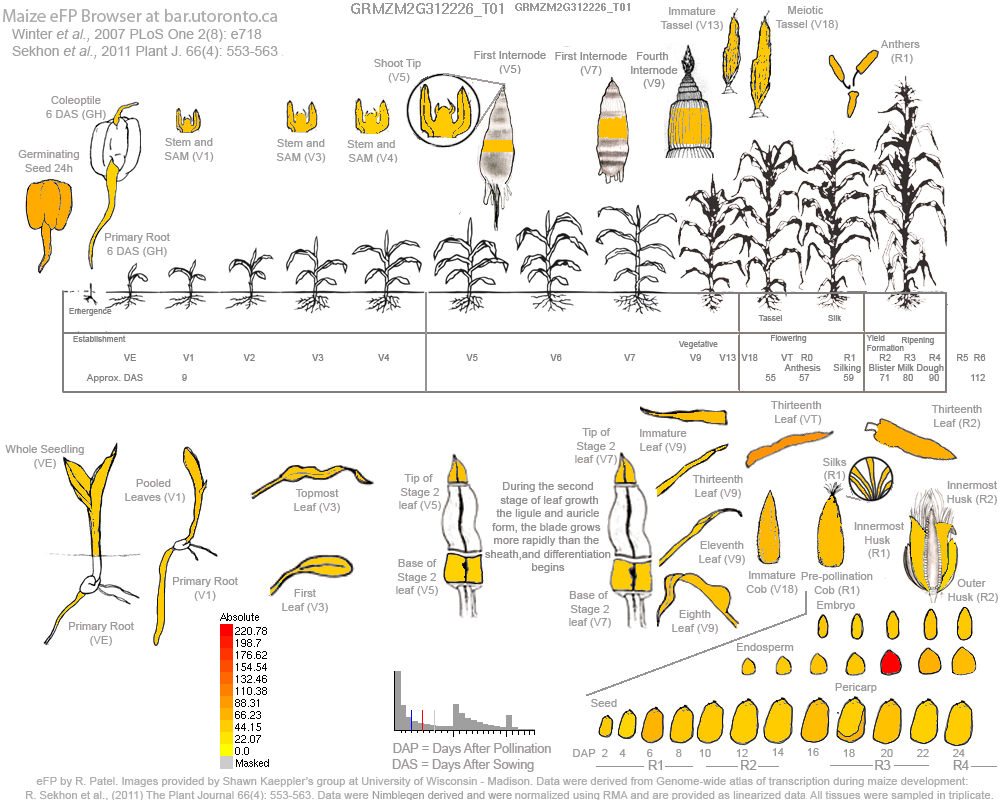


#### GRMZM2G134251^a^


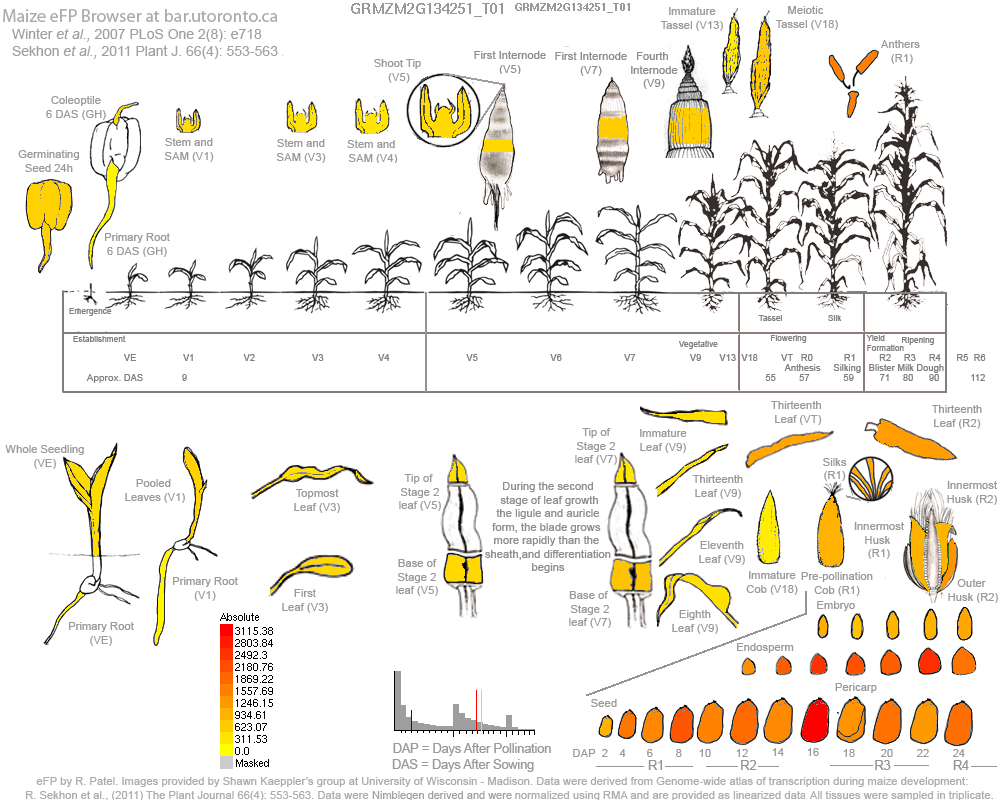


#### GRMZM2G103668^c^


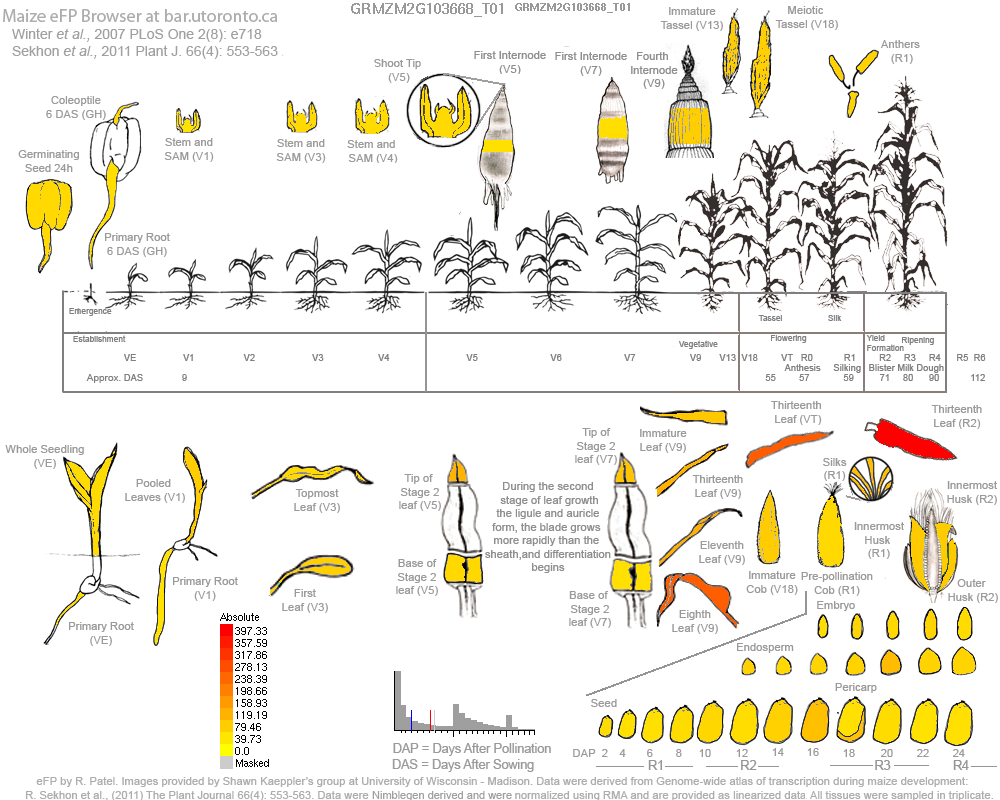


#### GRMZM2G544531^gh^

#### GRMZM2G162505^a^


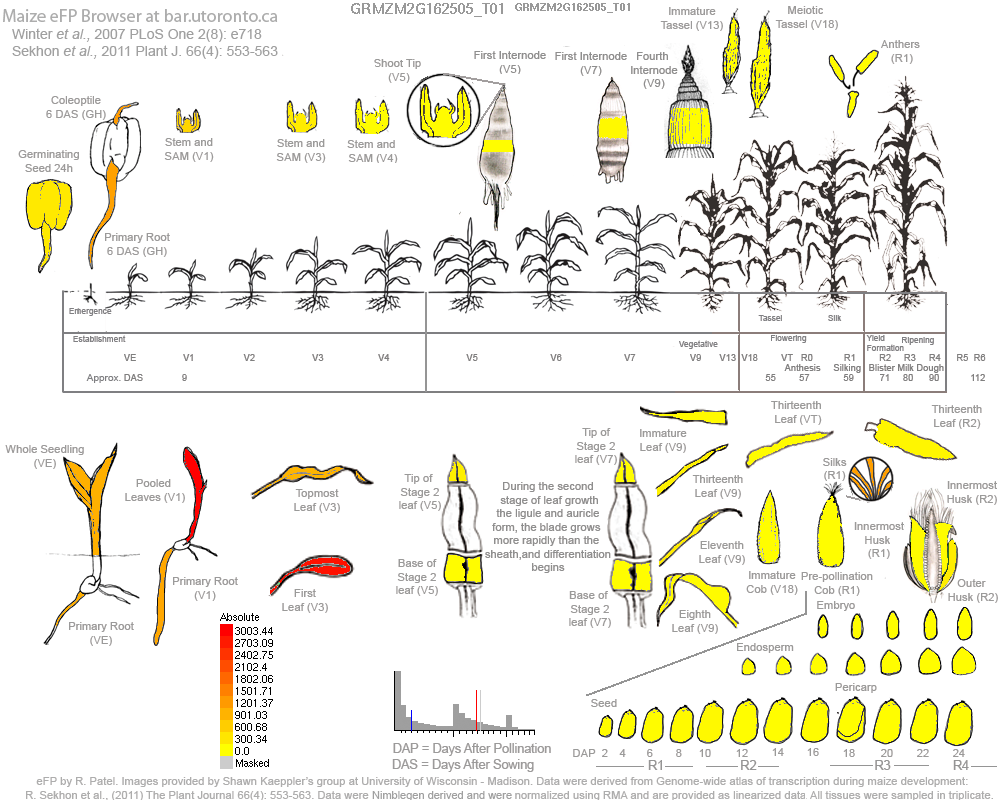


#### GRMZM2G057093^a^


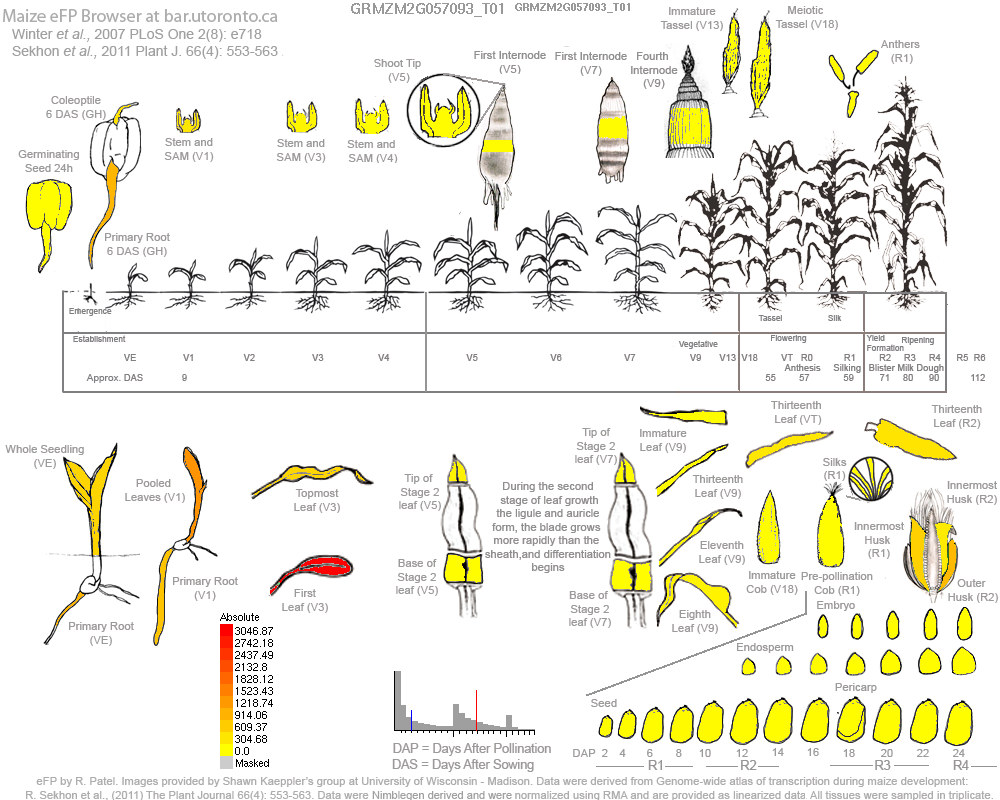


## Chromosome 2

#### GRMZM2G051921^d^


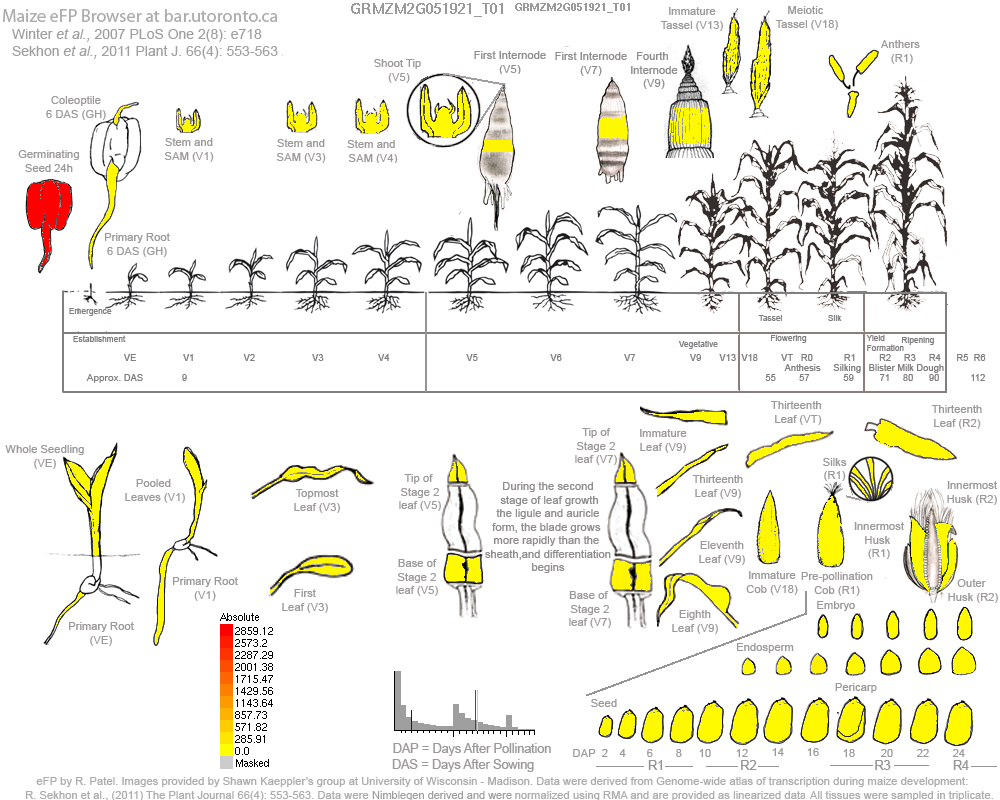


#### GRMZM2G051943^e^


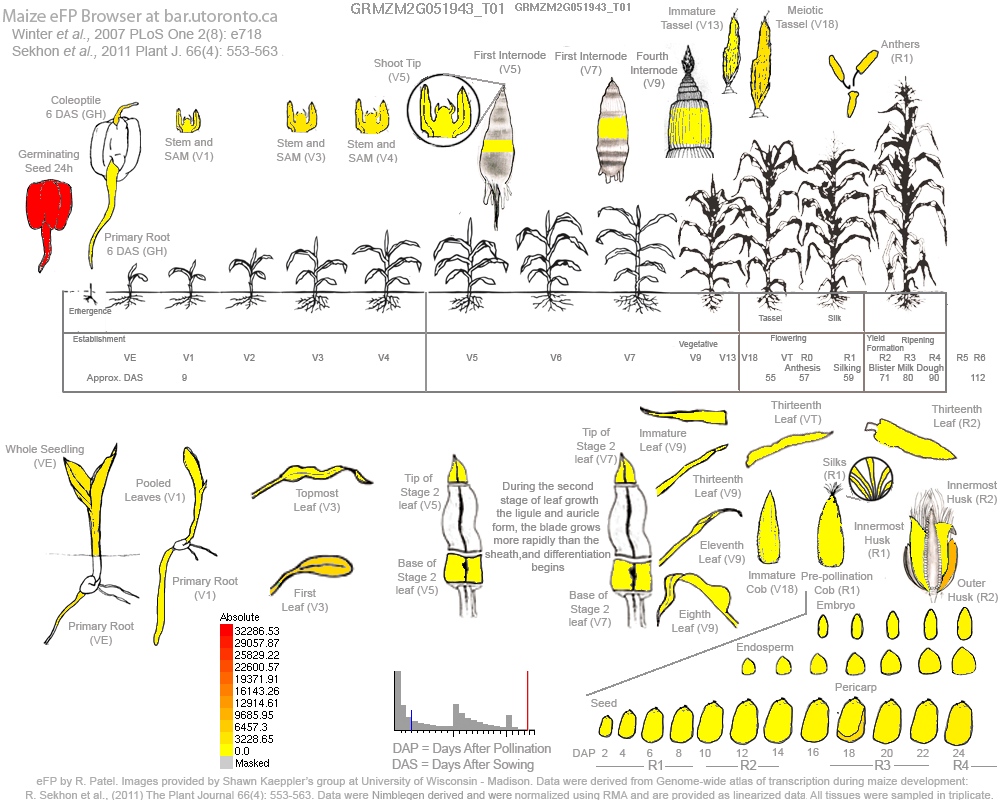


#### GRMZM2G052175^d^


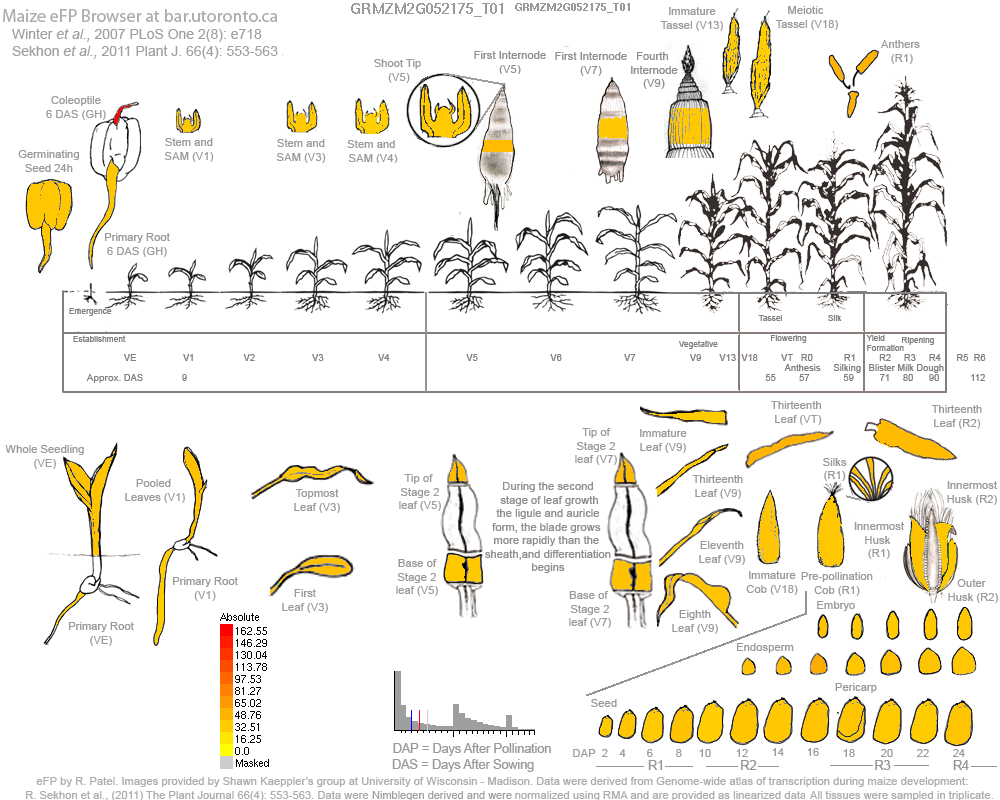


## Chromosome 3

#### GRMZM2G403475^f^


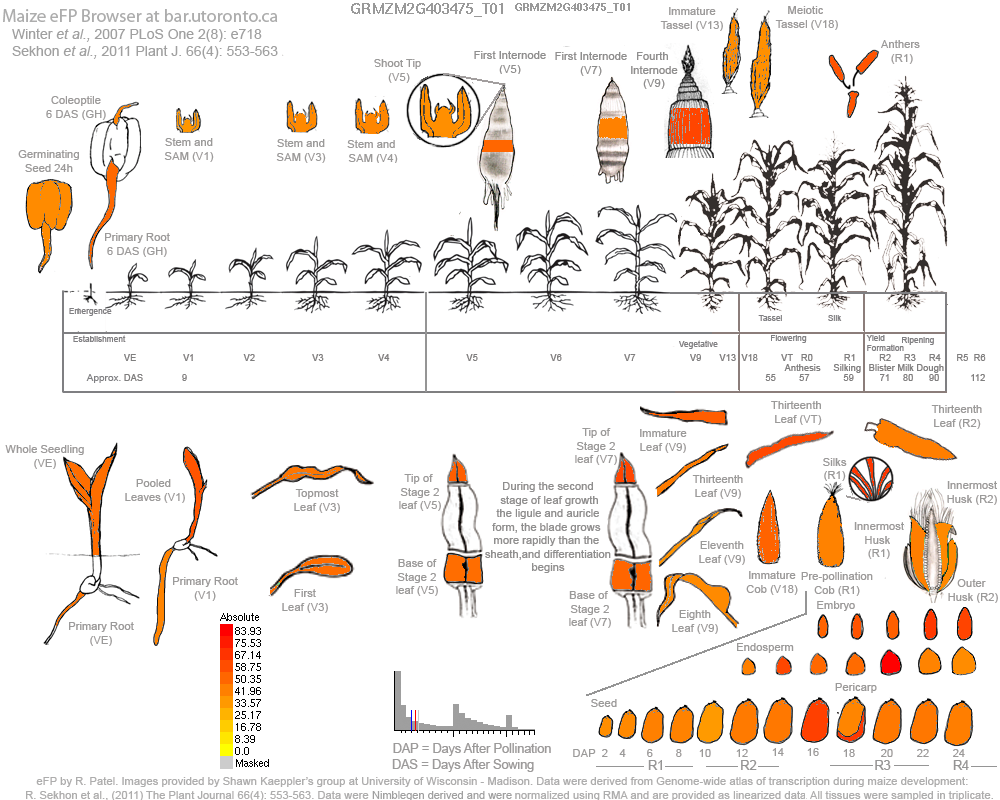


#### GRMZM5G837822^a^


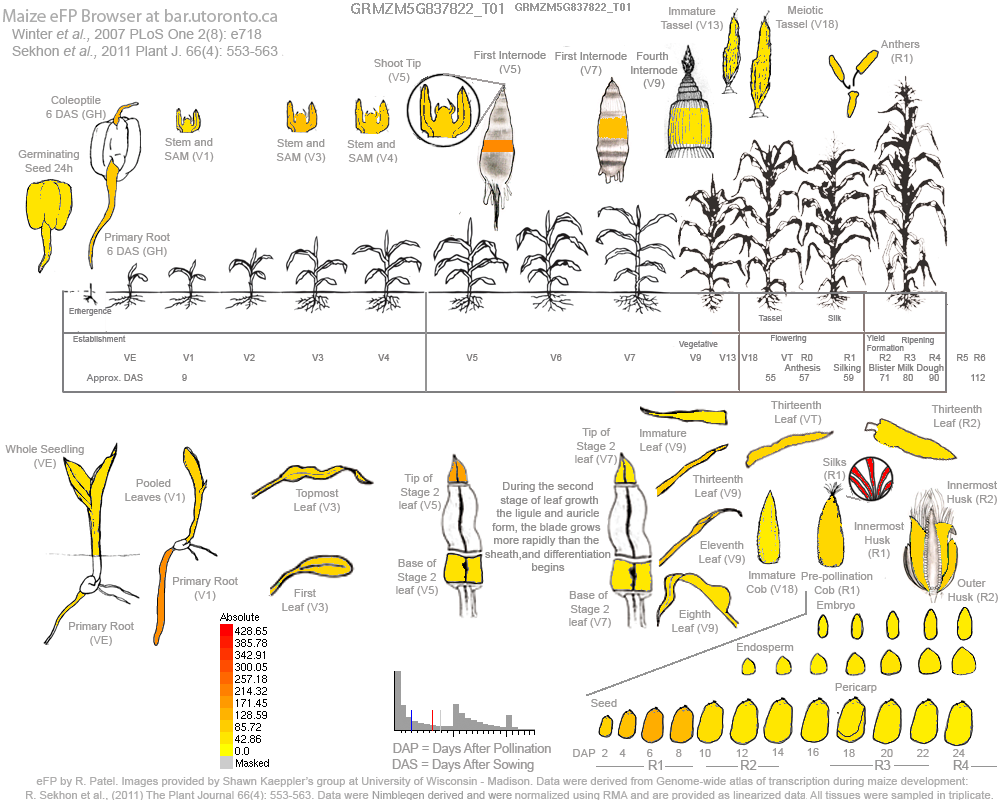


#### GRMZM2G430936^g^


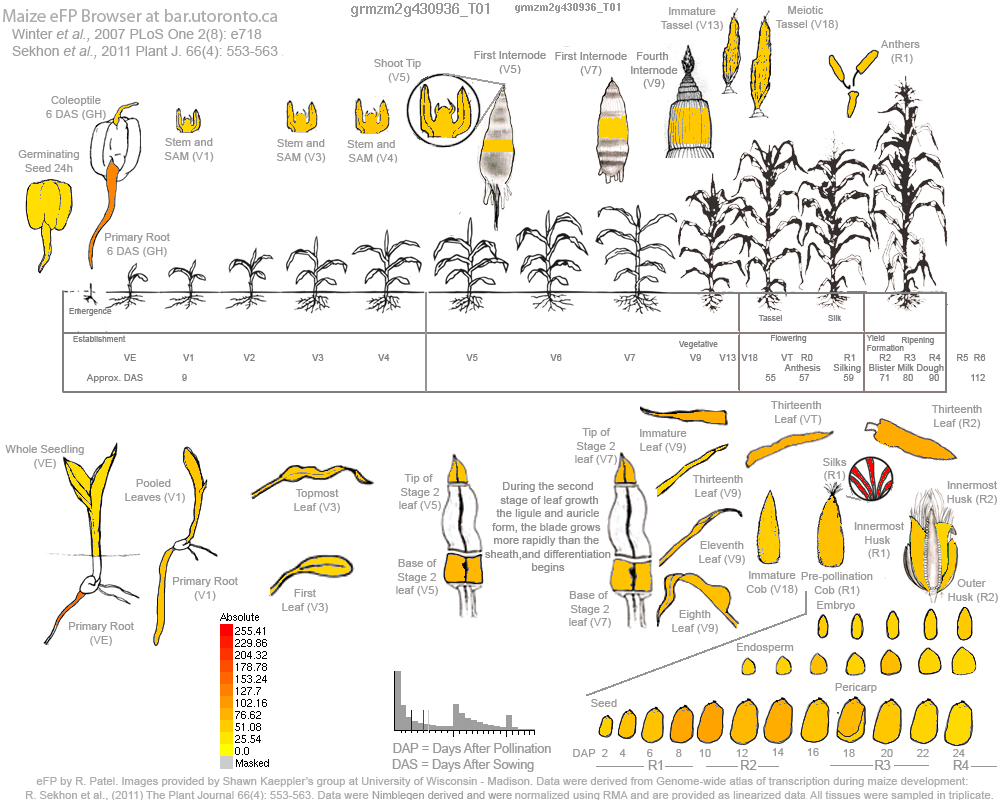


#### GRMZM2G430942^g^


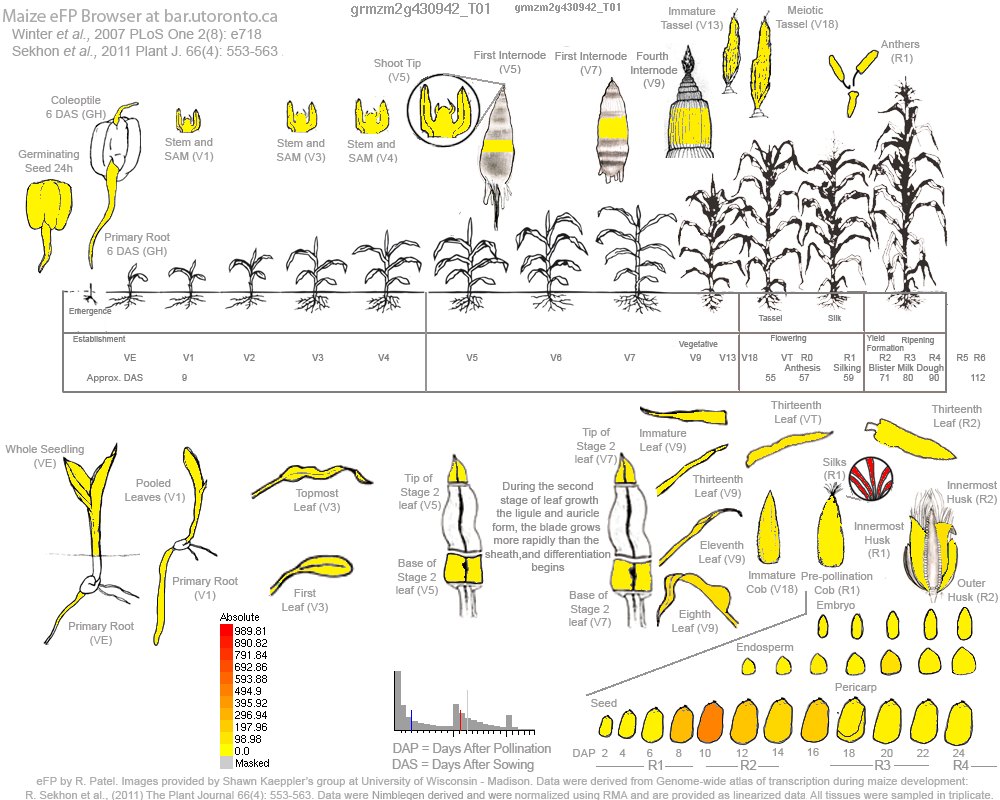


#### GRMZM2G023650^g^


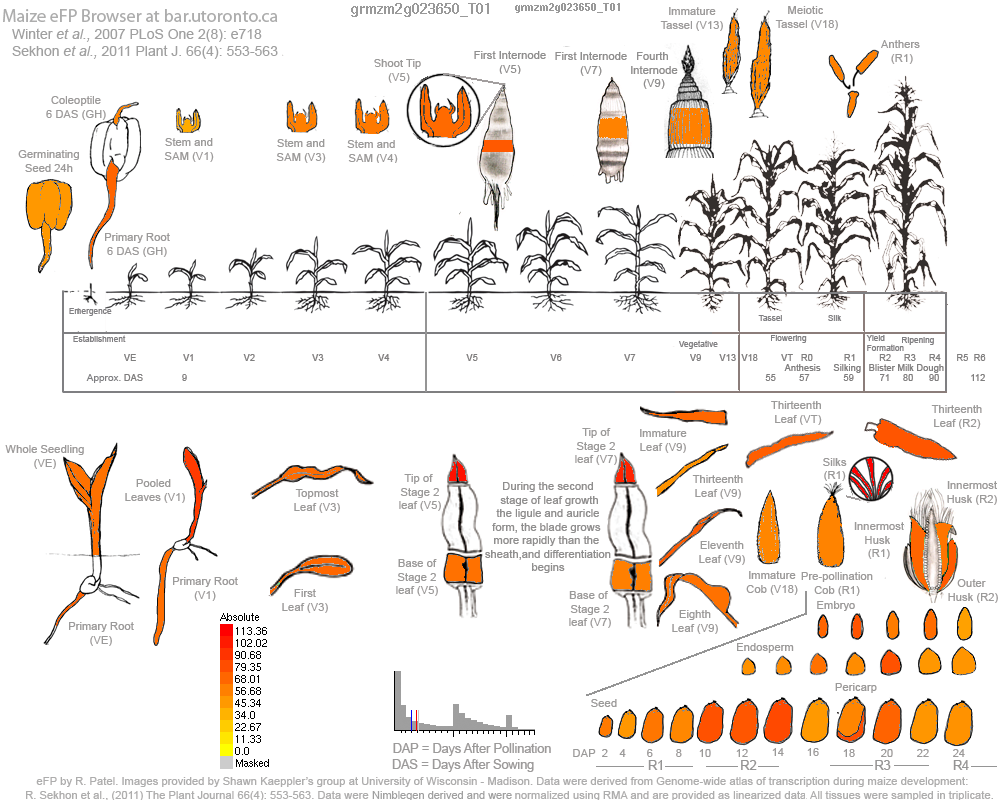


#### GRMZM2G453805^a^


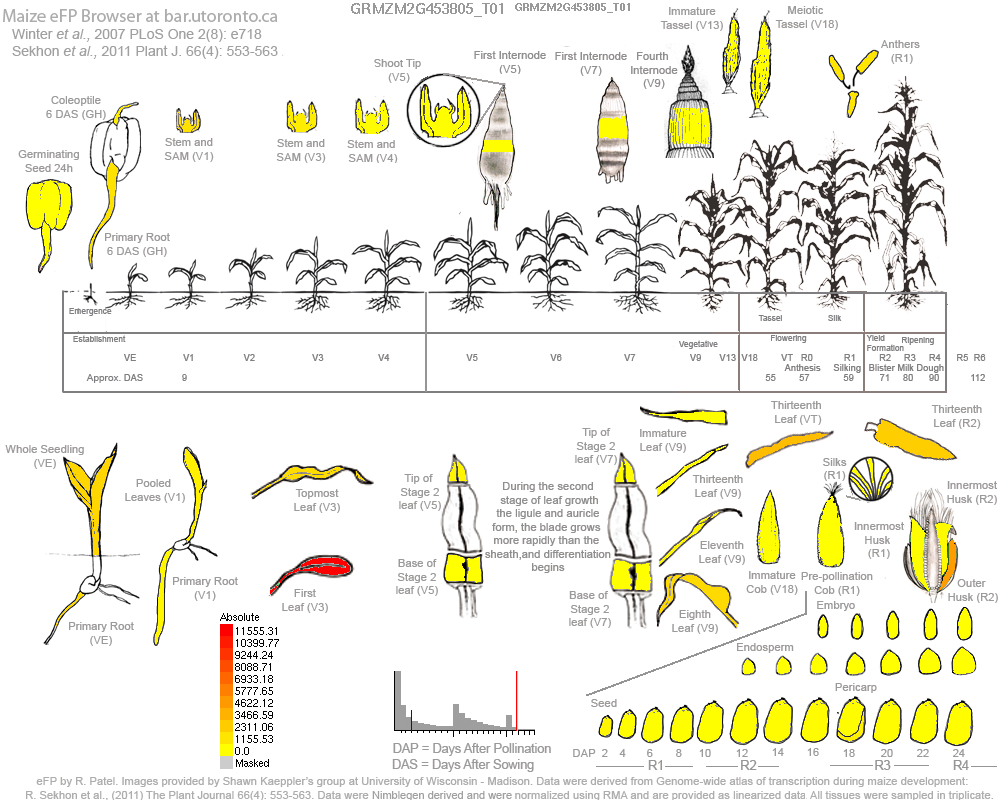


## Chromosome 4

#### GRMZM2G133781^e^


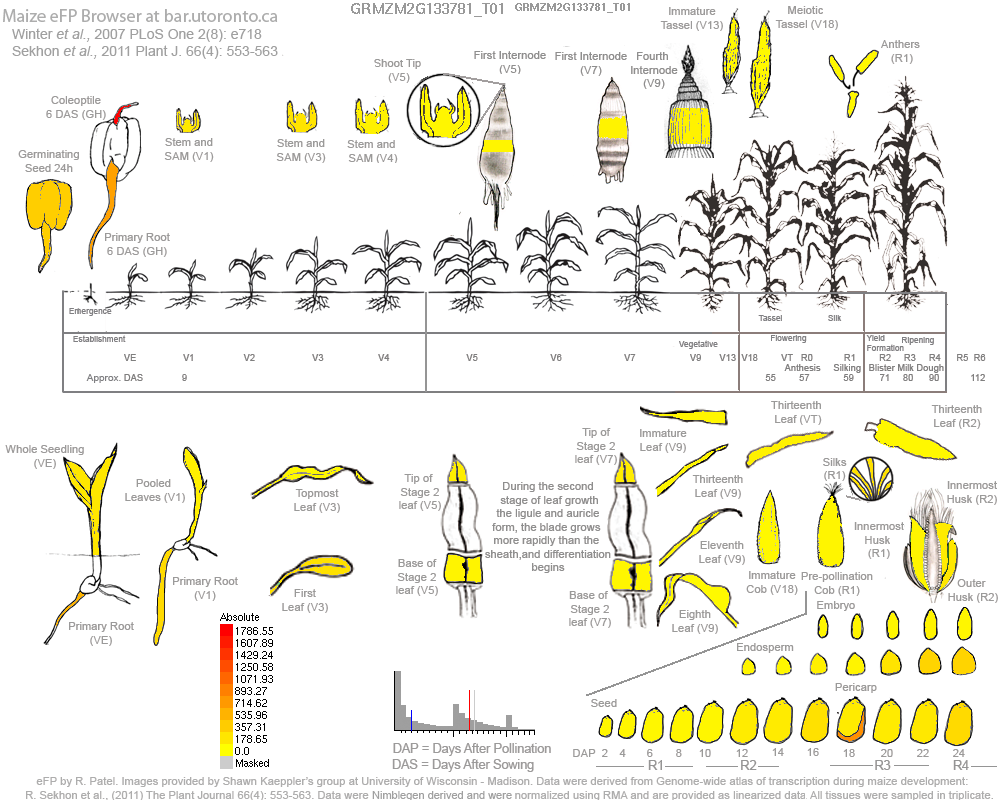


#### GRMZM2G358153^g^


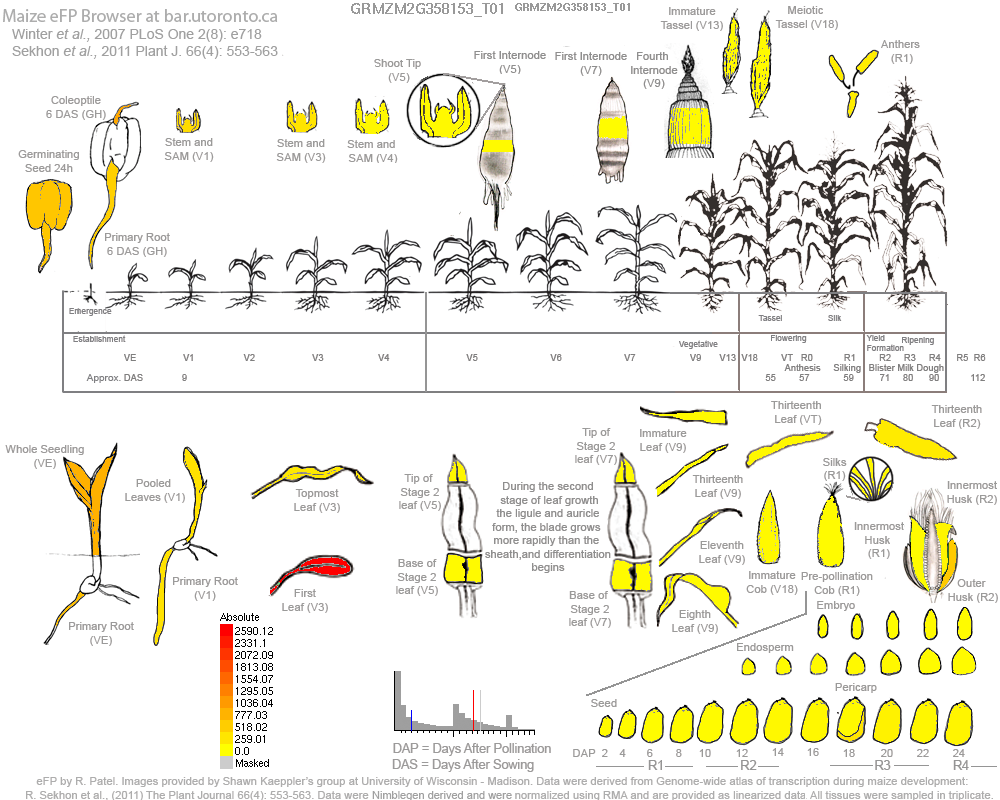


## Chromosome 5

#### GRMZM2G064360^a^


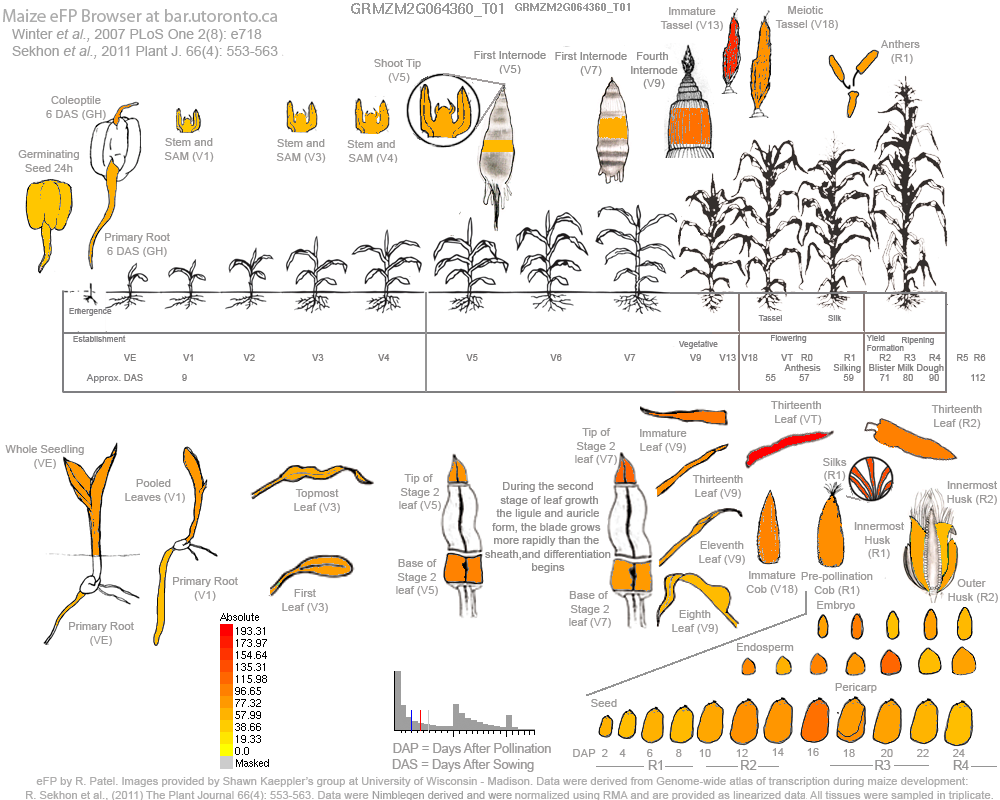


#### GRMZM2G389582^a^


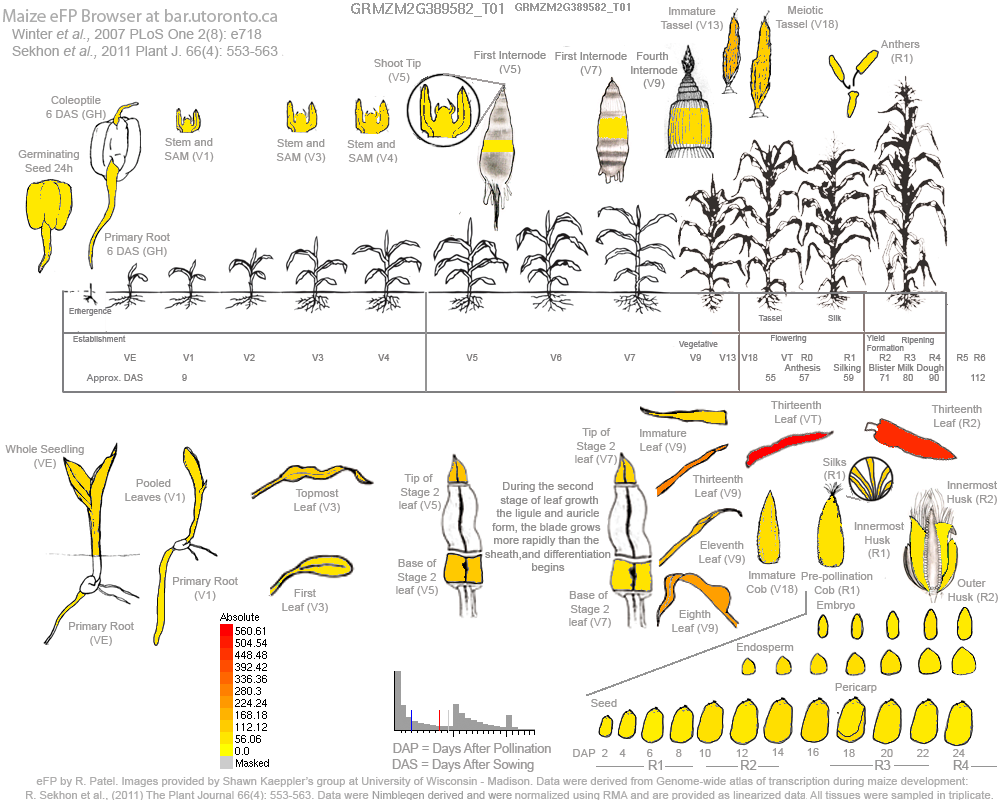


#### GRMZM2G129189^a^


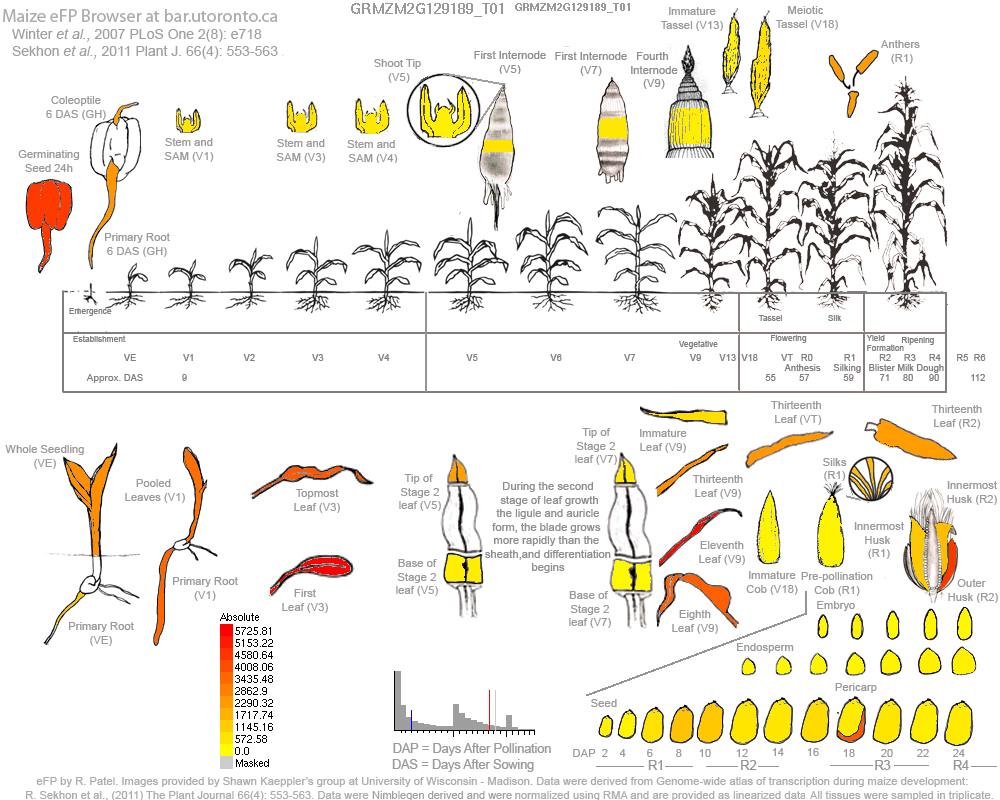


#### GRMZM2G389577^g^


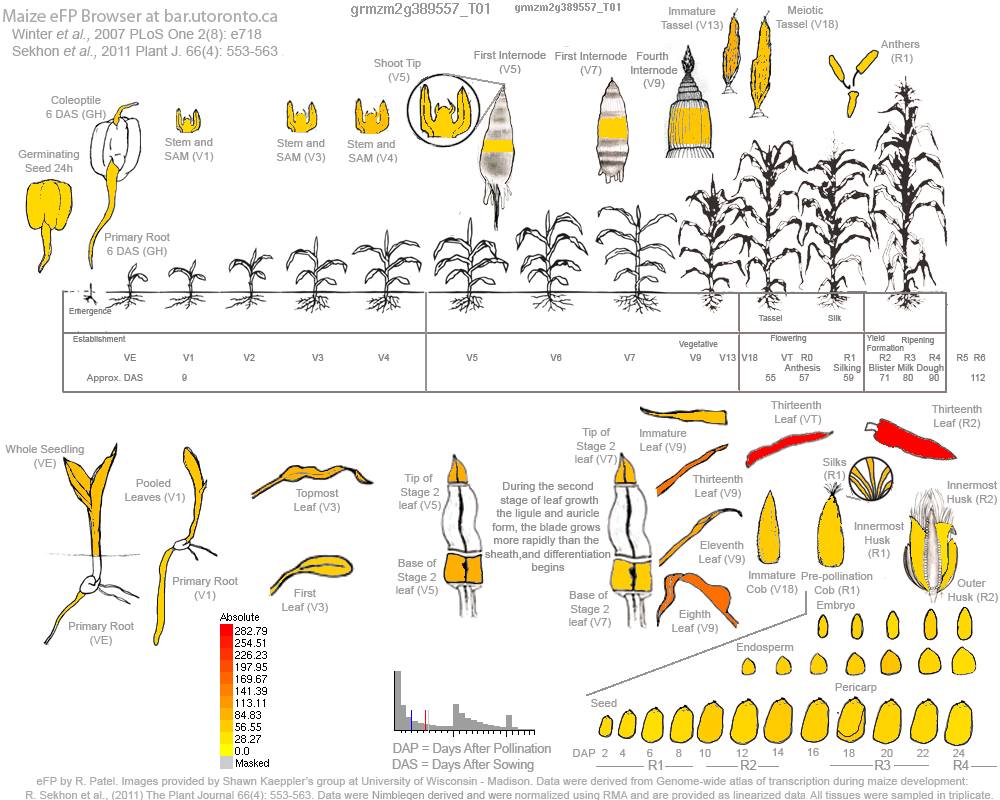


#### GRMZM2G057766^a^


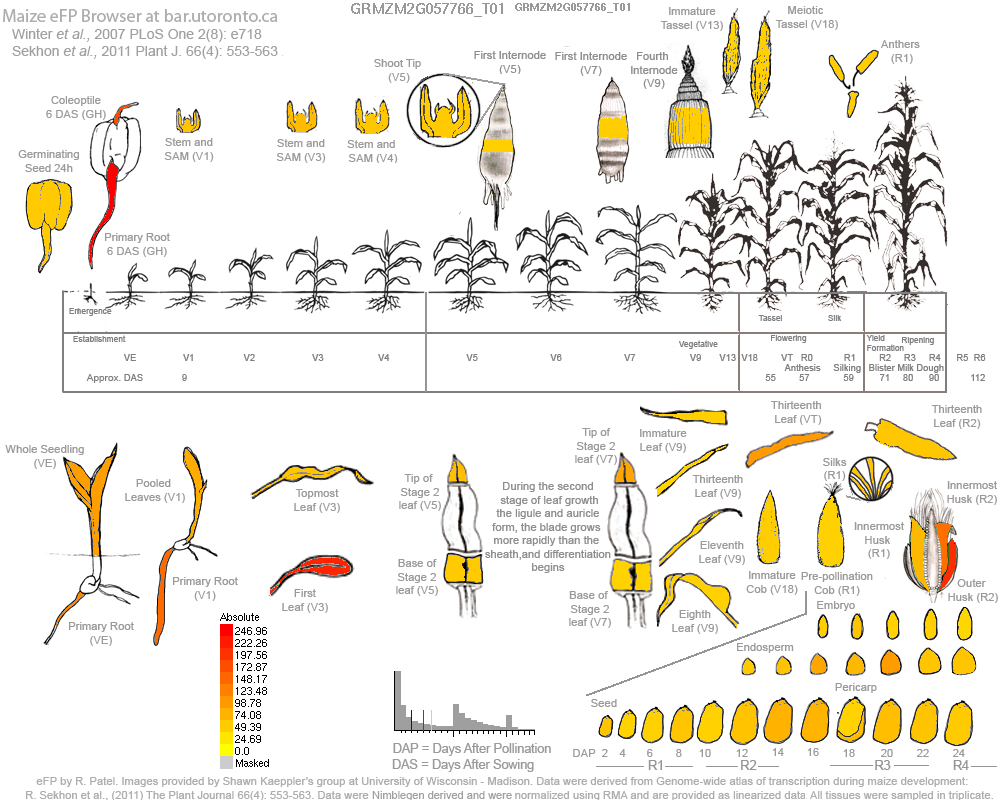


#### GRMZM2G141456^a^


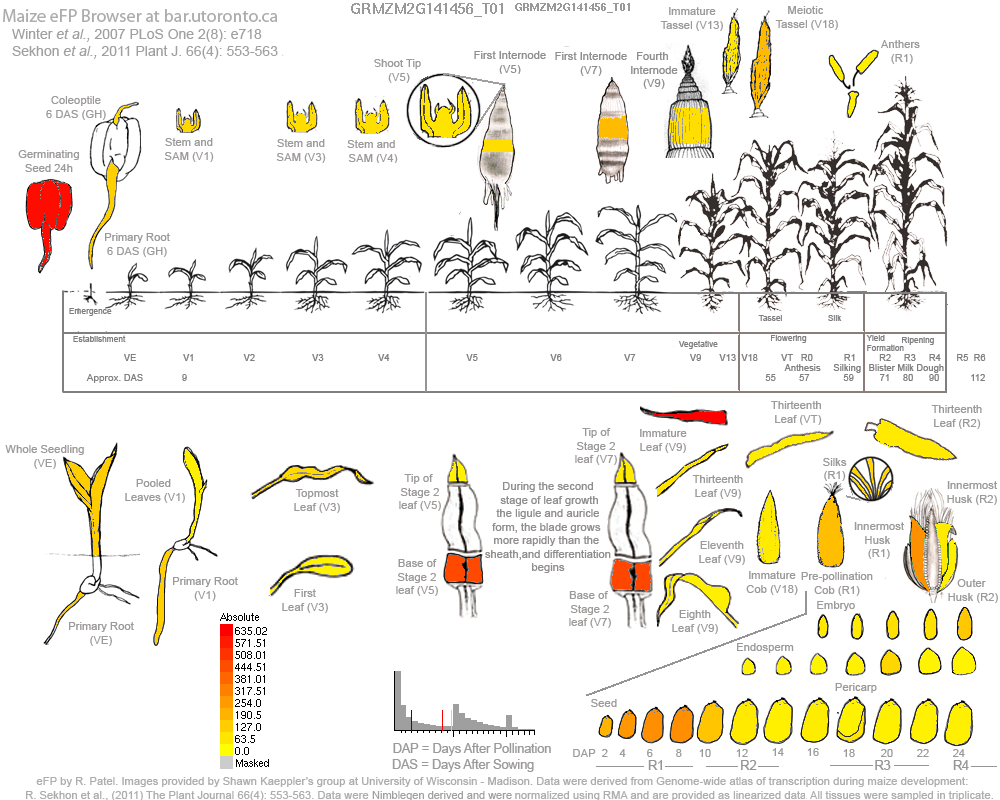


## Chromosome 6

#### GRMZM2G034598^a^


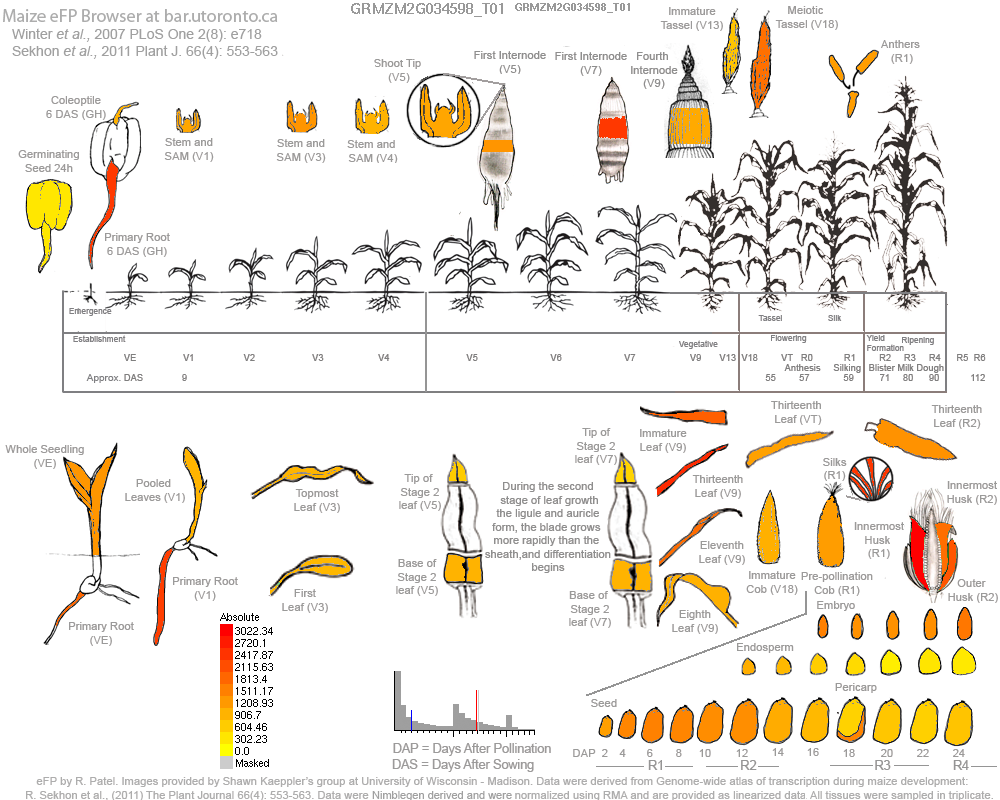


#### GRMZM2G412577^g^


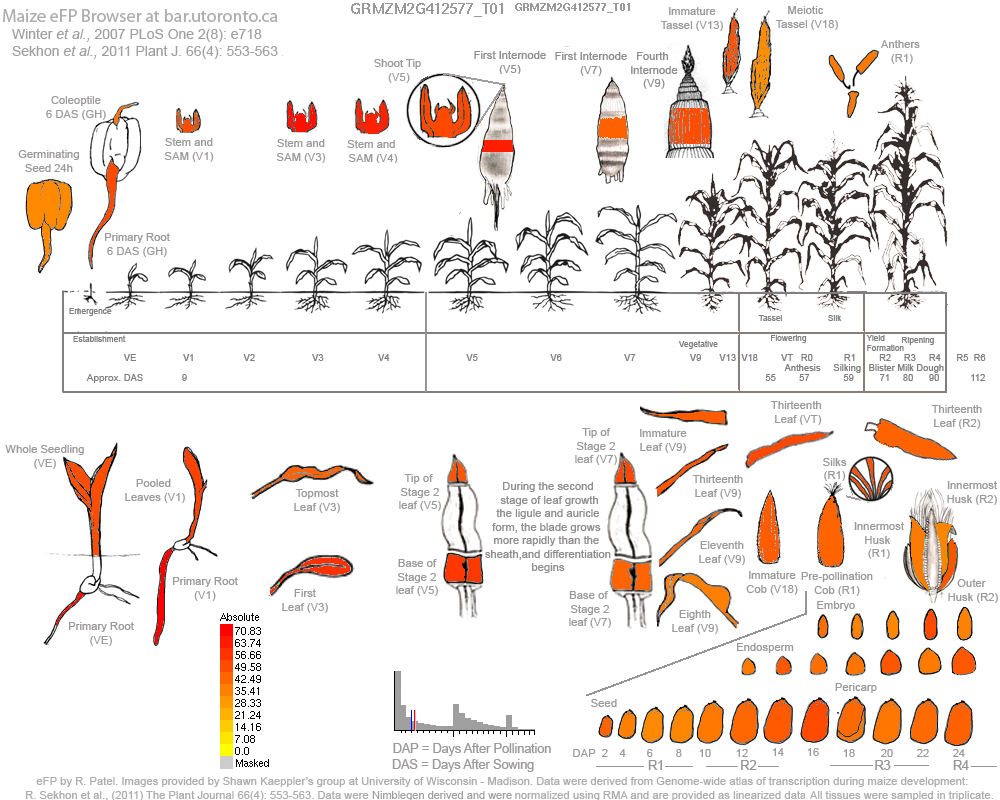


#### GRMZM2G145518^e^


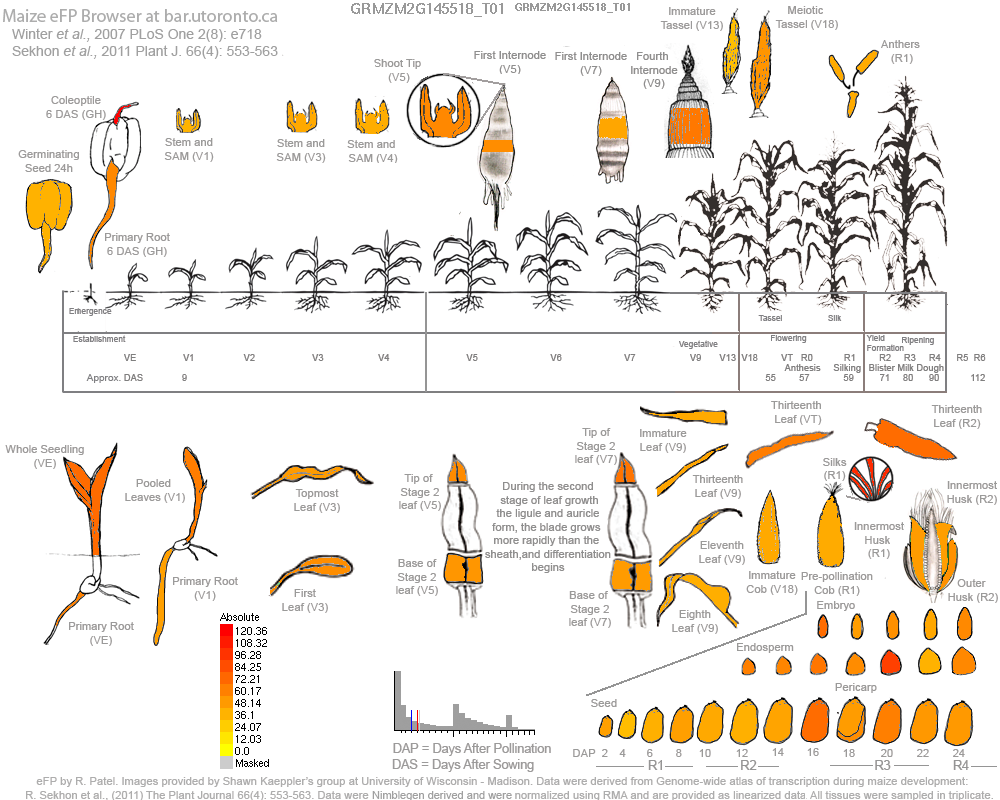


#### GRMZM2G447967^e^


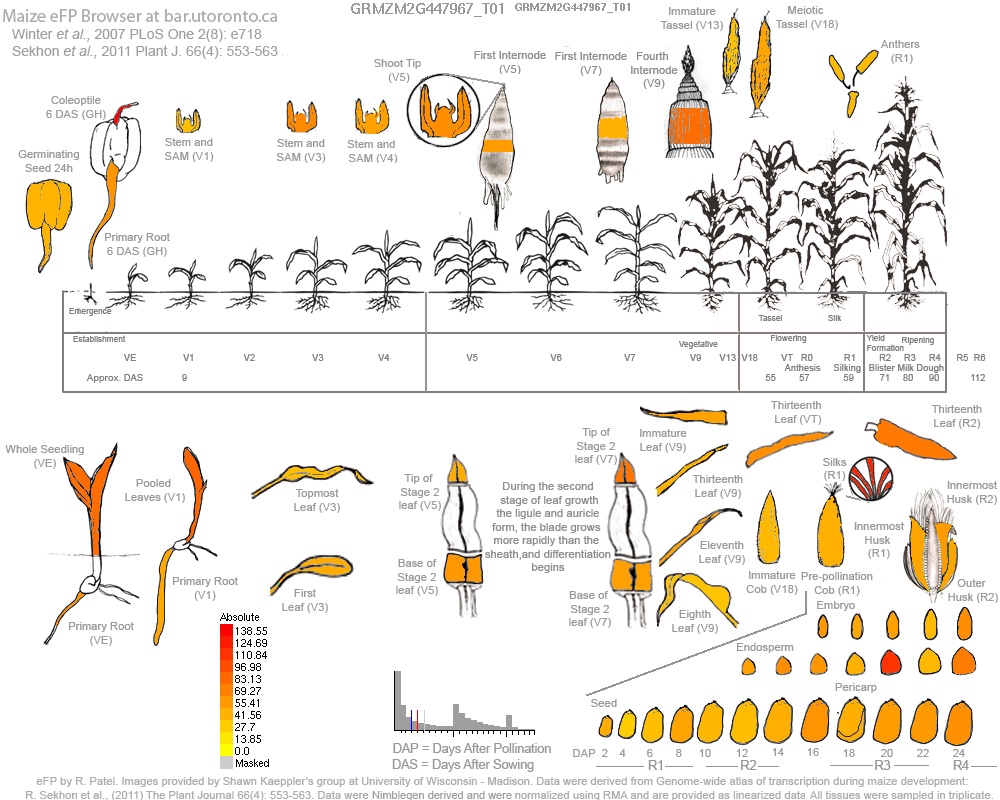


#### GRMZM2G145461^a^


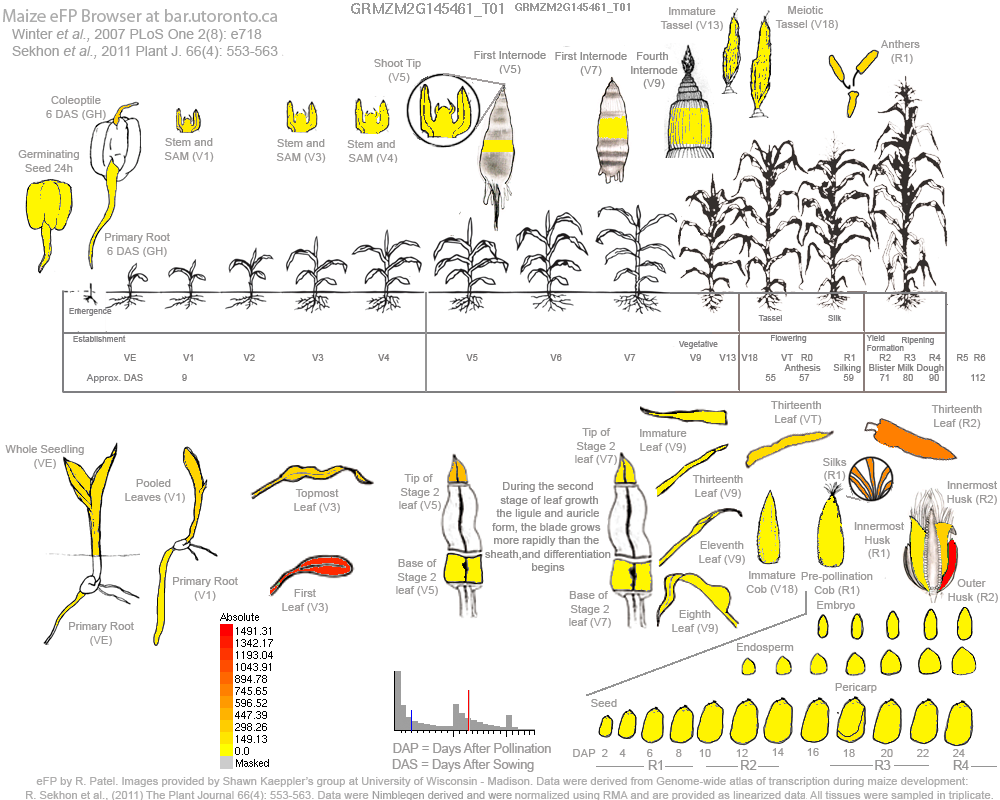


#### GRMZM2G447795^a^


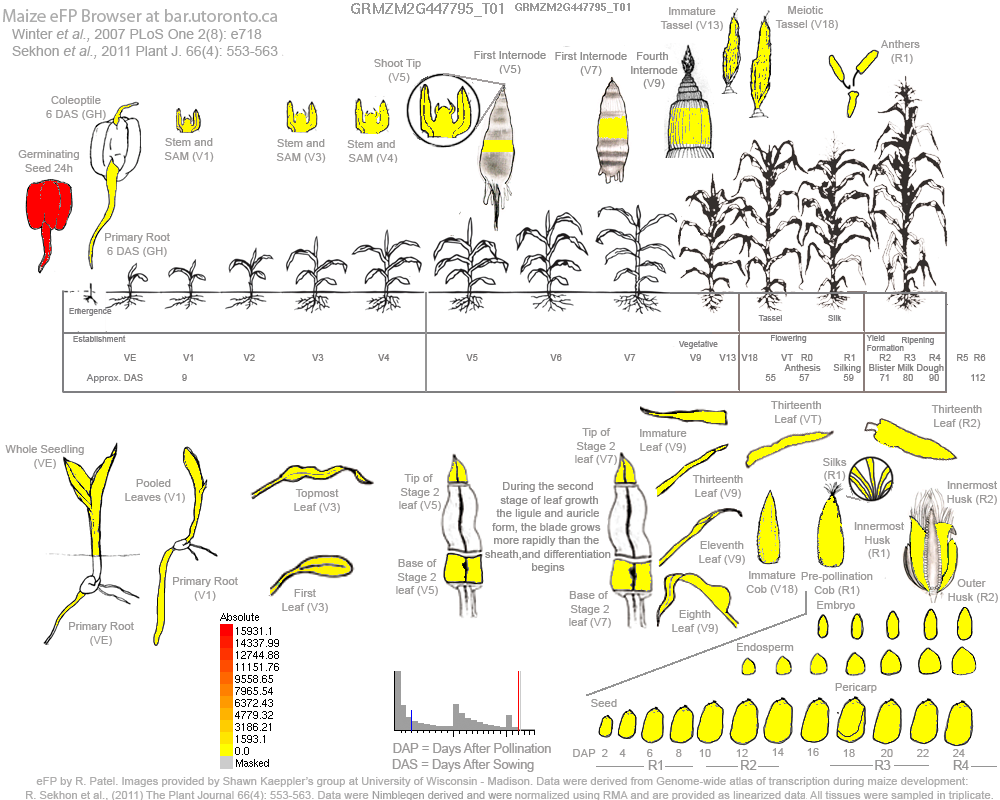


## Chromosome 7

#### GRMZM2G328171^a^


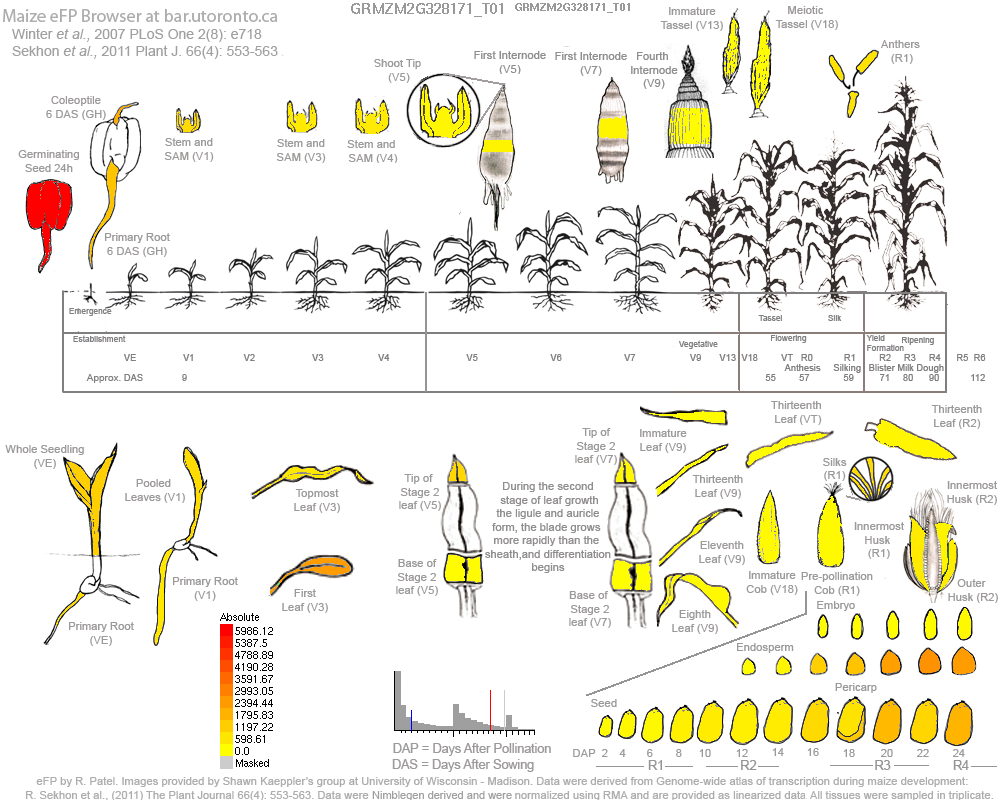


#### GRMZM2G162359^a^


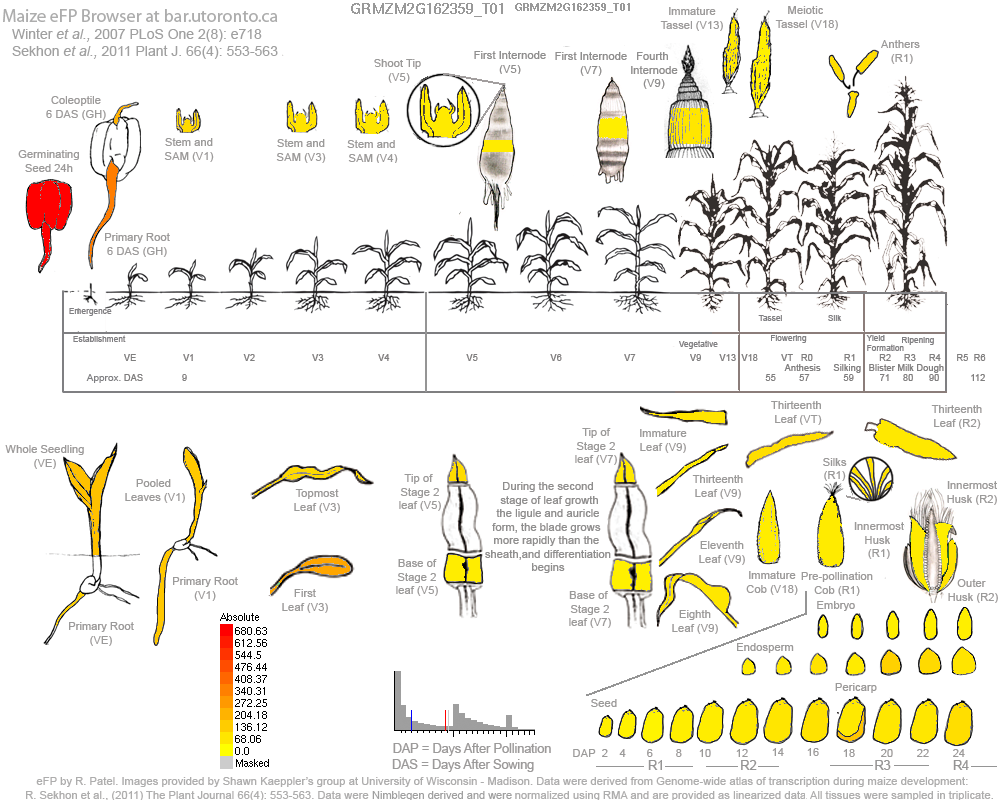


#### GRMZM2G168364^a^


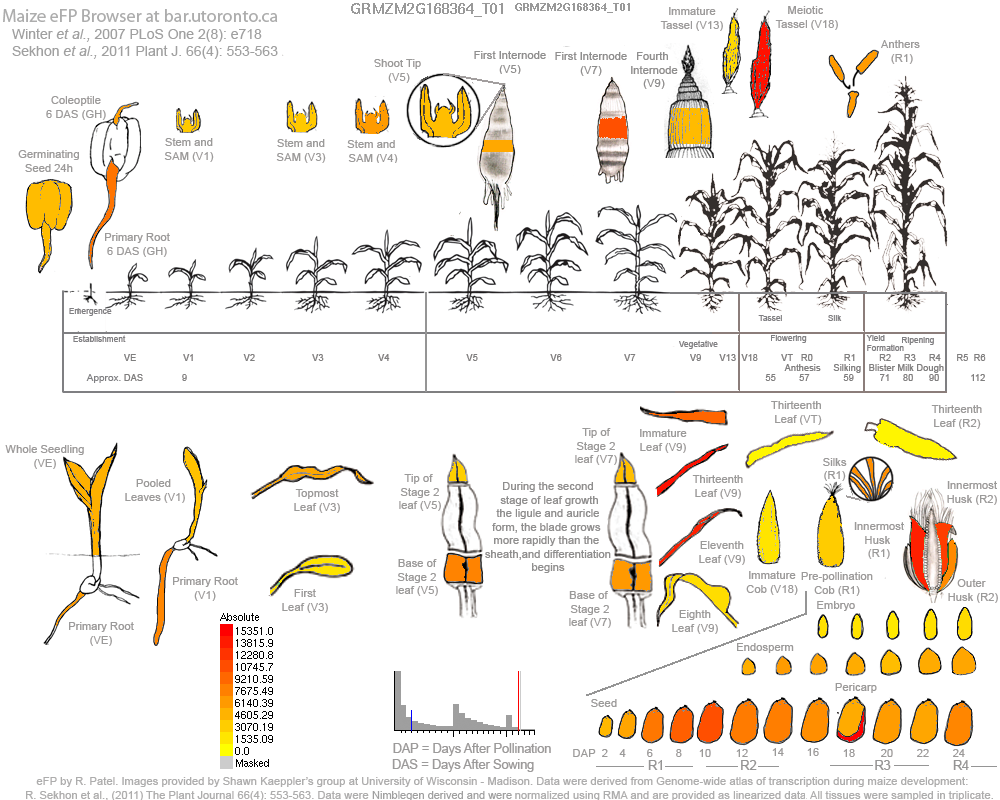


## Chromosome 8

#### GRMZM2G400497^g^


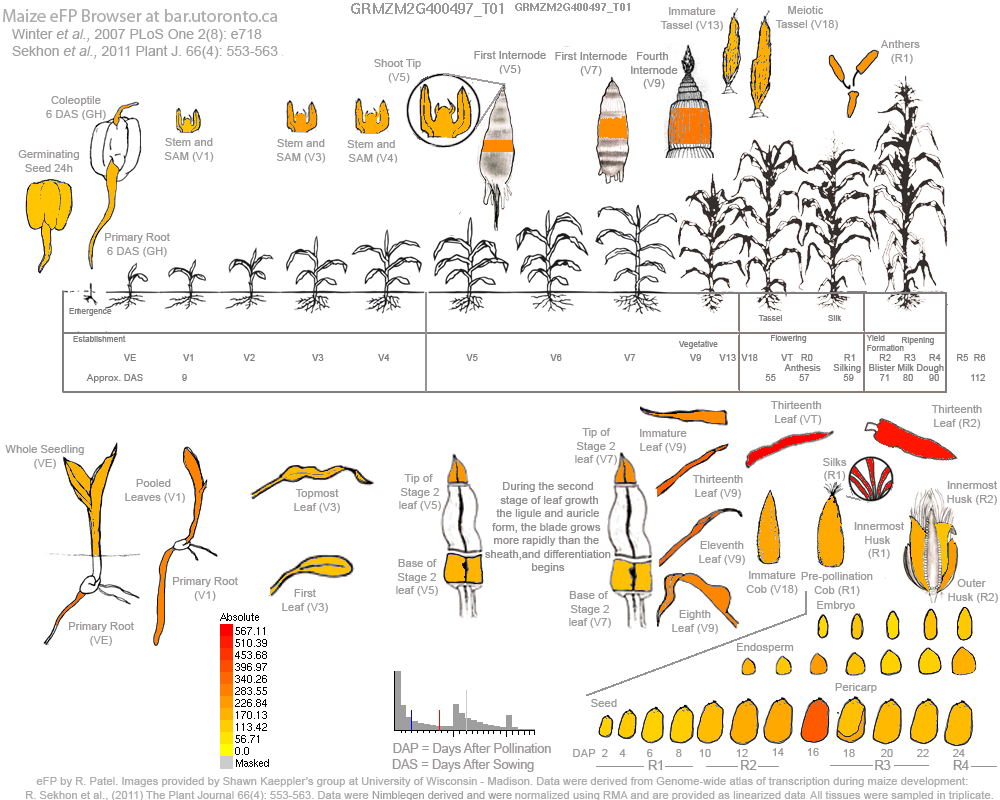


#### GRMZM2G062974^a^


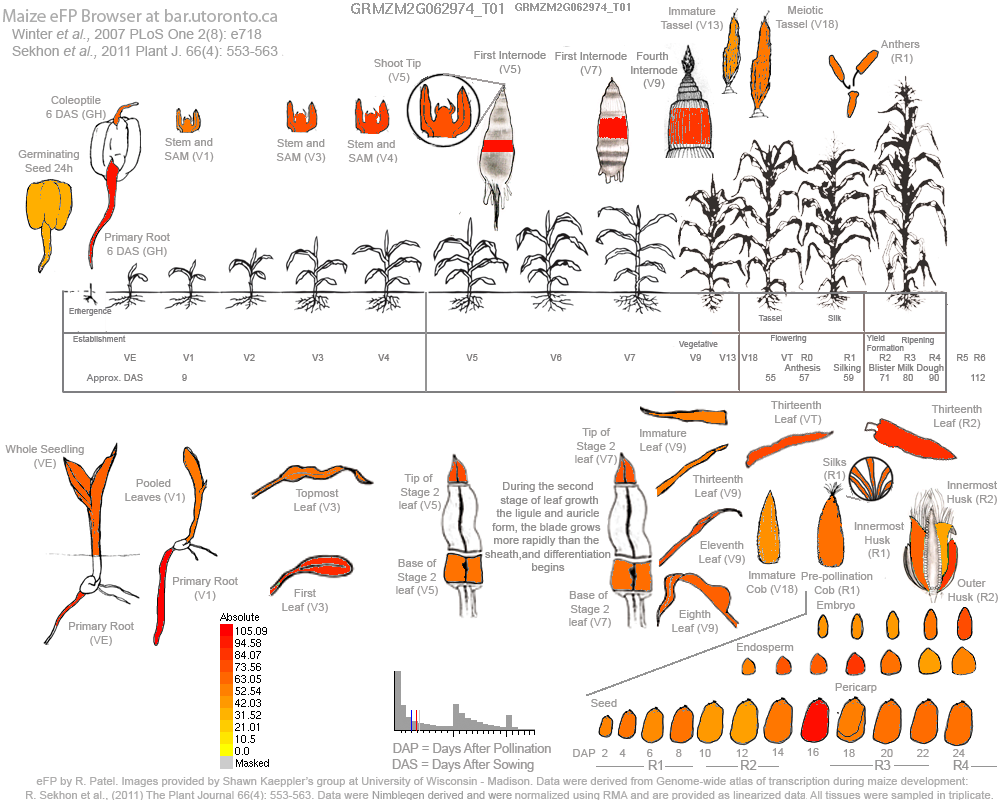


#### GRMZM2G083292^g^


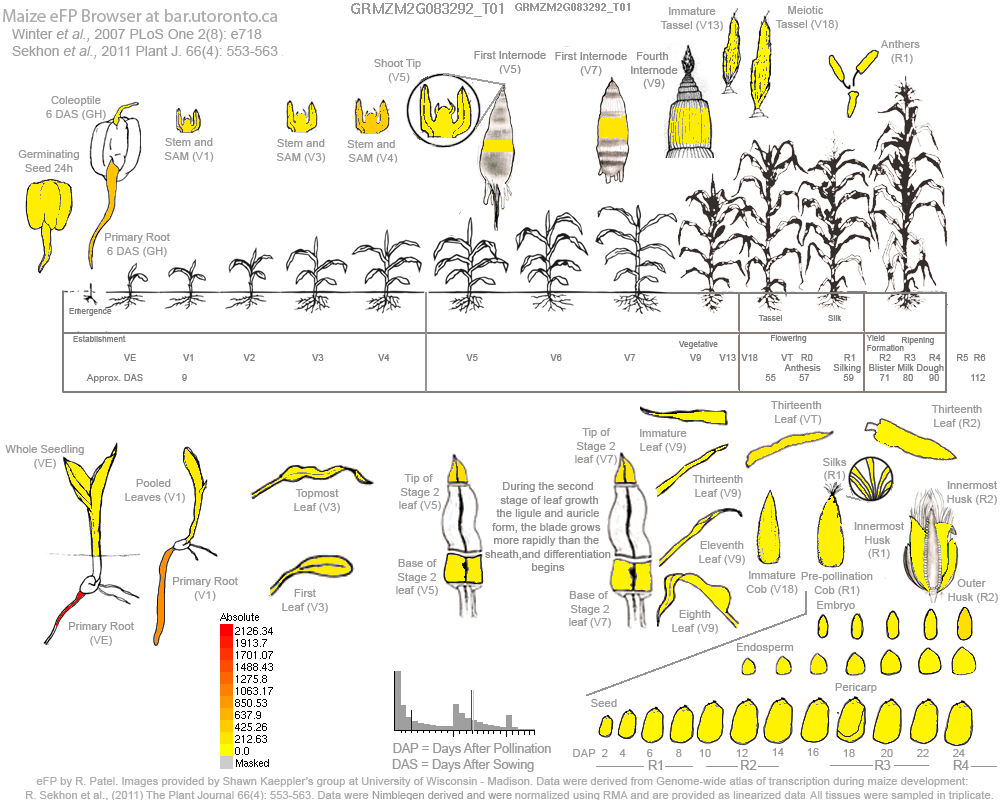


#### GRMZM2G037694^d^


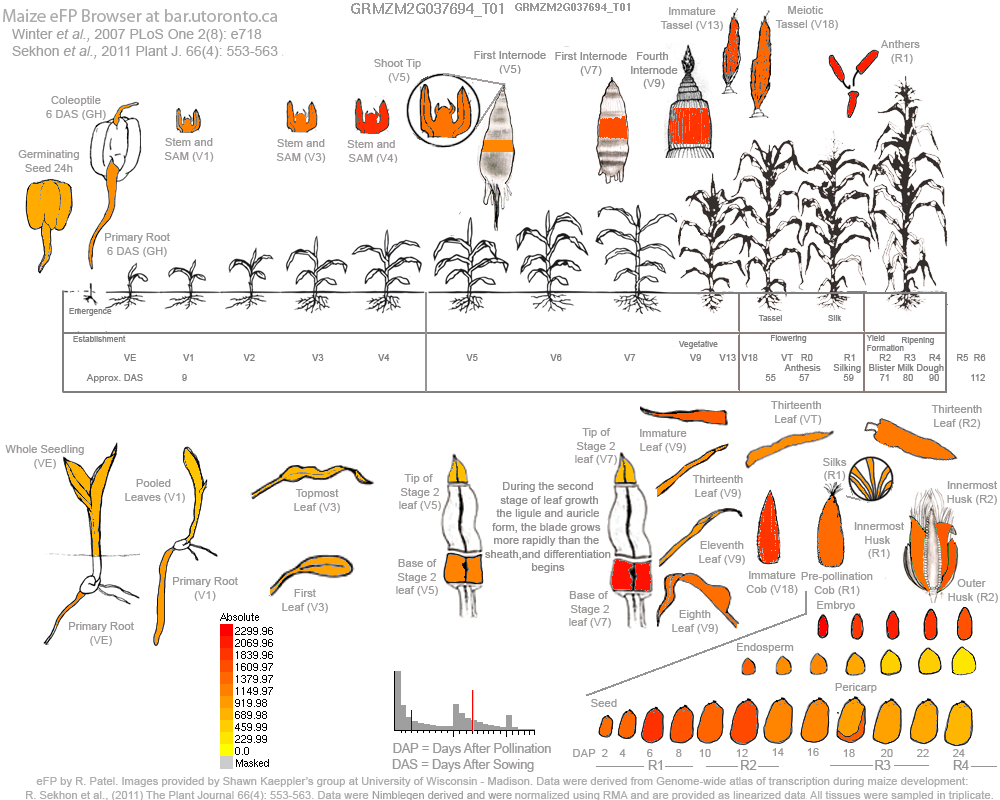


#### GRMZM2G117405^a^


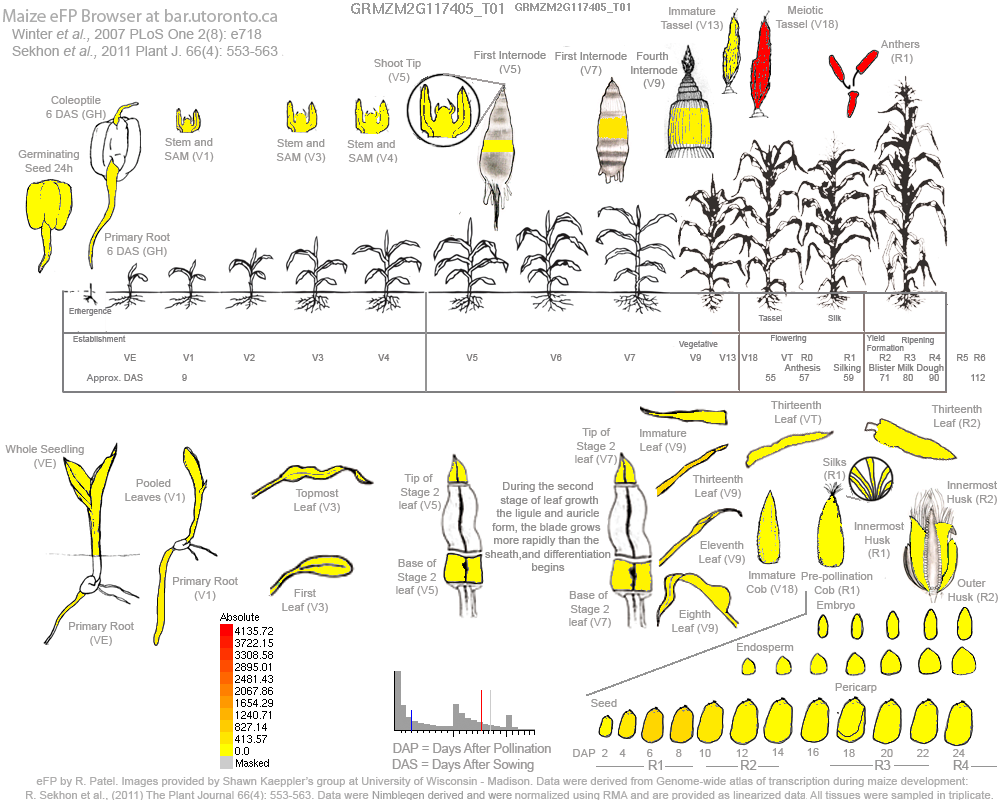


## Chromosome 10

#### GRMZM2G400999^h^

#### GRMZM2G090441^a^


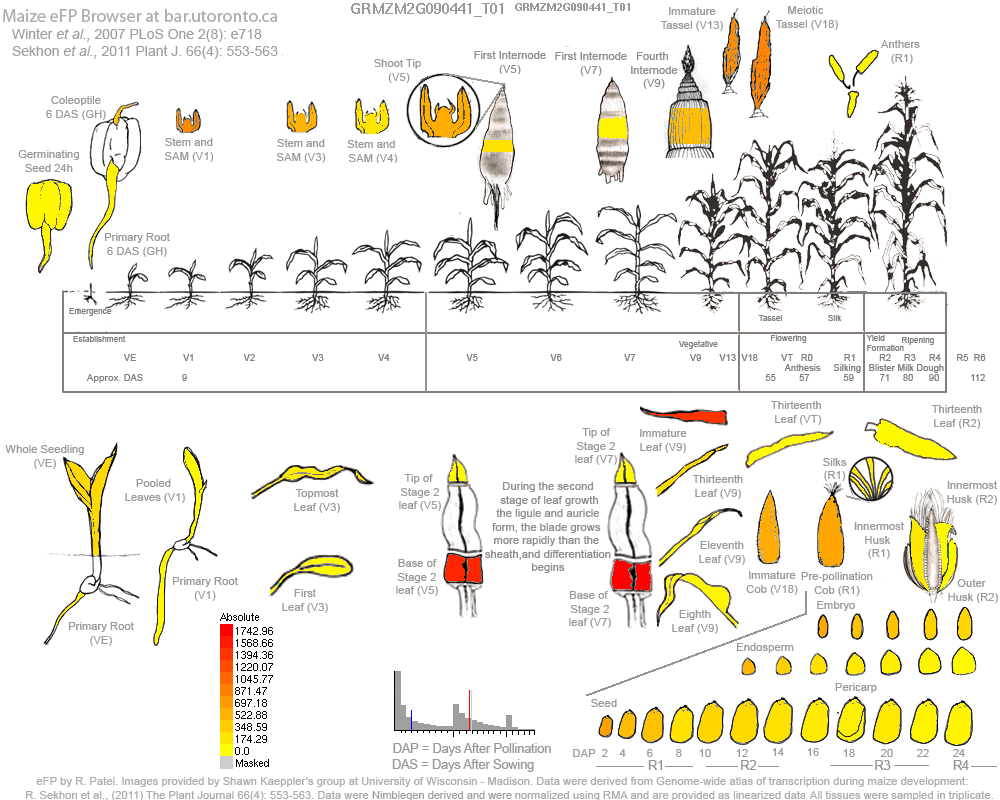


#### GRMZM2G005633^a^


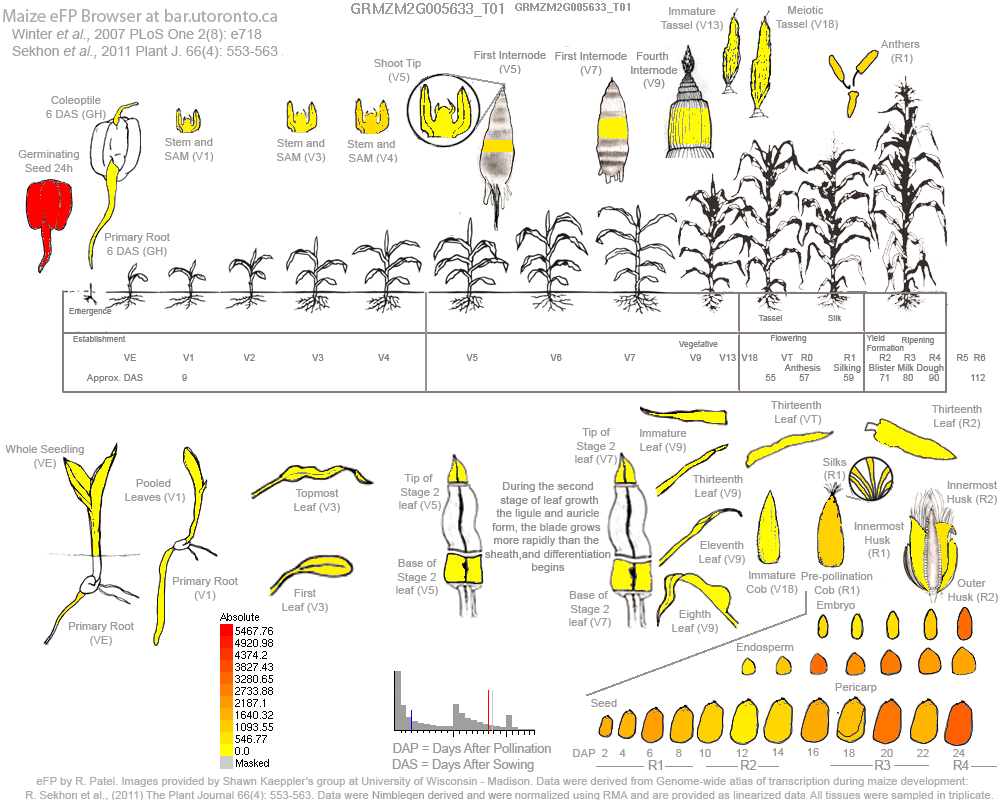

Supplement: S1 Fig — a = Similar Expression information from Qteller; b = Qteller indicates greater expression in the undifferentiated ear, appears to be constitutively expressed; c = Expression levels are very low from the Qteller output; d = Very low expression in Qteller; e = Qteller indicates high expression levels in the ear, silks, tassel, and/ or roots; f = Conflicting results, appears to be constitutive in Sekhon, but more expressed in the seeds in Qteller. Expression levels are low for both; g = Not studied in Qteller; h = Not studied by Sekhon et al. (DOCX) [file pone.0126185.s001.docx]
