# Supplementary material for: Characterization of the Maize Chitinase Genes and Their Effect on Aspergillus flavus and Aflatoxin Accumulation Resistance
Source: PLoS One. 2015 Jun 19;10(6):e0126185. doi: 10.1371/journal.pone.0126185 (PMC4475072; doi:10.1371/journal.pone.0126185)
Supplement: S2 Fig — The mapping populations, and previous references for them, are: MpT = Mp715 x T173 (Warburton et al., 2011); MpB = Mp313E x B73 (Brooks et al., 2005); MpVa = Mp313E x Va35 (Willcox et al., 2013); MpNC = Mp717 x NC300 (Warburton et al., 2009). New markers used to test the chitinase genes are shown with a red circle. (DOCX) [file pone.0126185.s002.docx]

Supplemental Figure 2. Linkage maps of four QTL mapping populations showing the QTL reported previously and new QTL attributed to the chitinase genes characterized in this study. The mapping populations, and previous references for them, are: MpT = Mp715 x T173 (Warburton et al., 2011); MpB = Mp313E x B73 (Brooks et al., 2005); MpVa = Mp313E x Va35 (Willcox et al., 2013); MpNC = Mp717 x NC300 (Warburton et al., 2009). New markers used to test the chitinase genes are shown with a red circle.


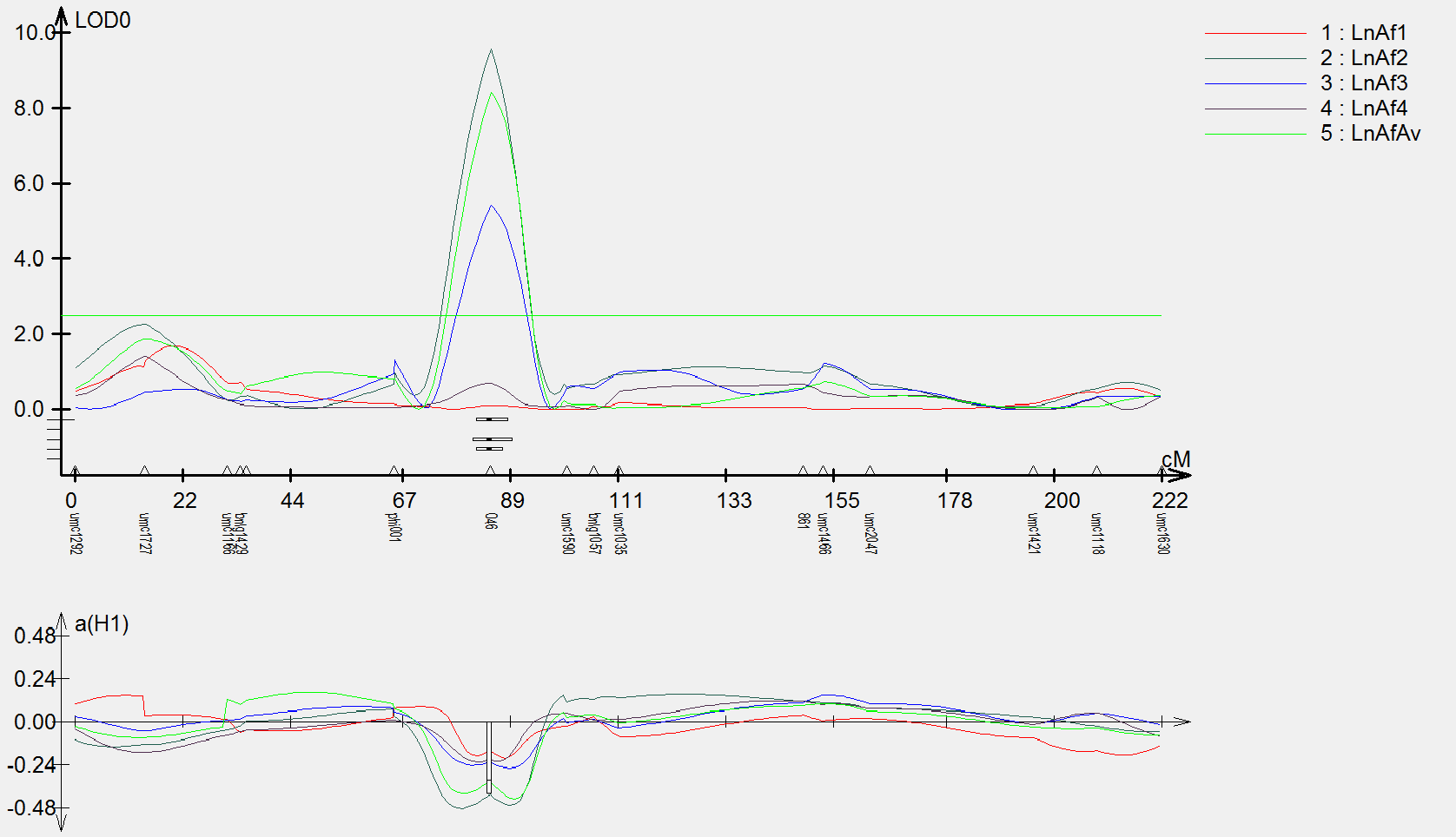

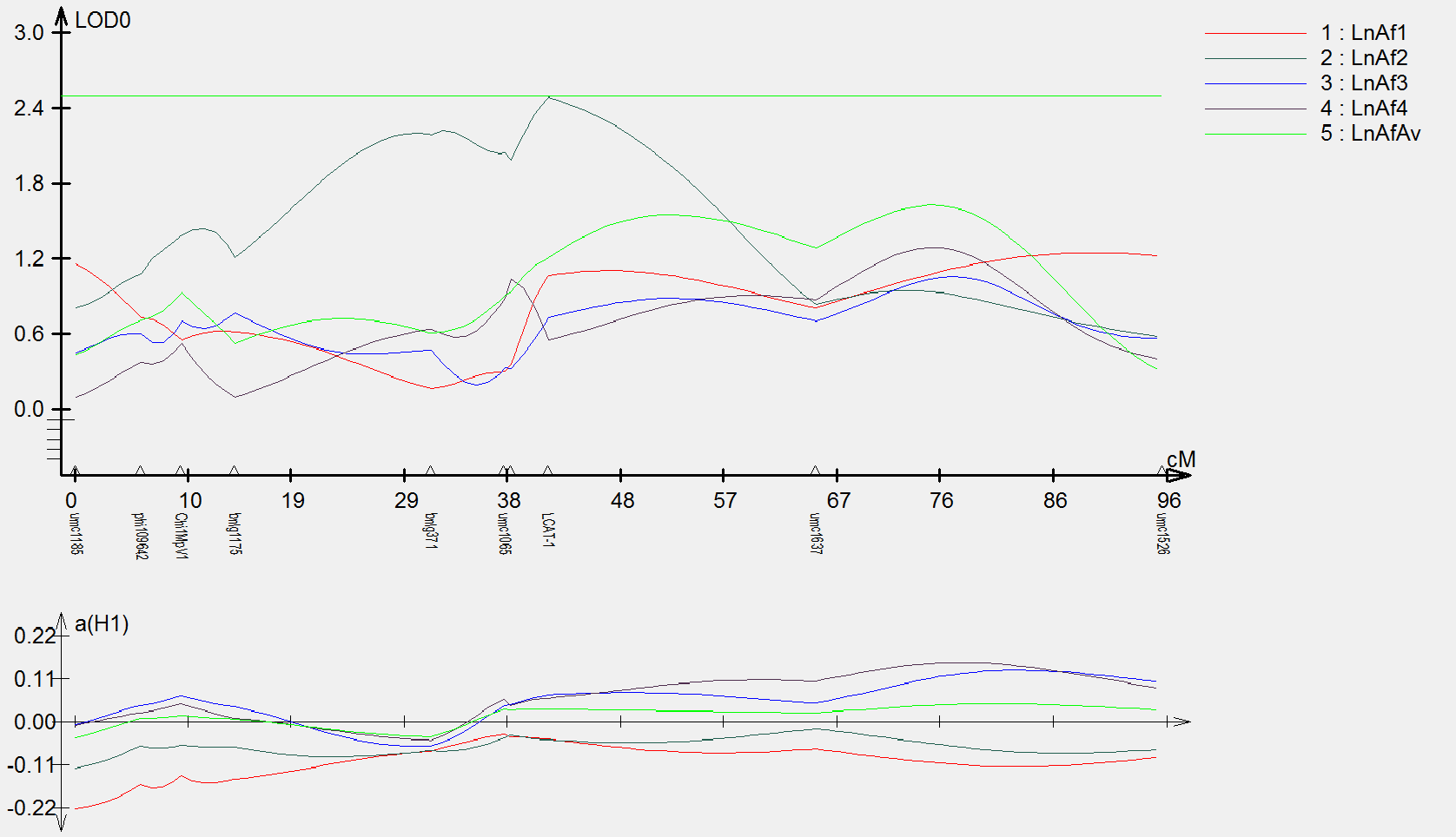

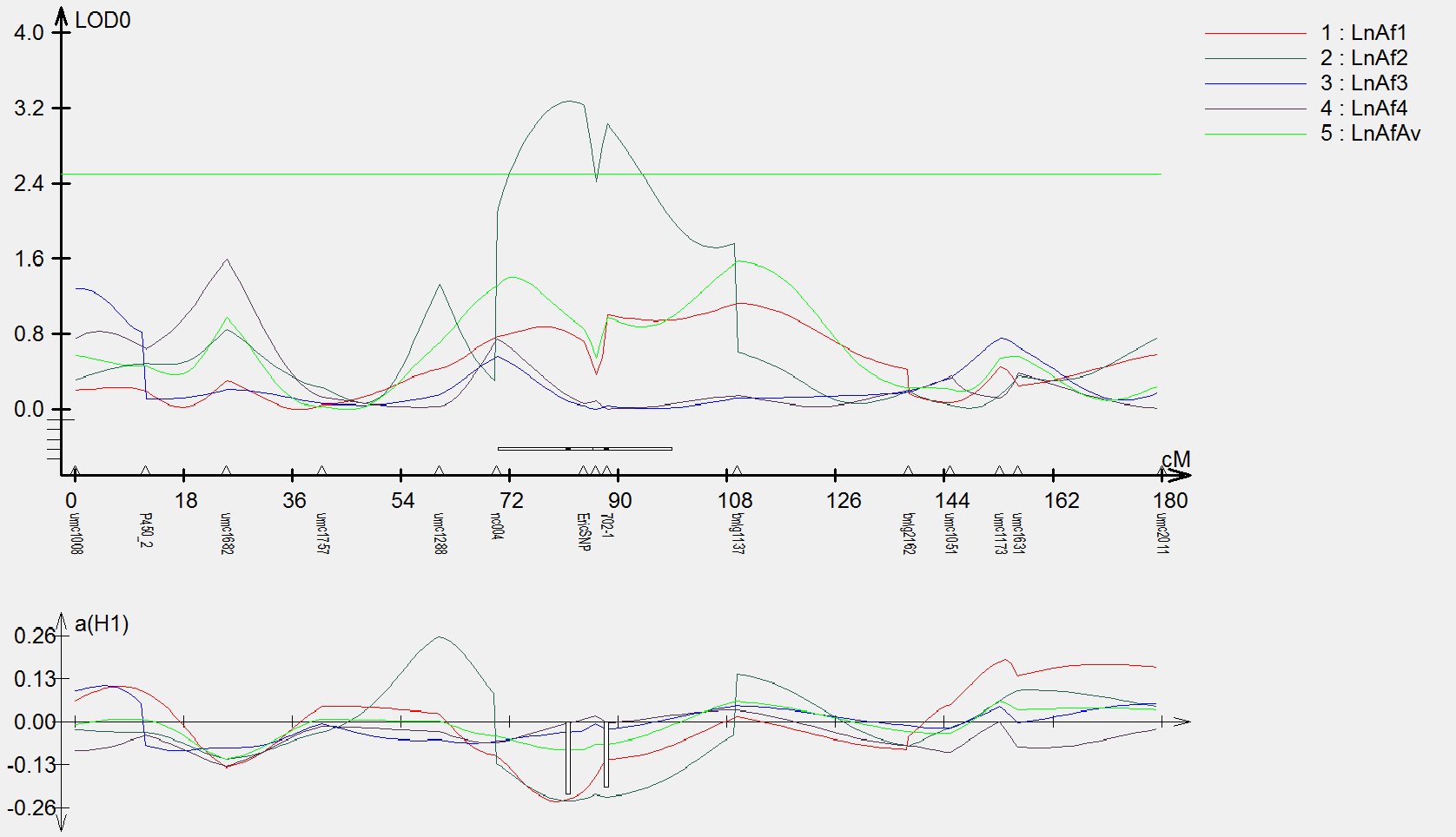

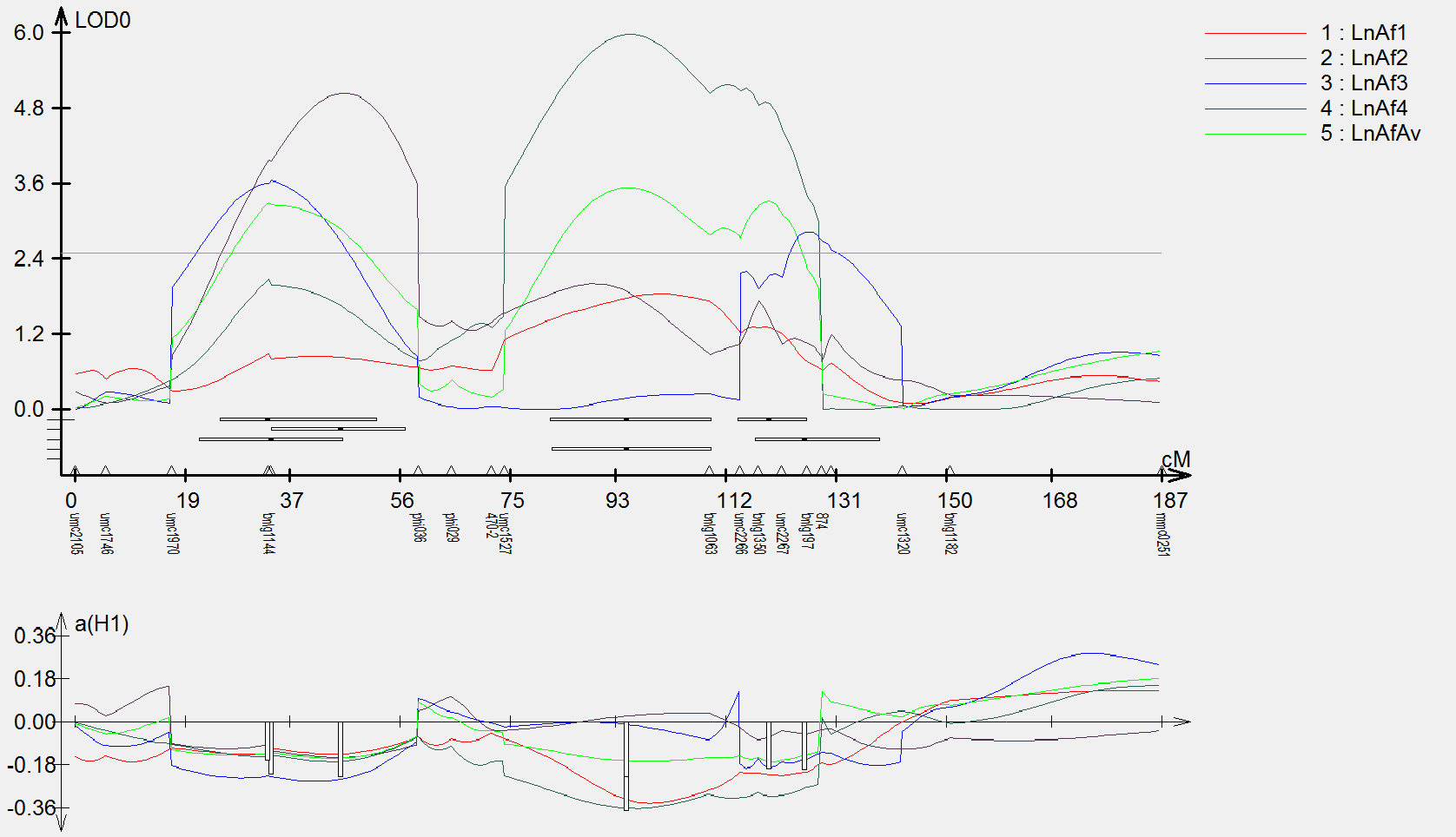
MpT mapping population

Chromosome 1 Chromosome 2


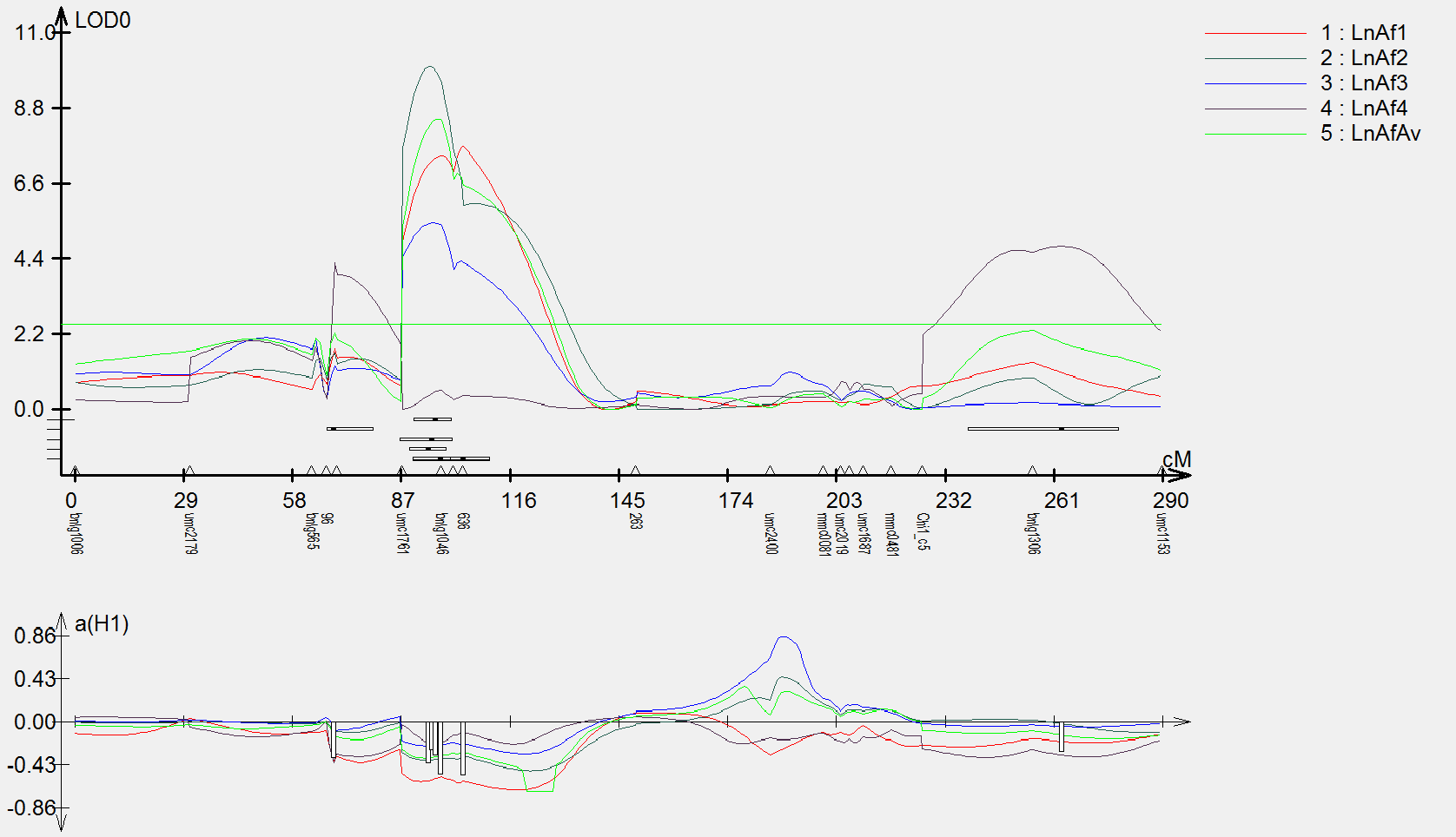

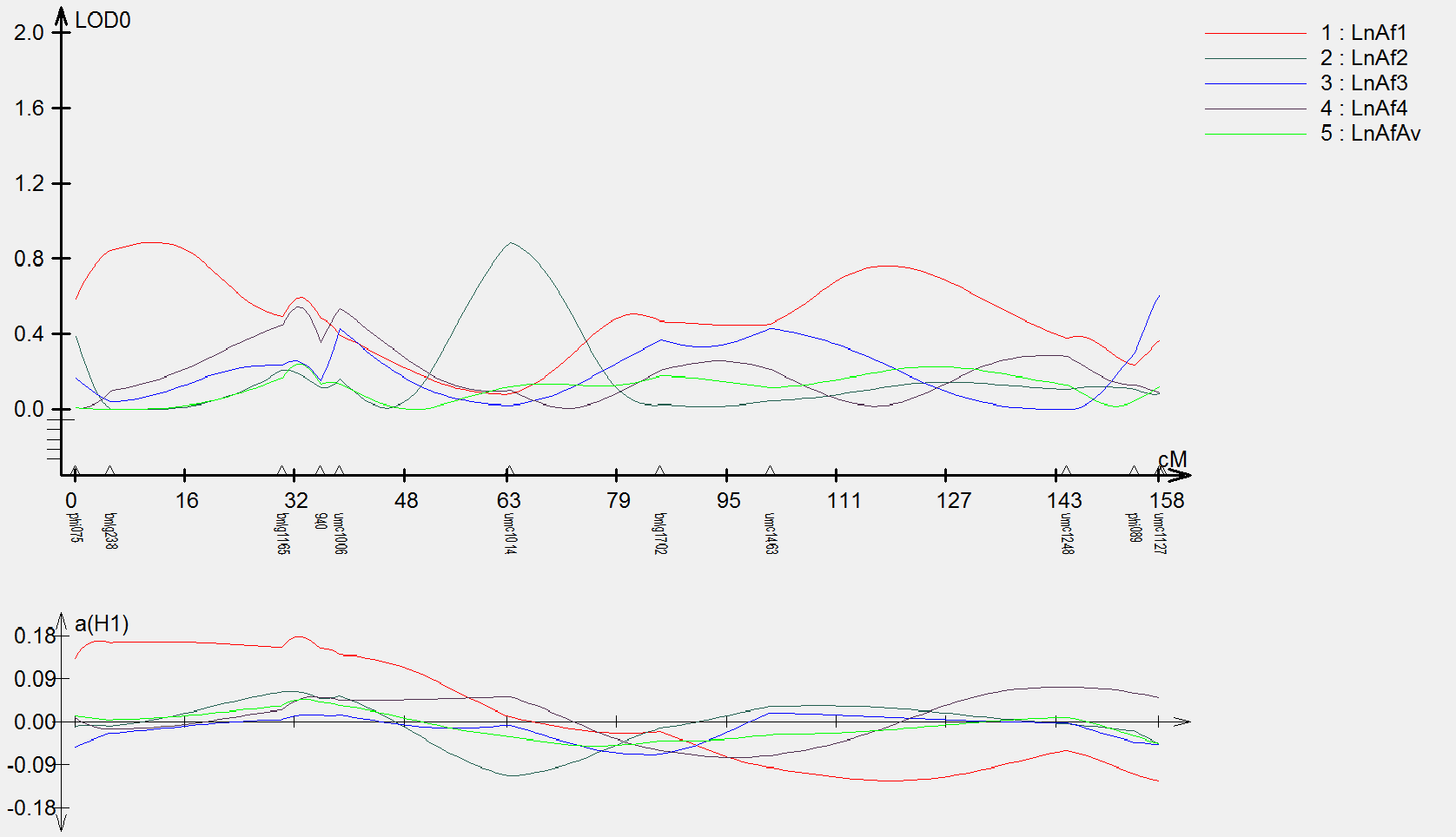
Chromosome 3 Chromosome 4


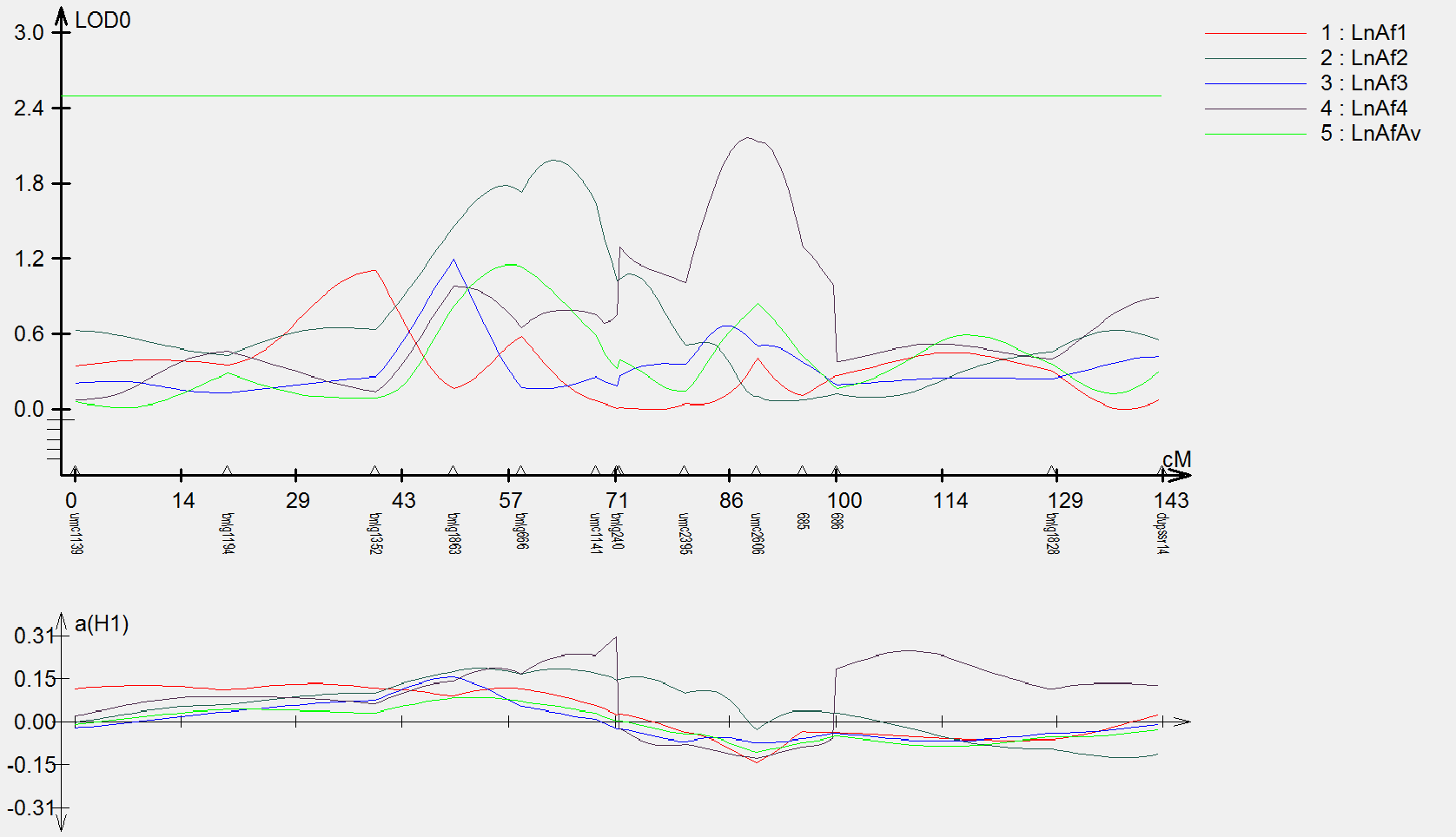

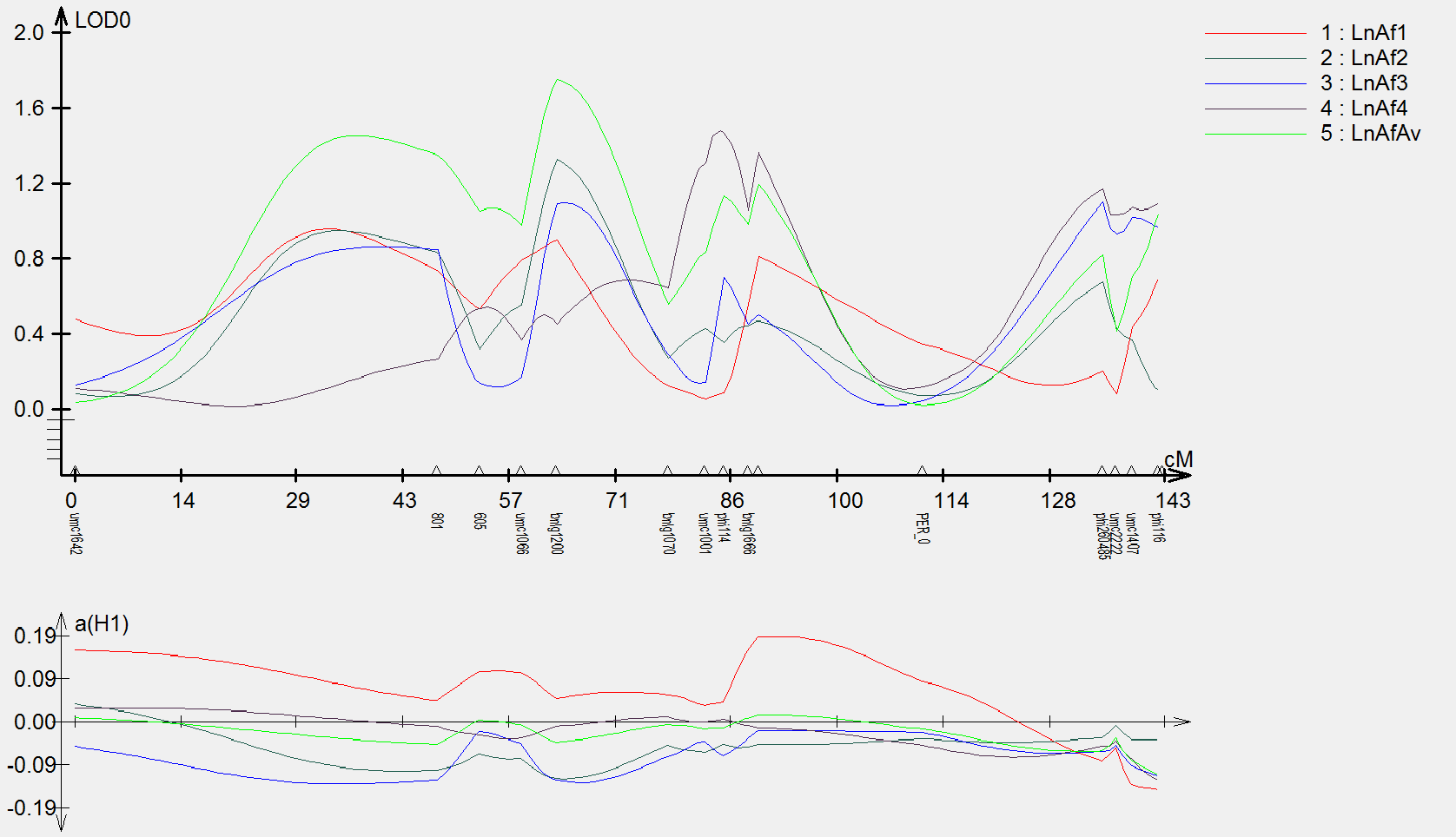
Chromosome 5 Chromosome 6

Chromosome 7 Chromosome 8


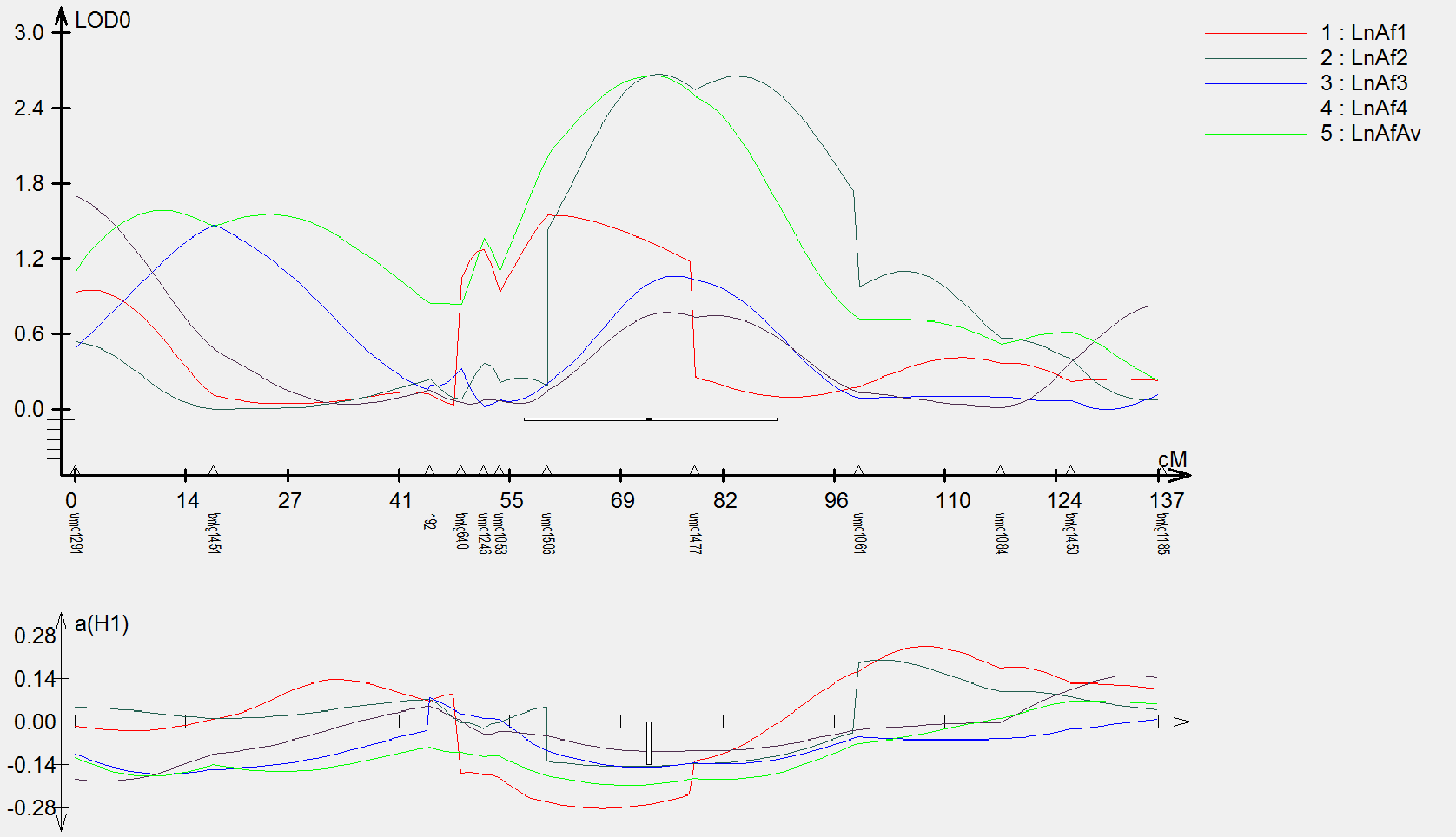

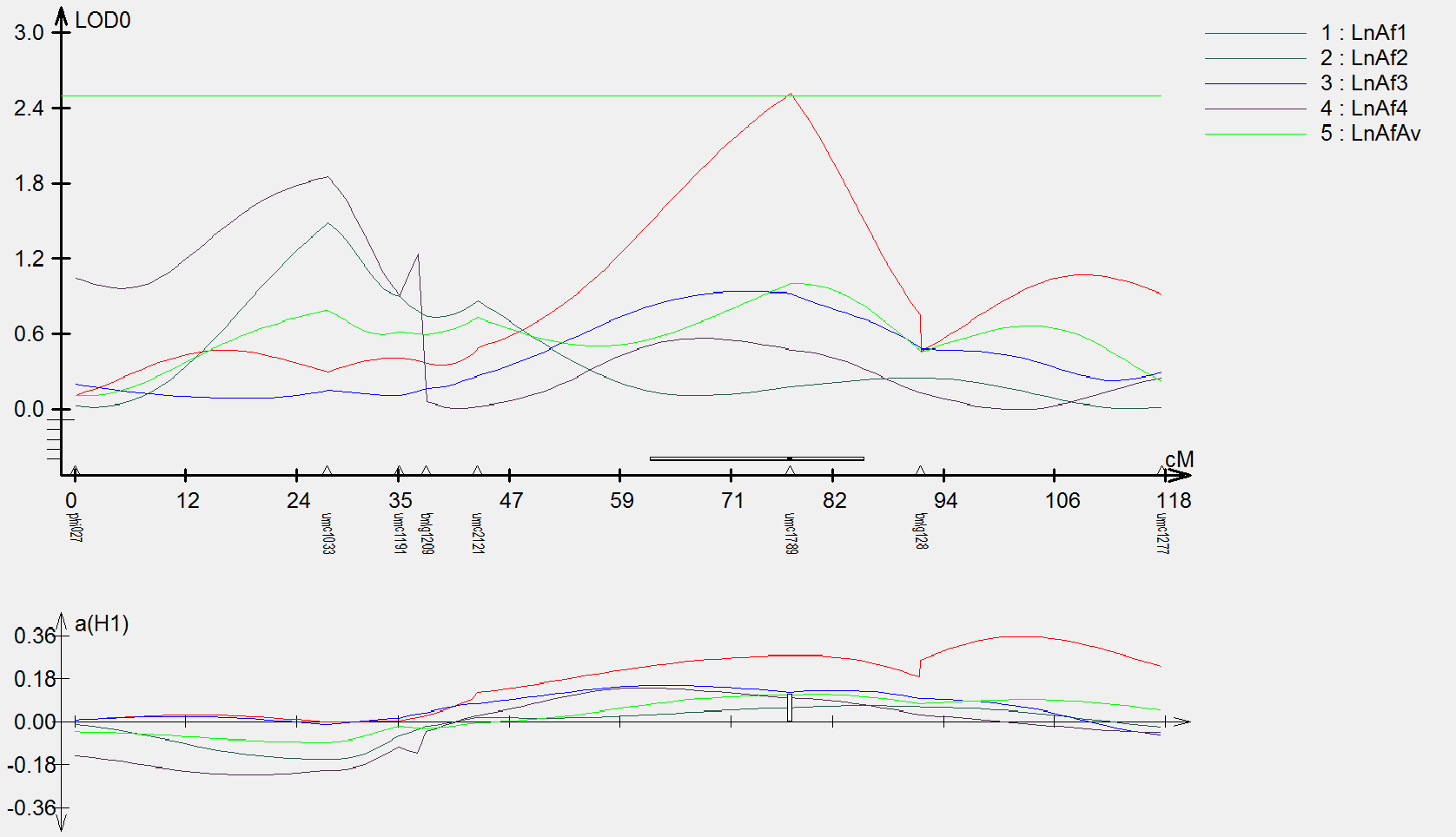
Chromosome 9 Chromosome 10


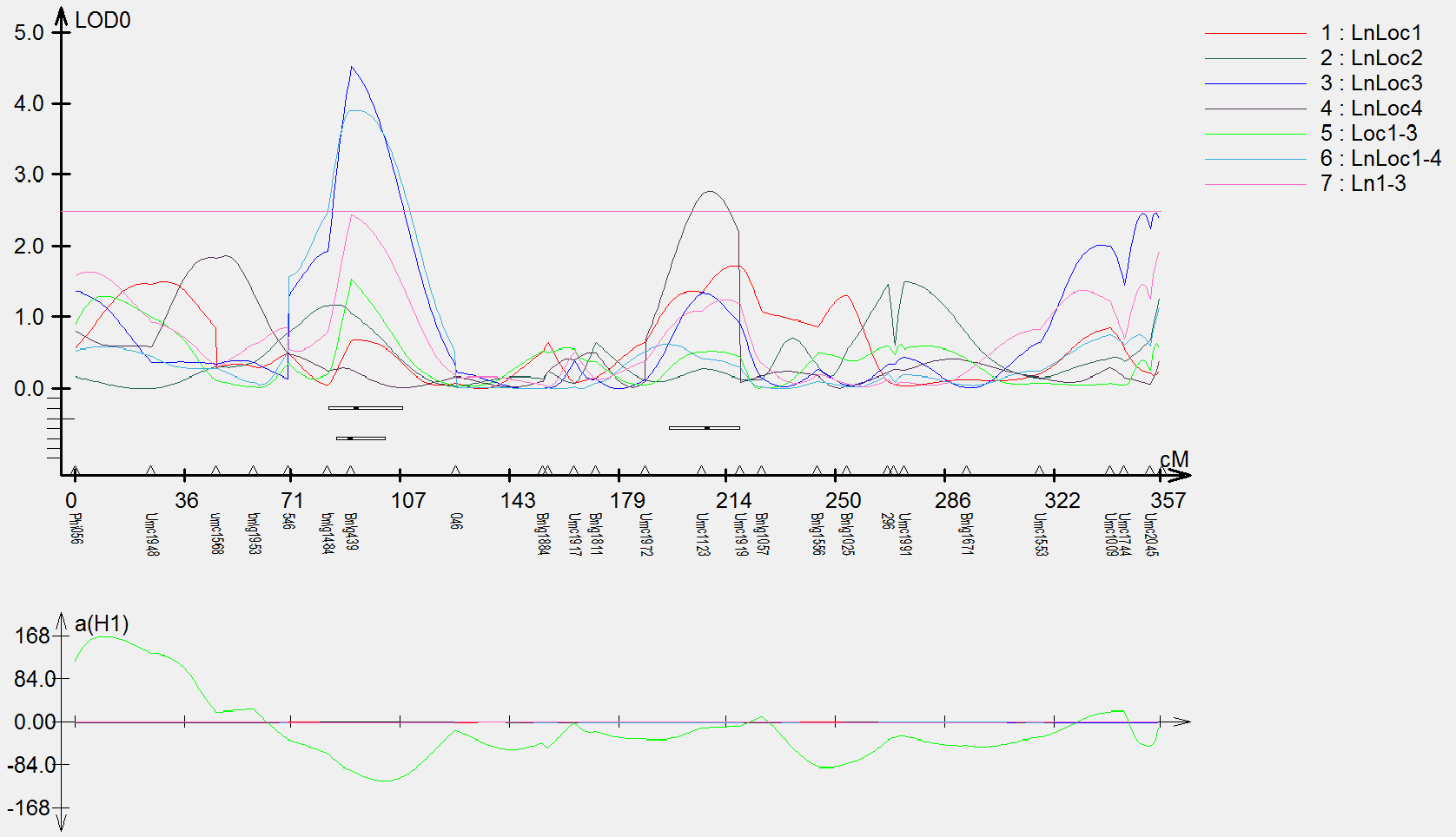

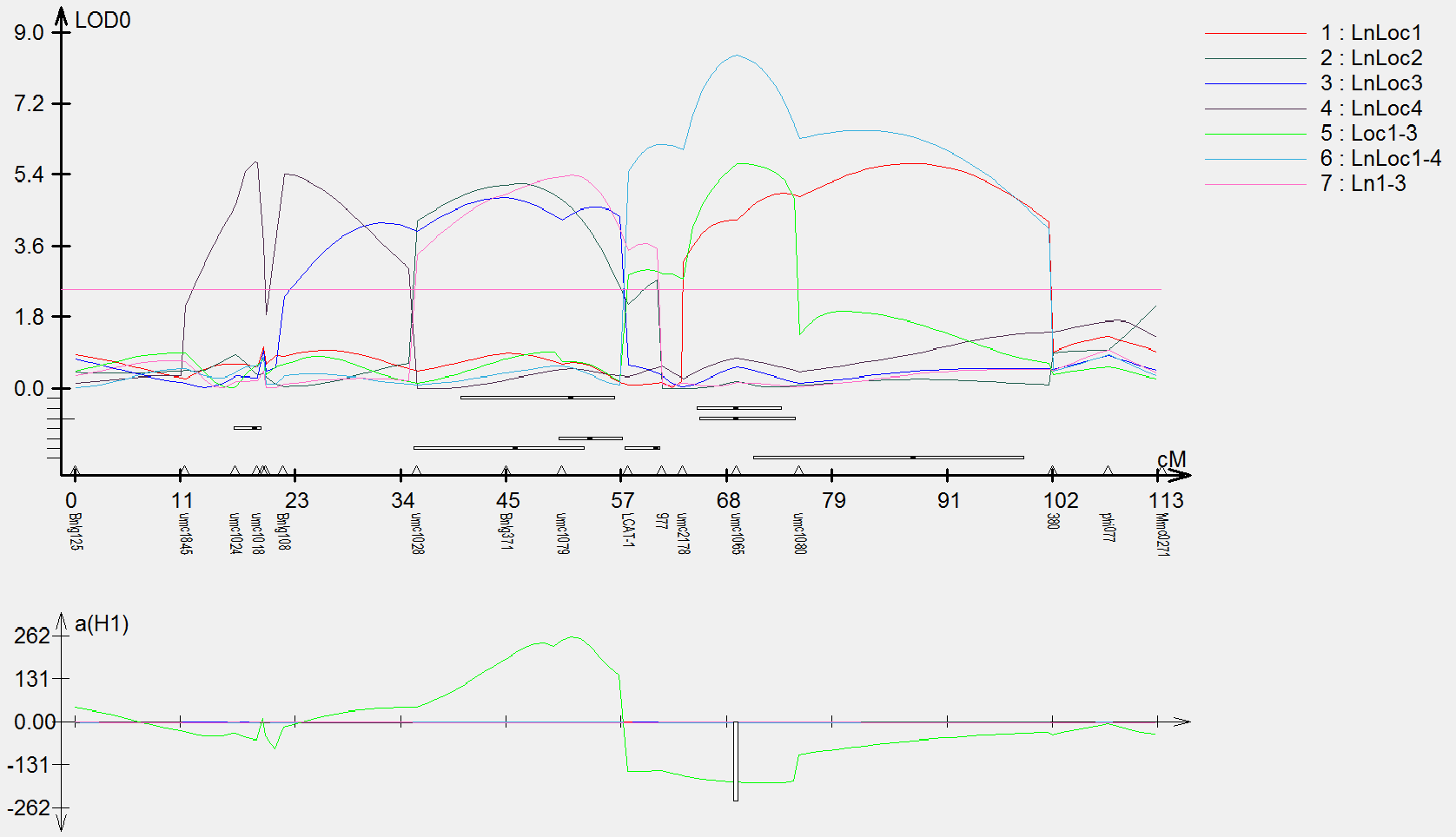
MpB mapping population


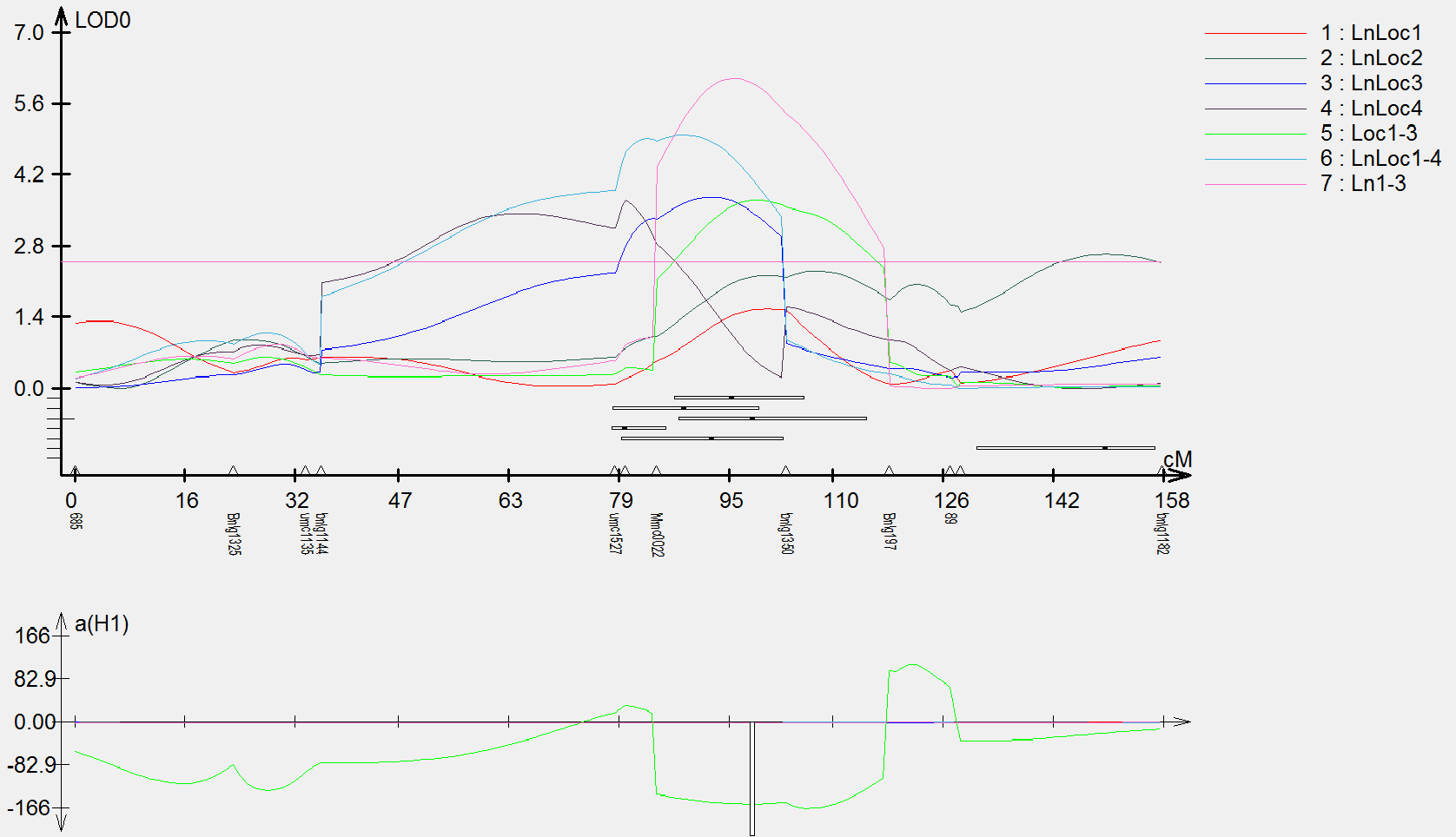

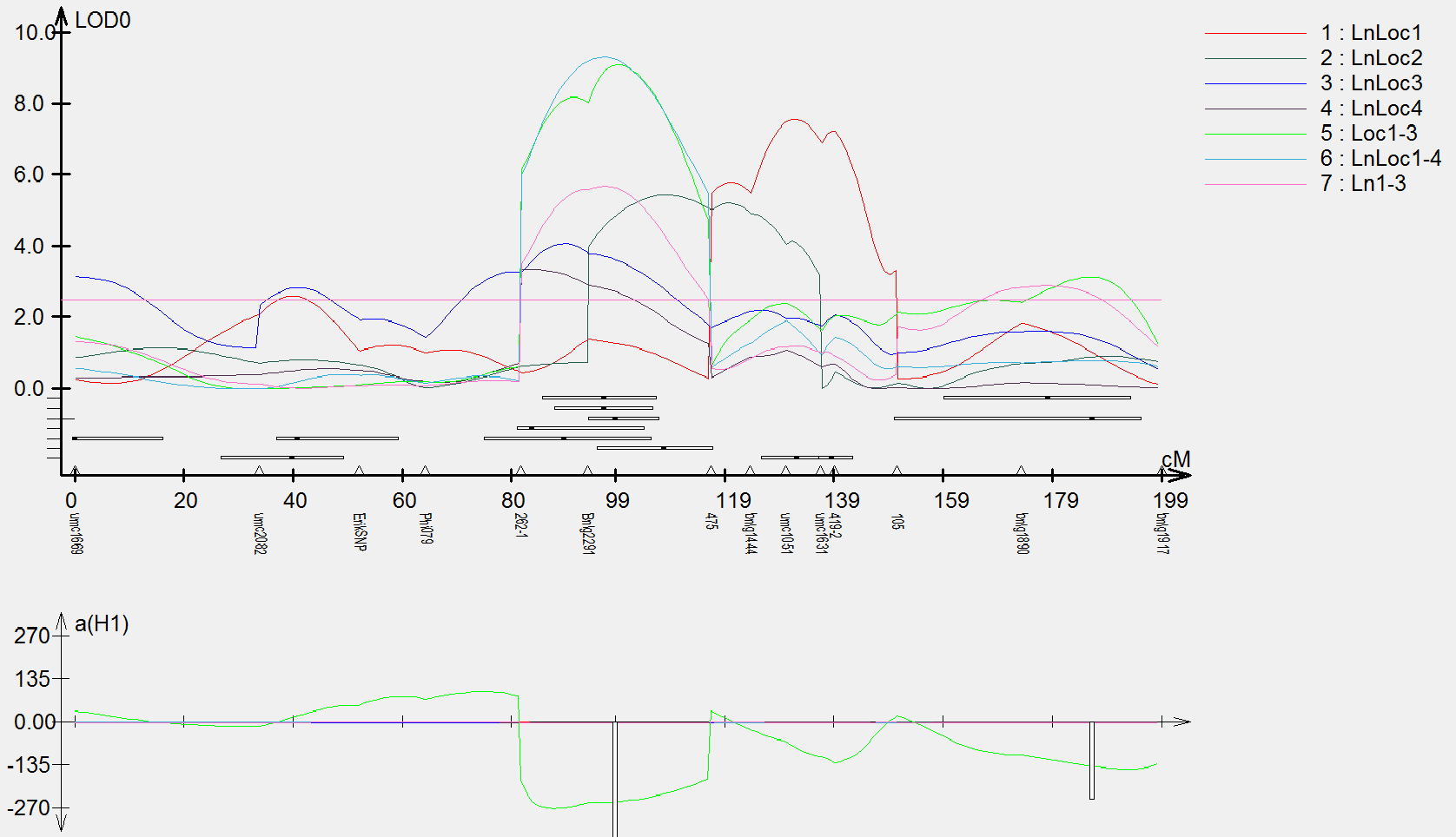
Chromosome 1 Chromosome 2


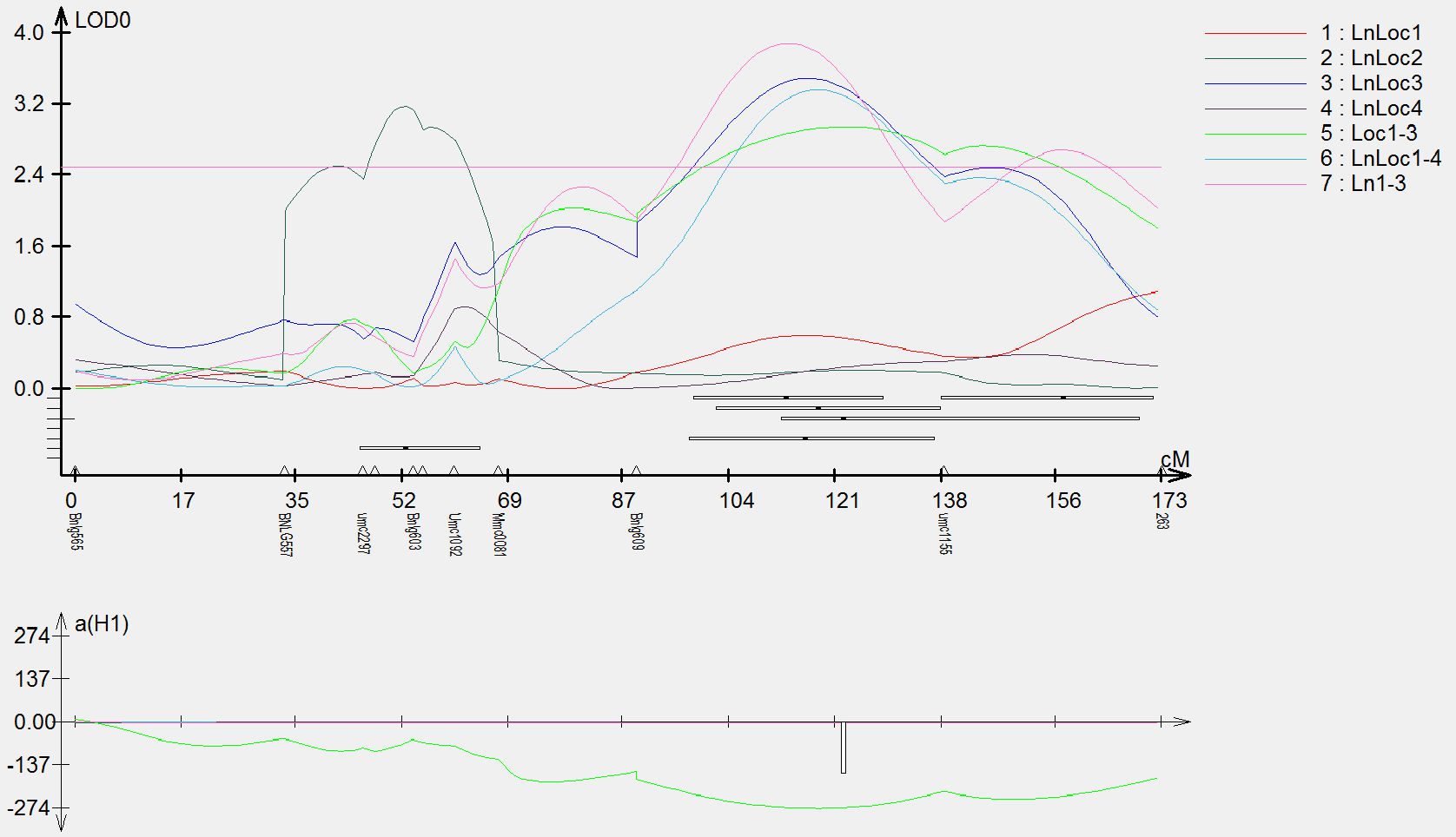

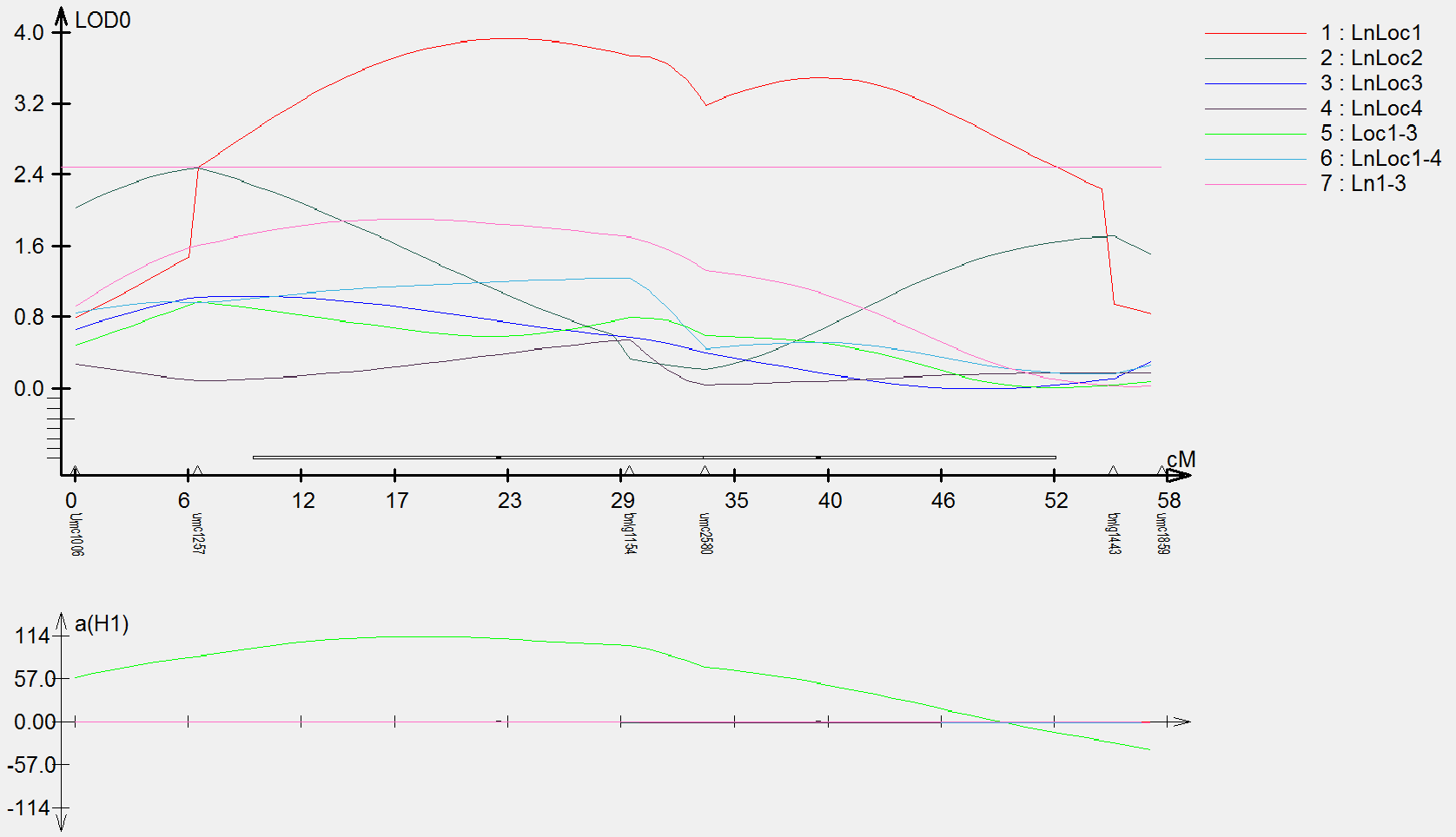
Chromosome 3 Chromosome 4

Chromosome 5 Chromosome 6


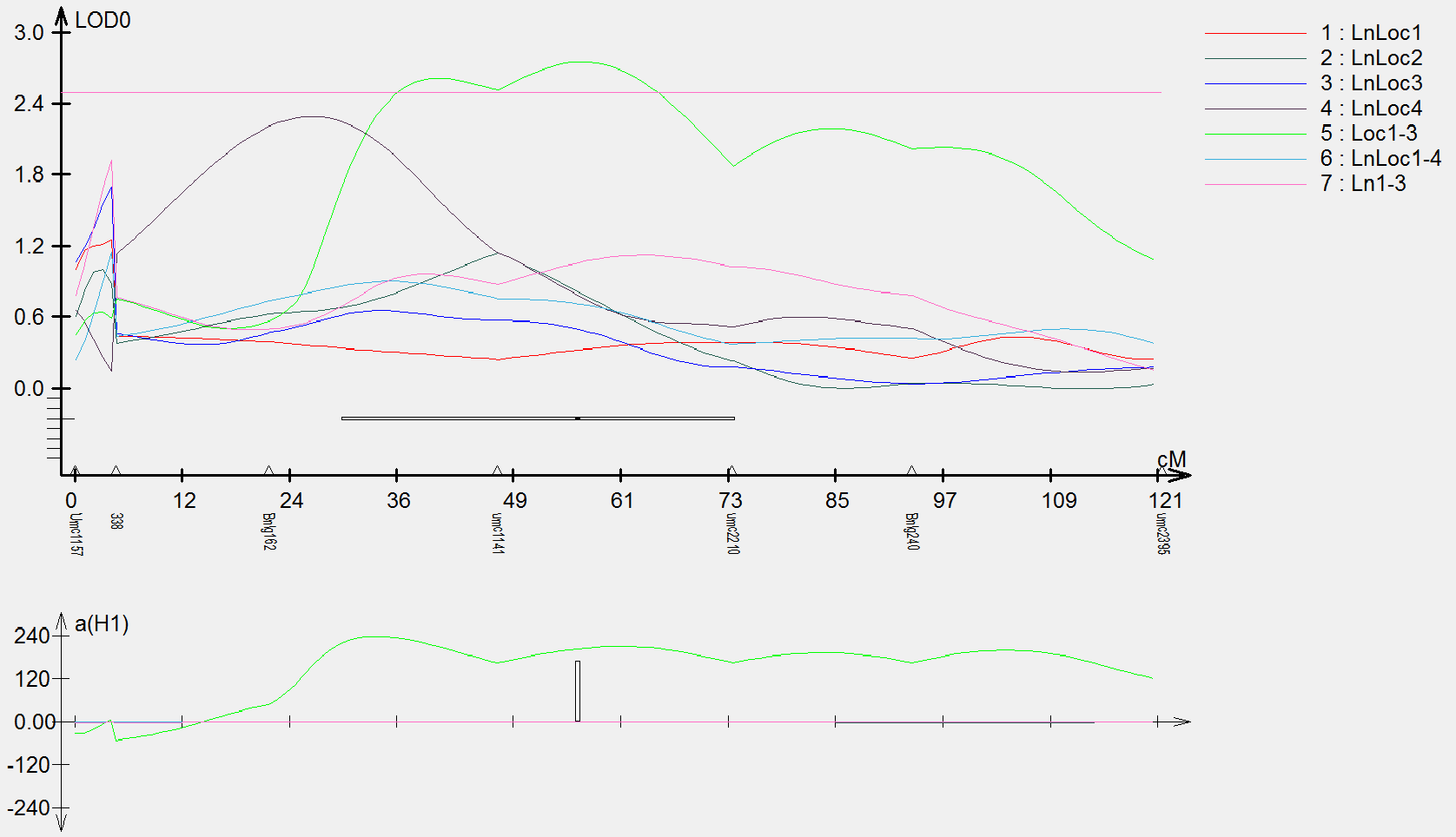

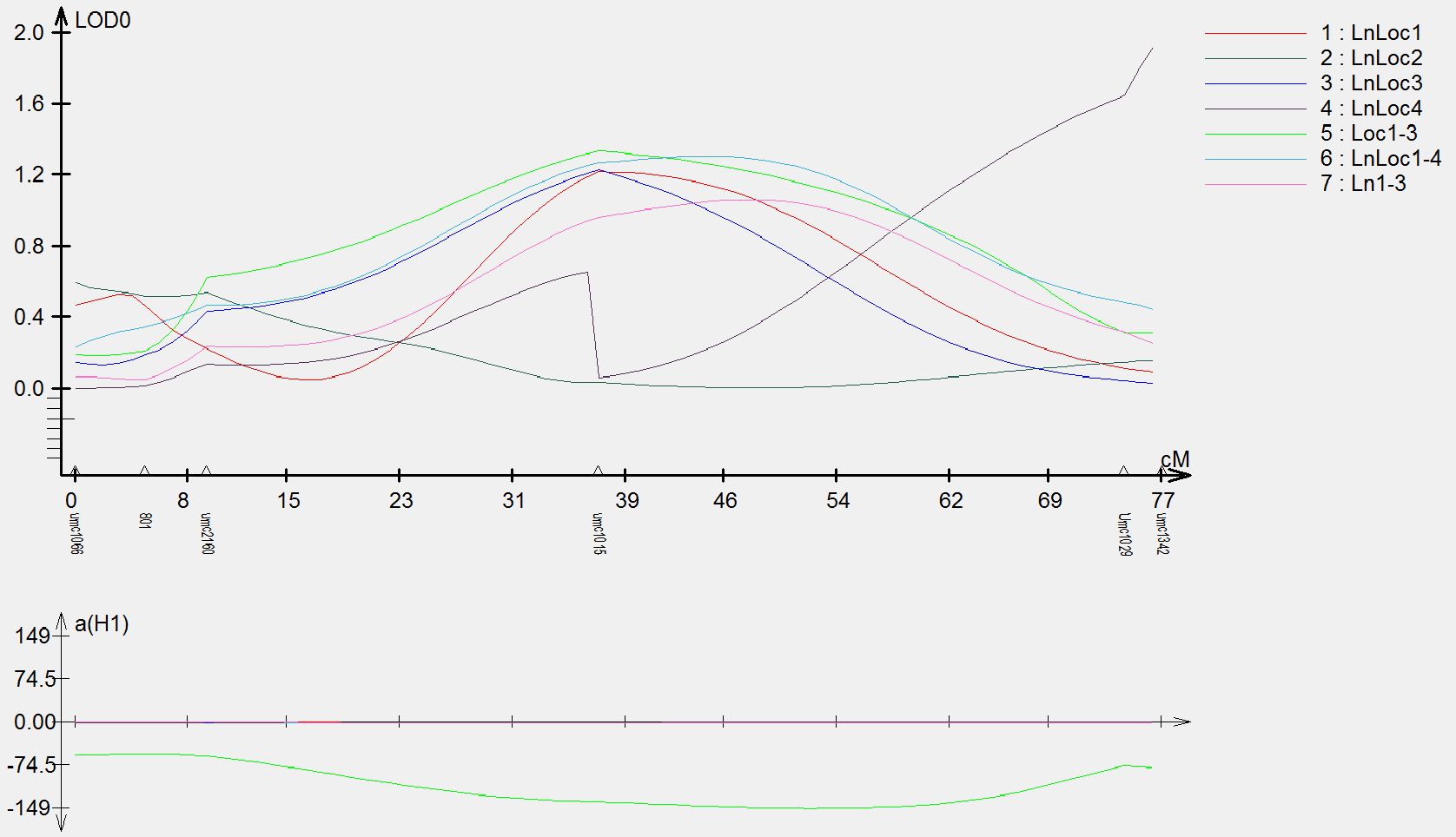

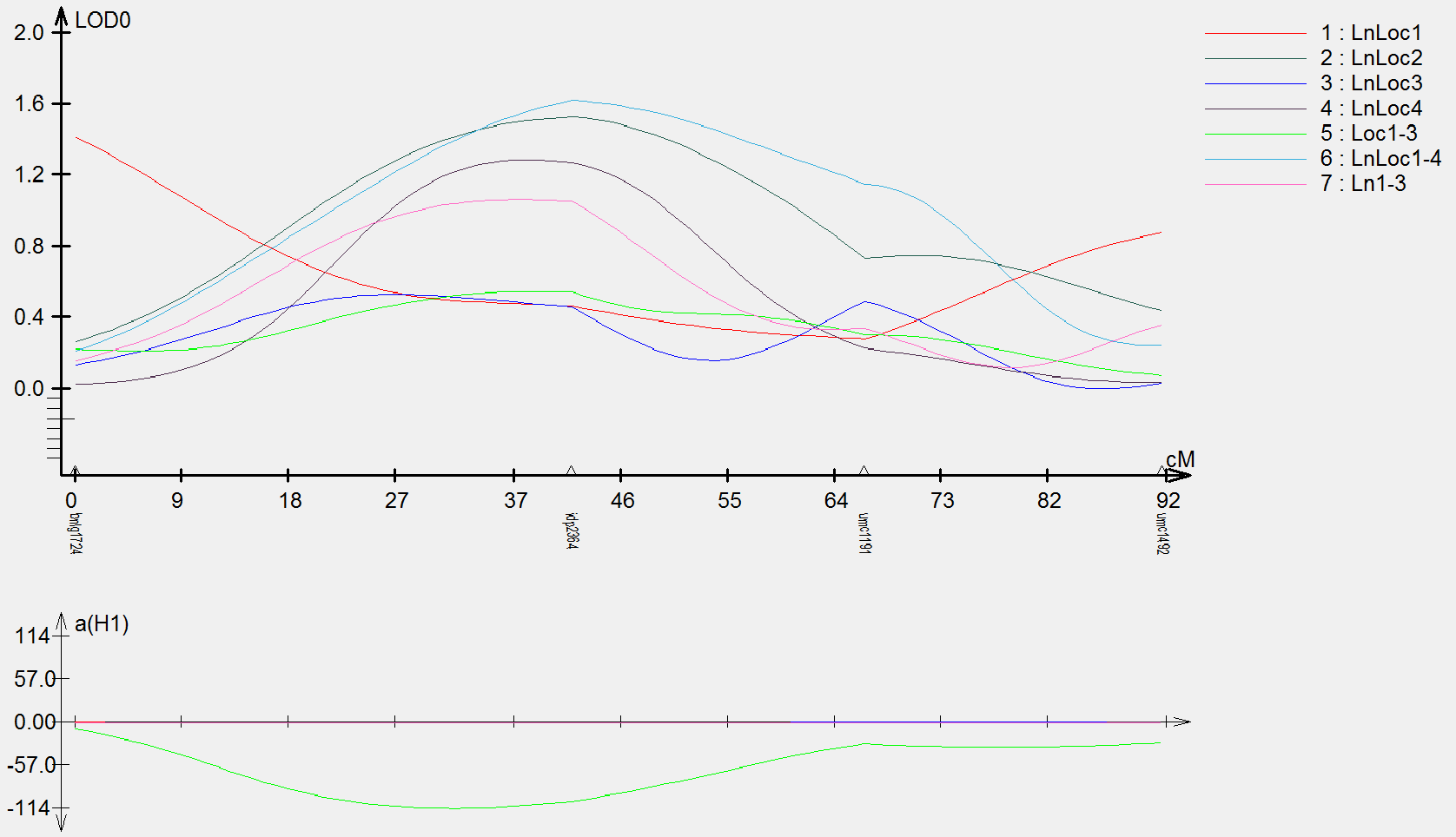

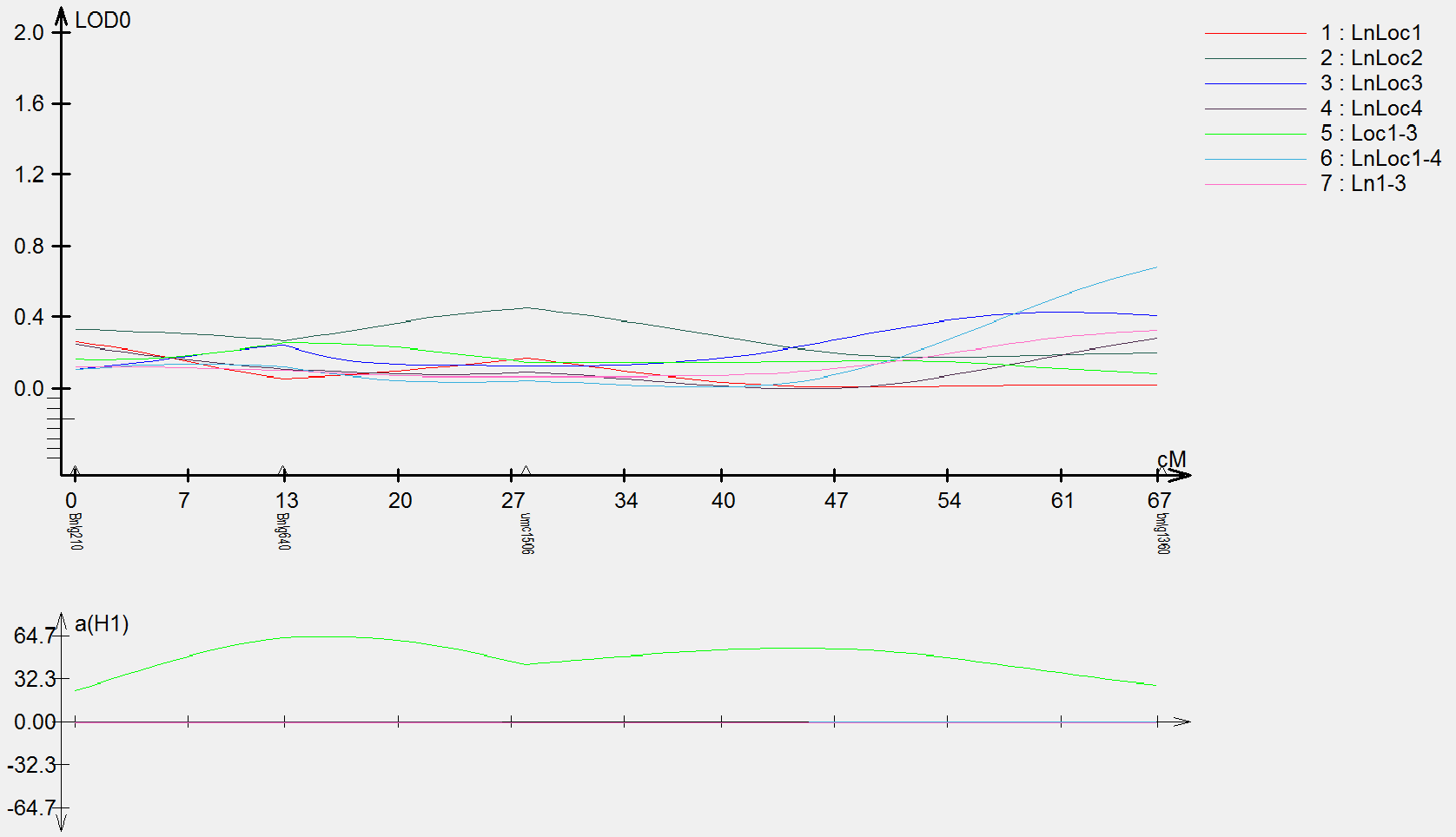
Chromosome 7 Chromosome 8

Chromosome 9 Chromosome 10


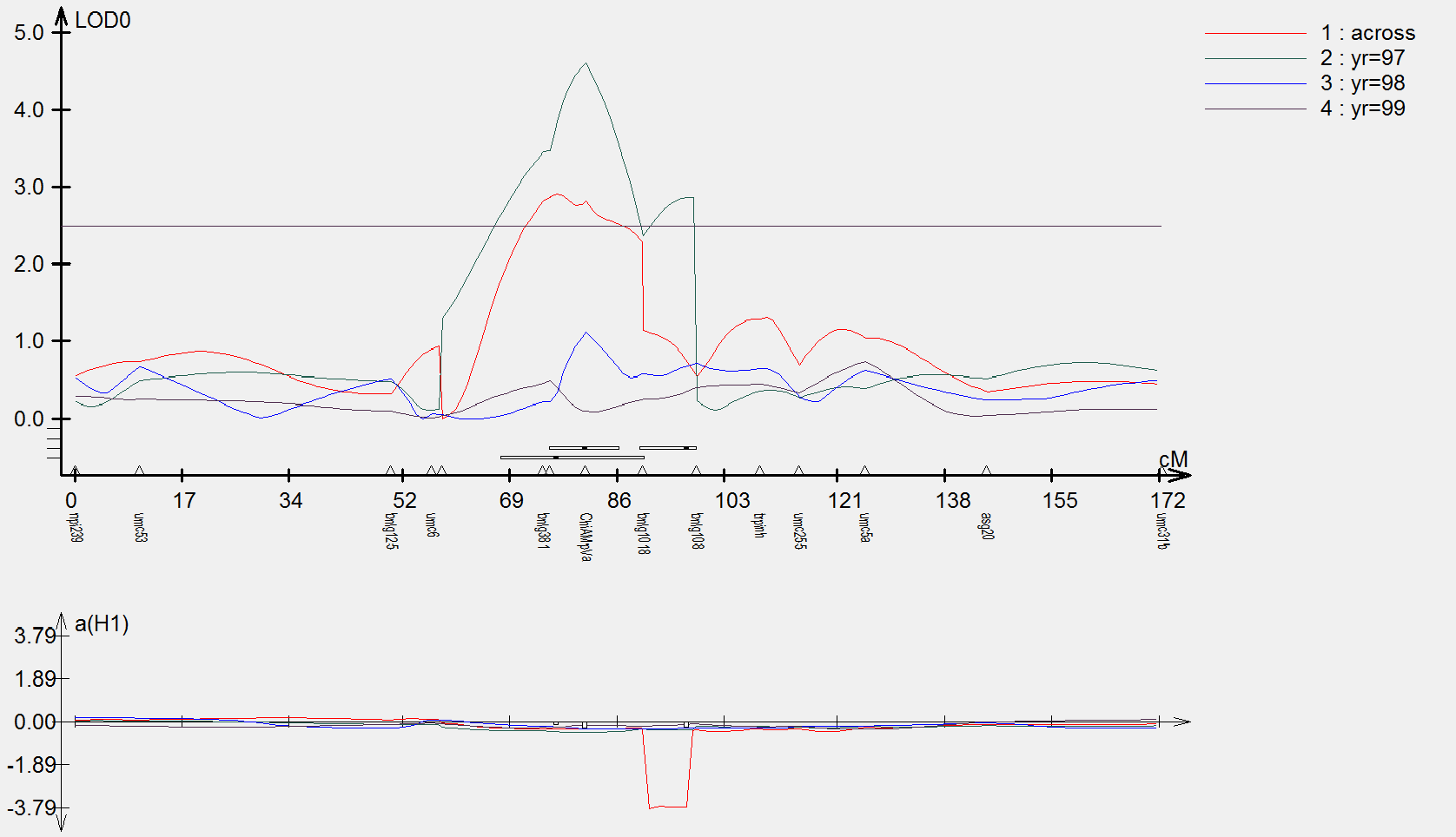

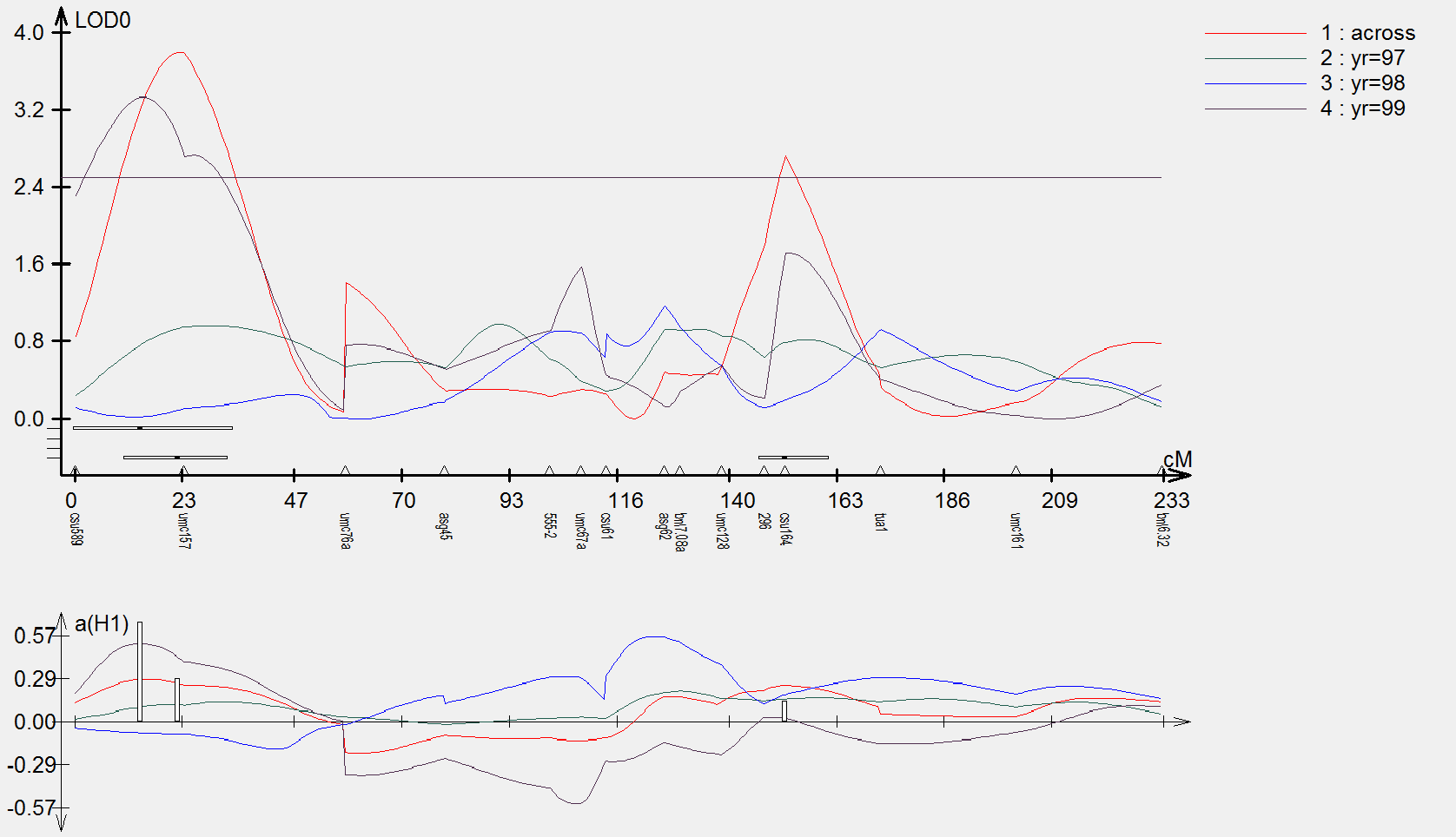
MpVa mapping population


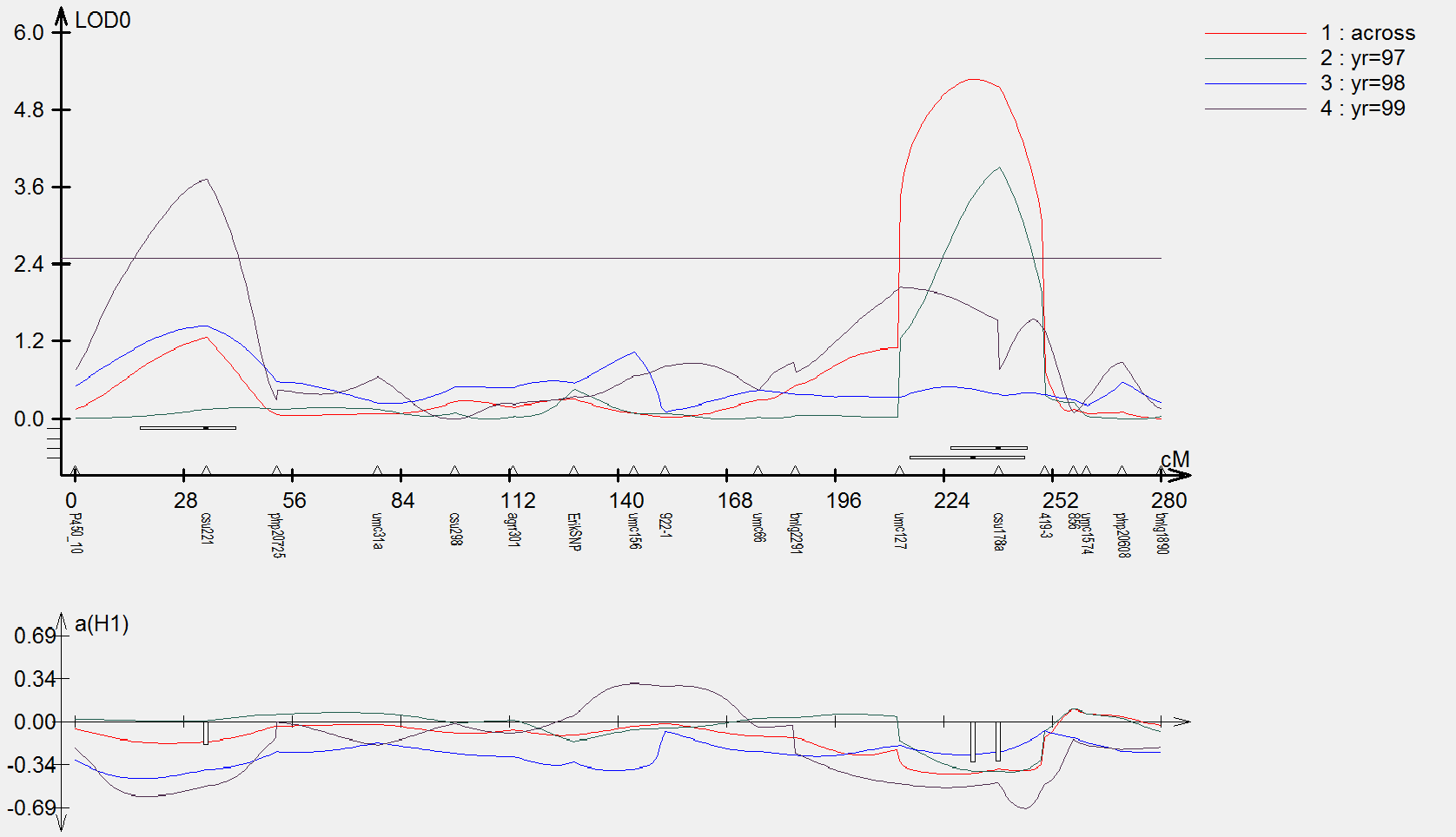

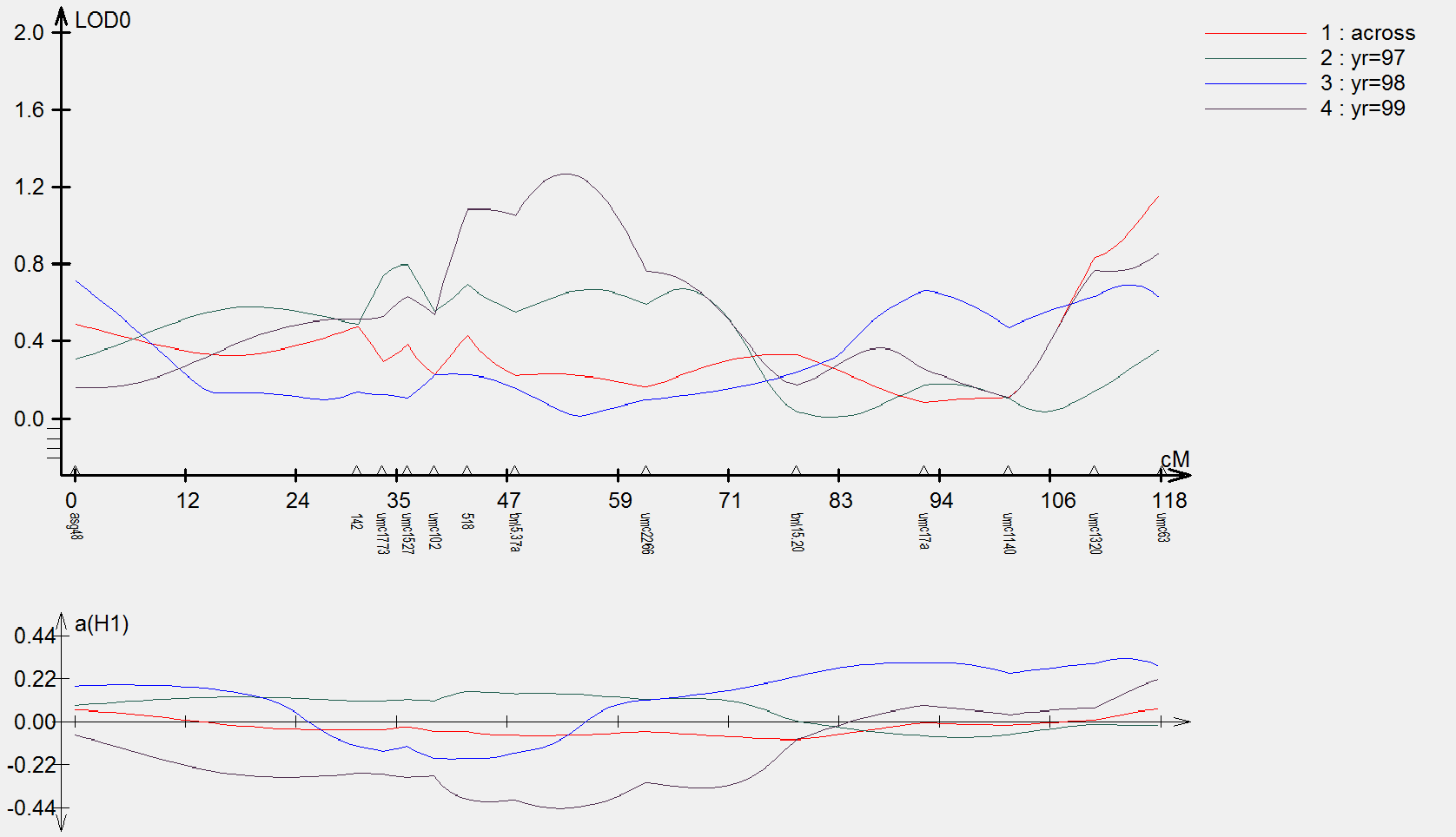
Chromosome 1 Chromosome 2

Chromosome 3 Chromosome 4


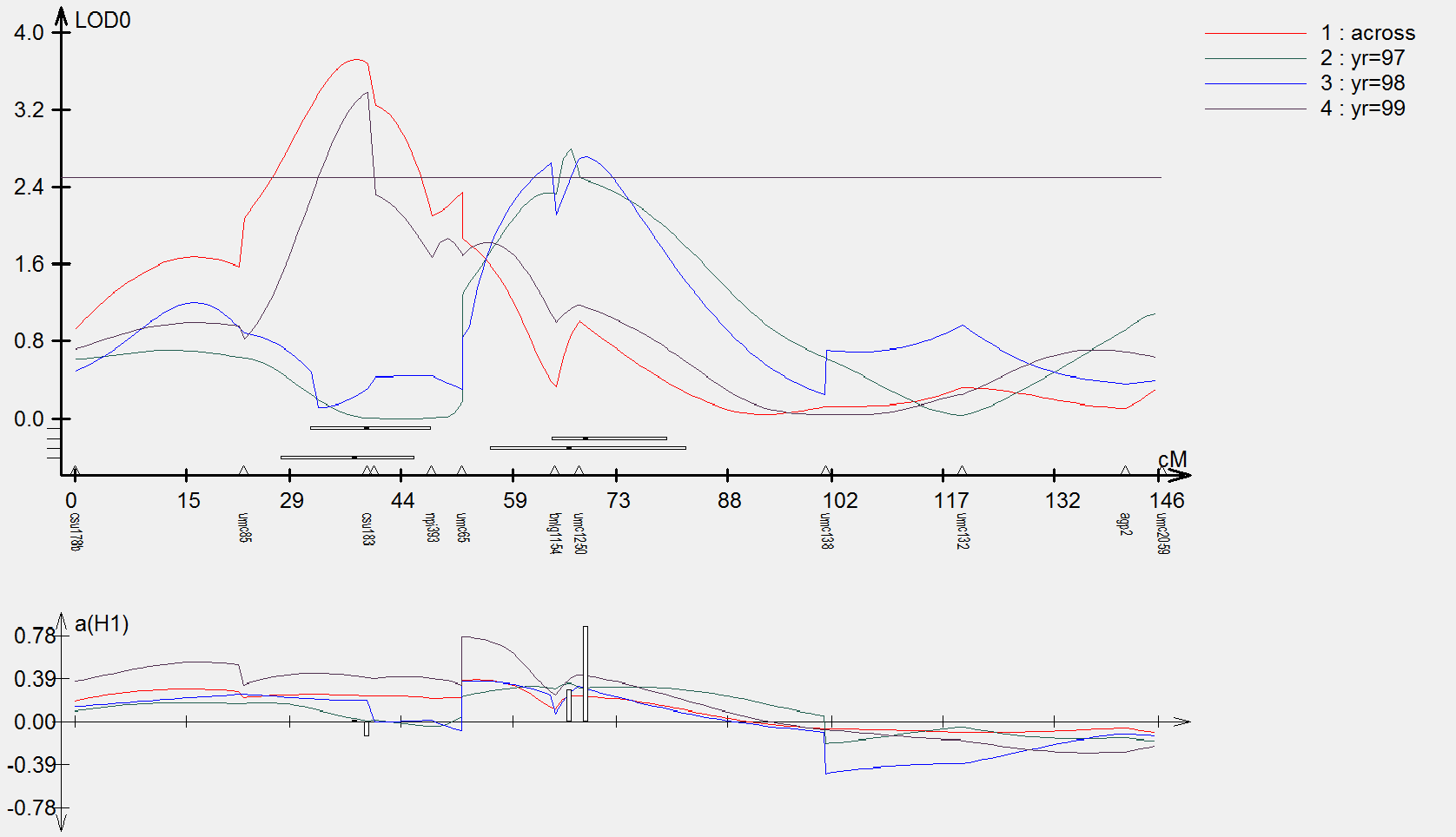

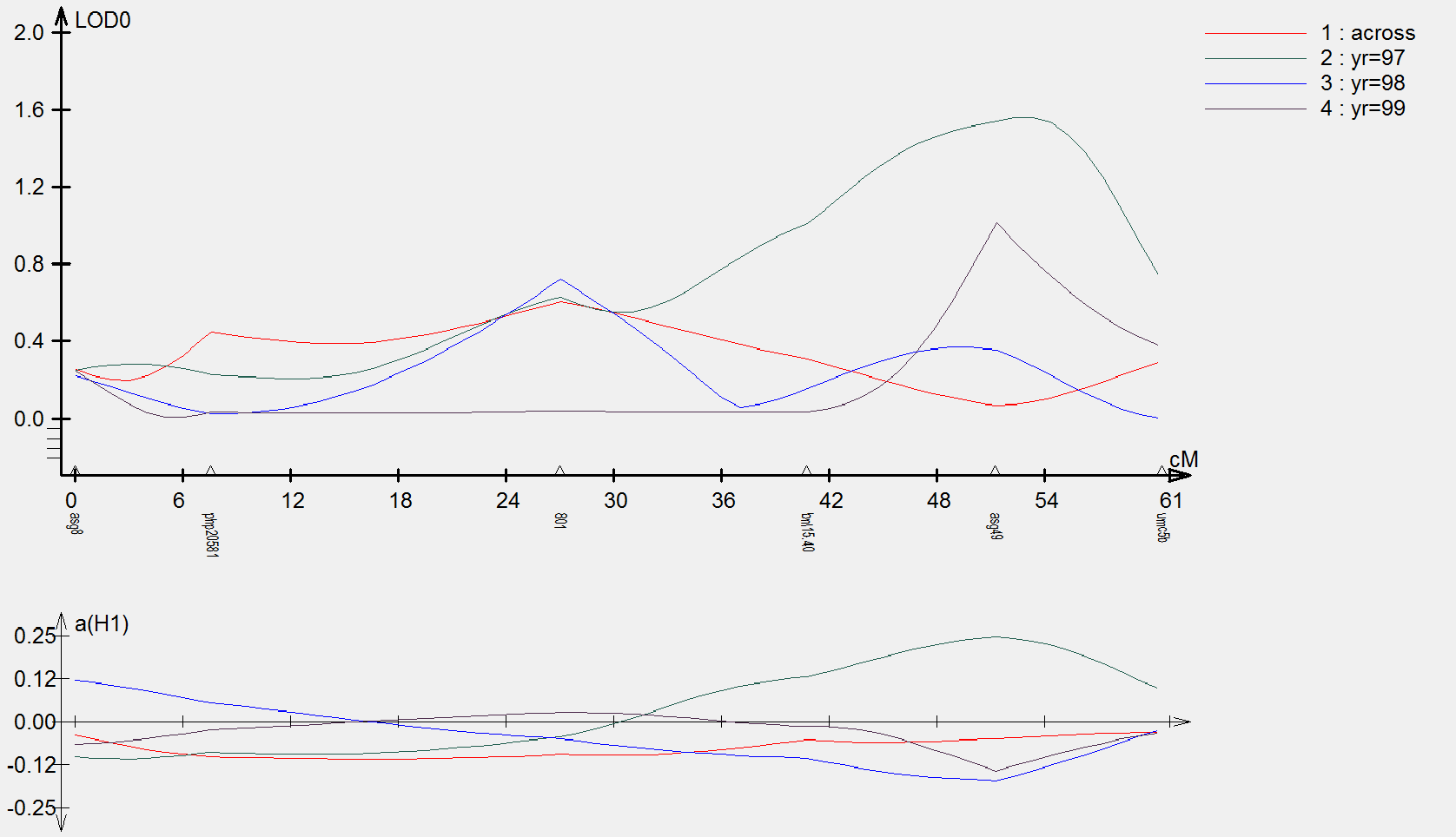

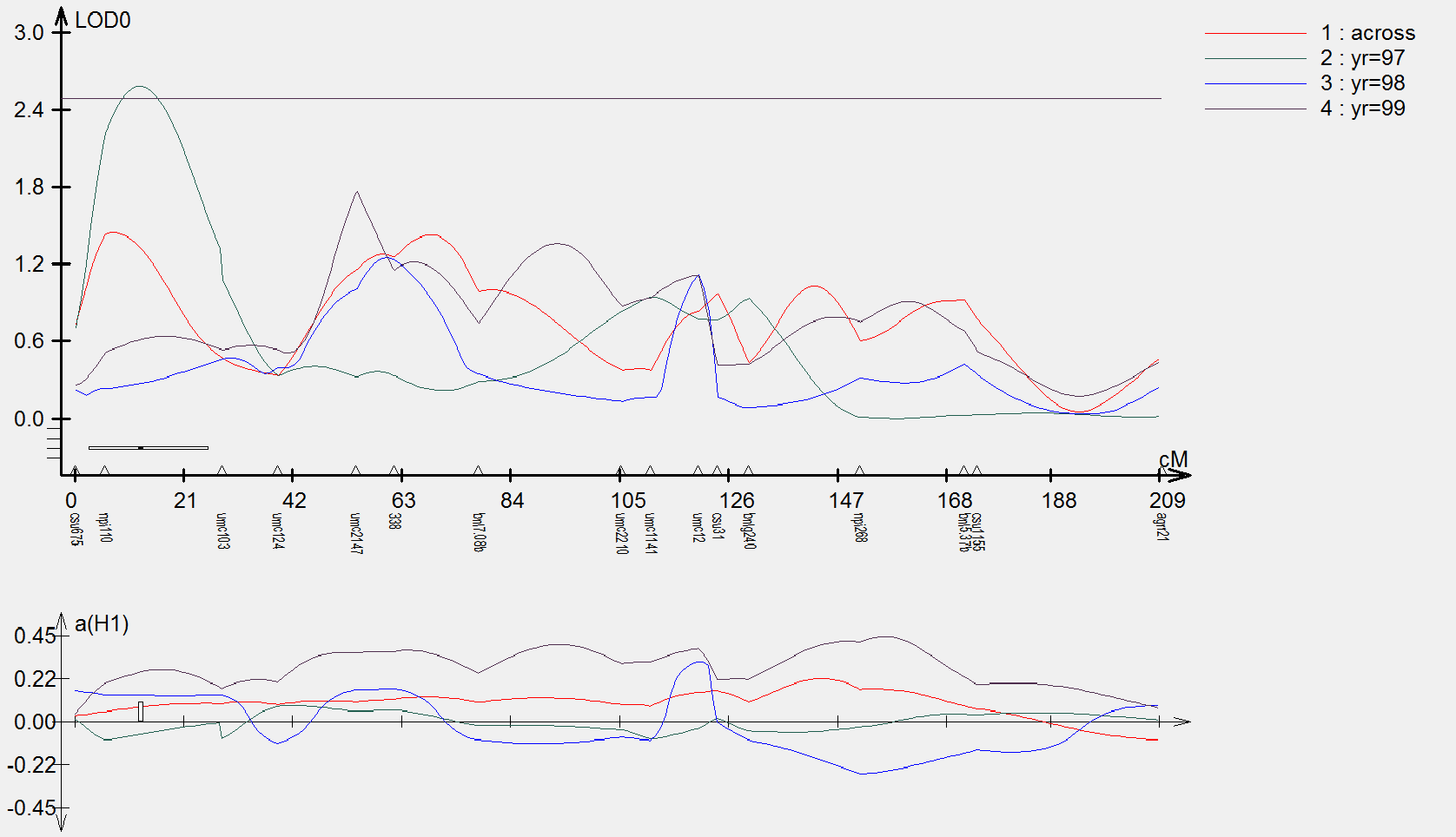

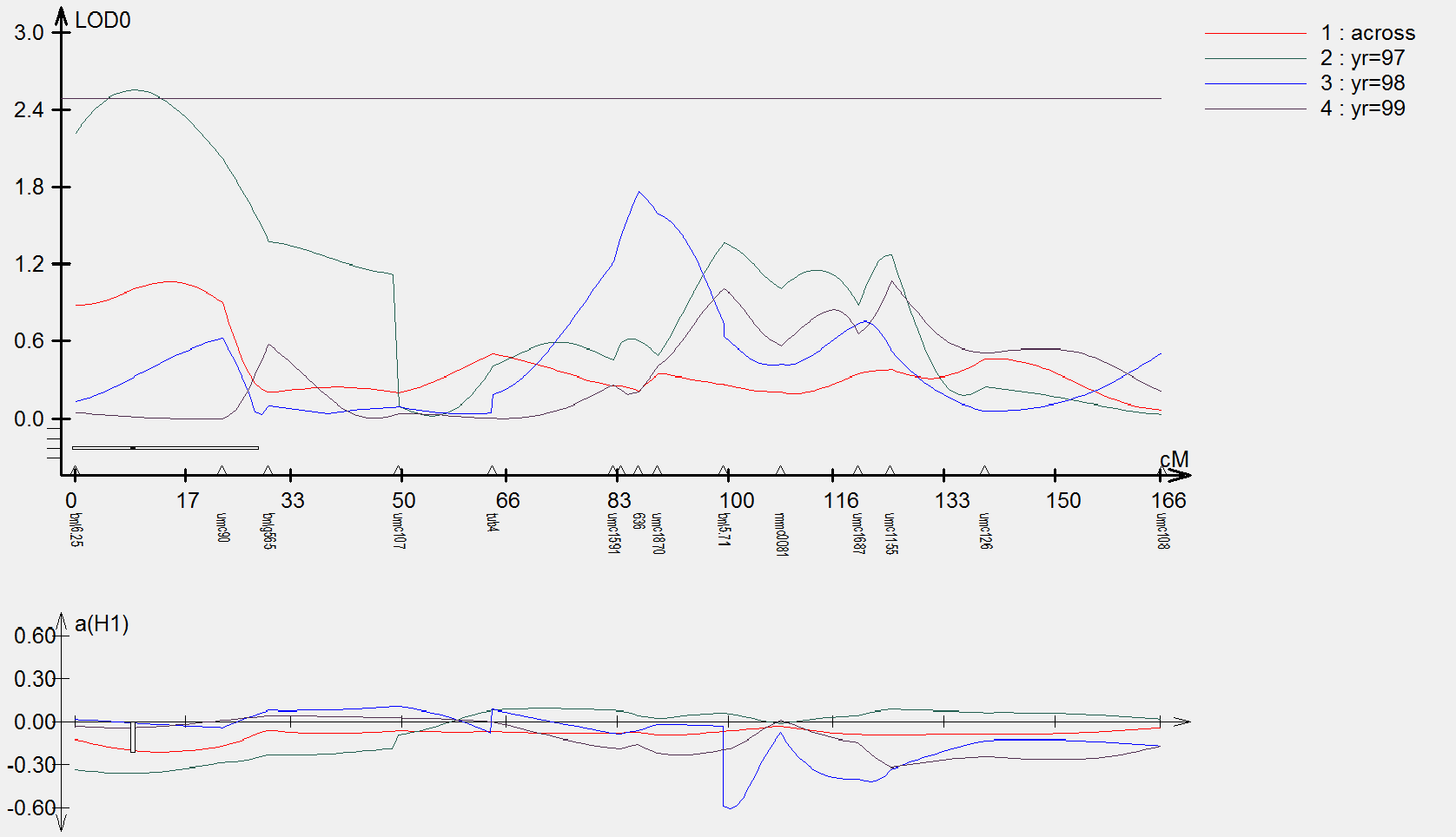
Chromosome 5 Chromosome 6


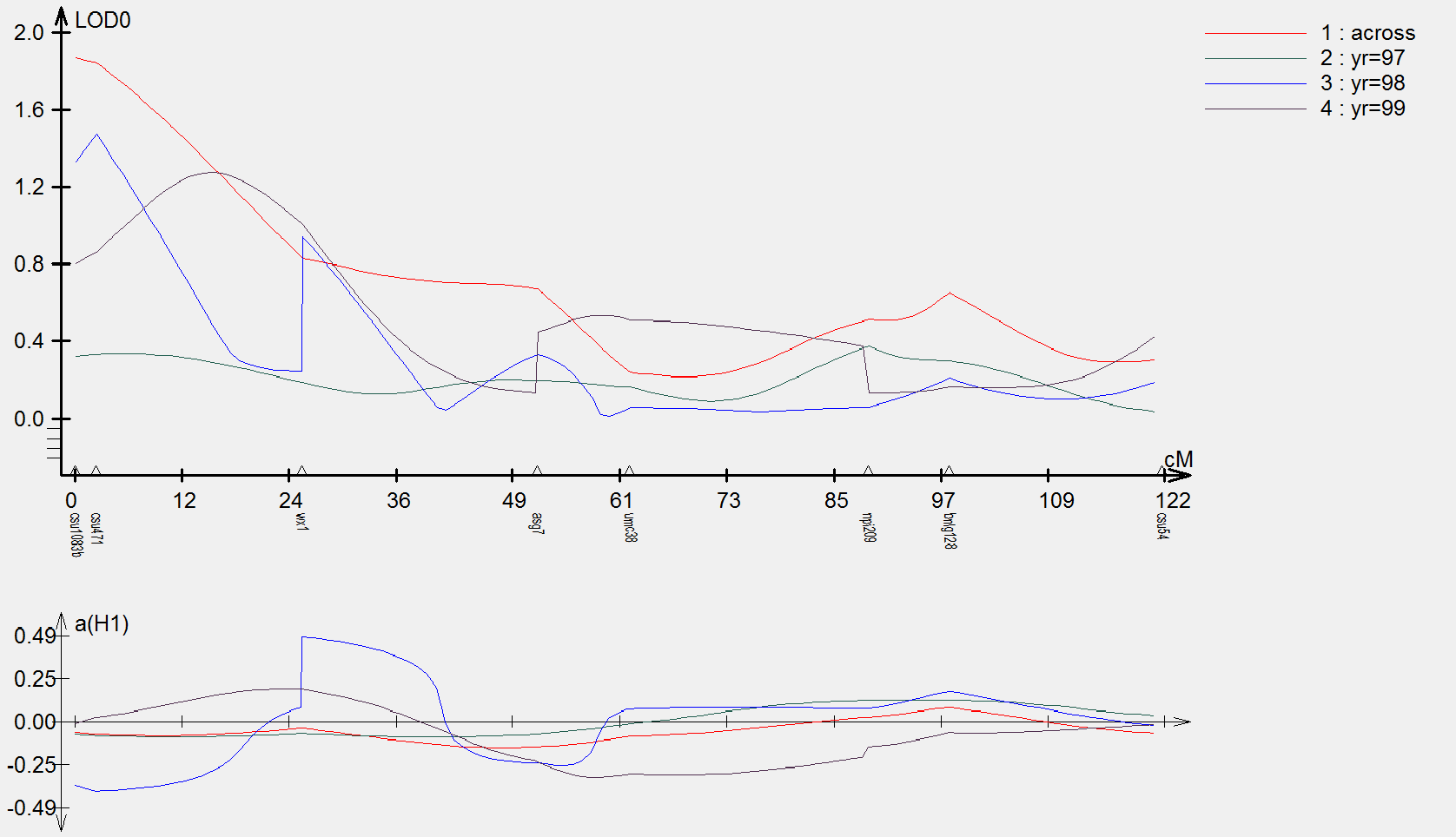

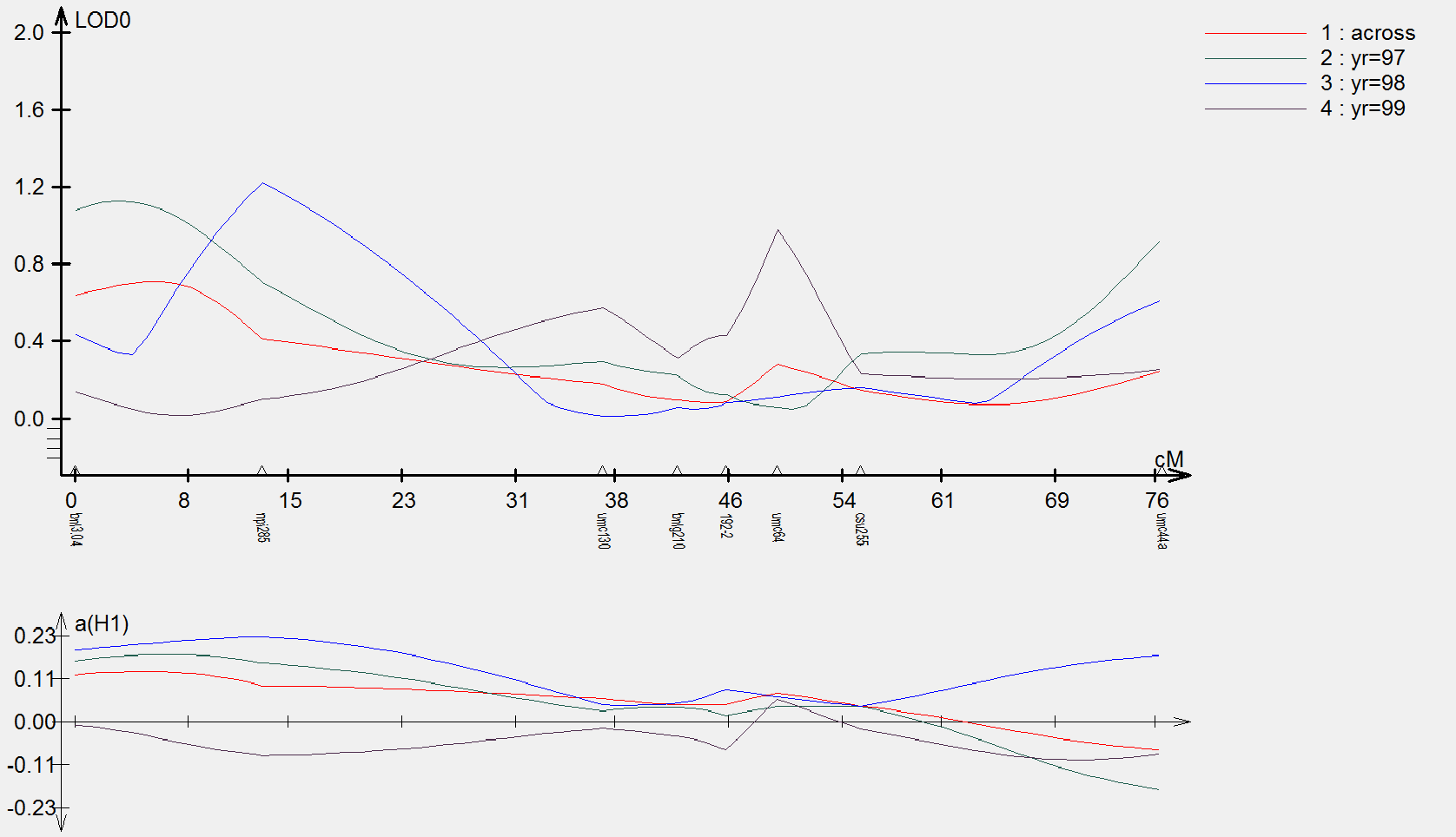
Chromosome 7 Chromosome 8

Chromosome 9 Chromosome 10

MpNC mapping population


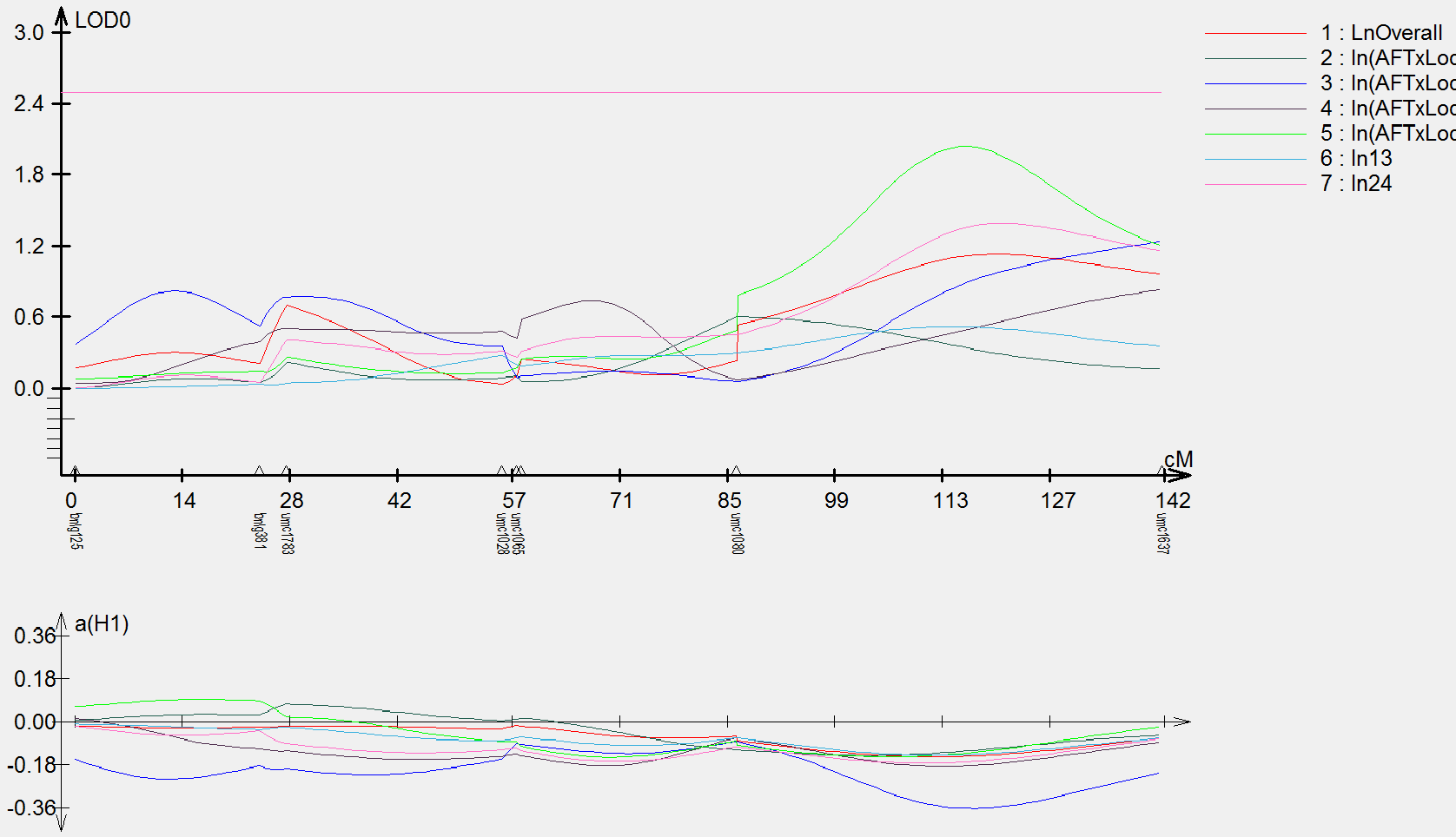

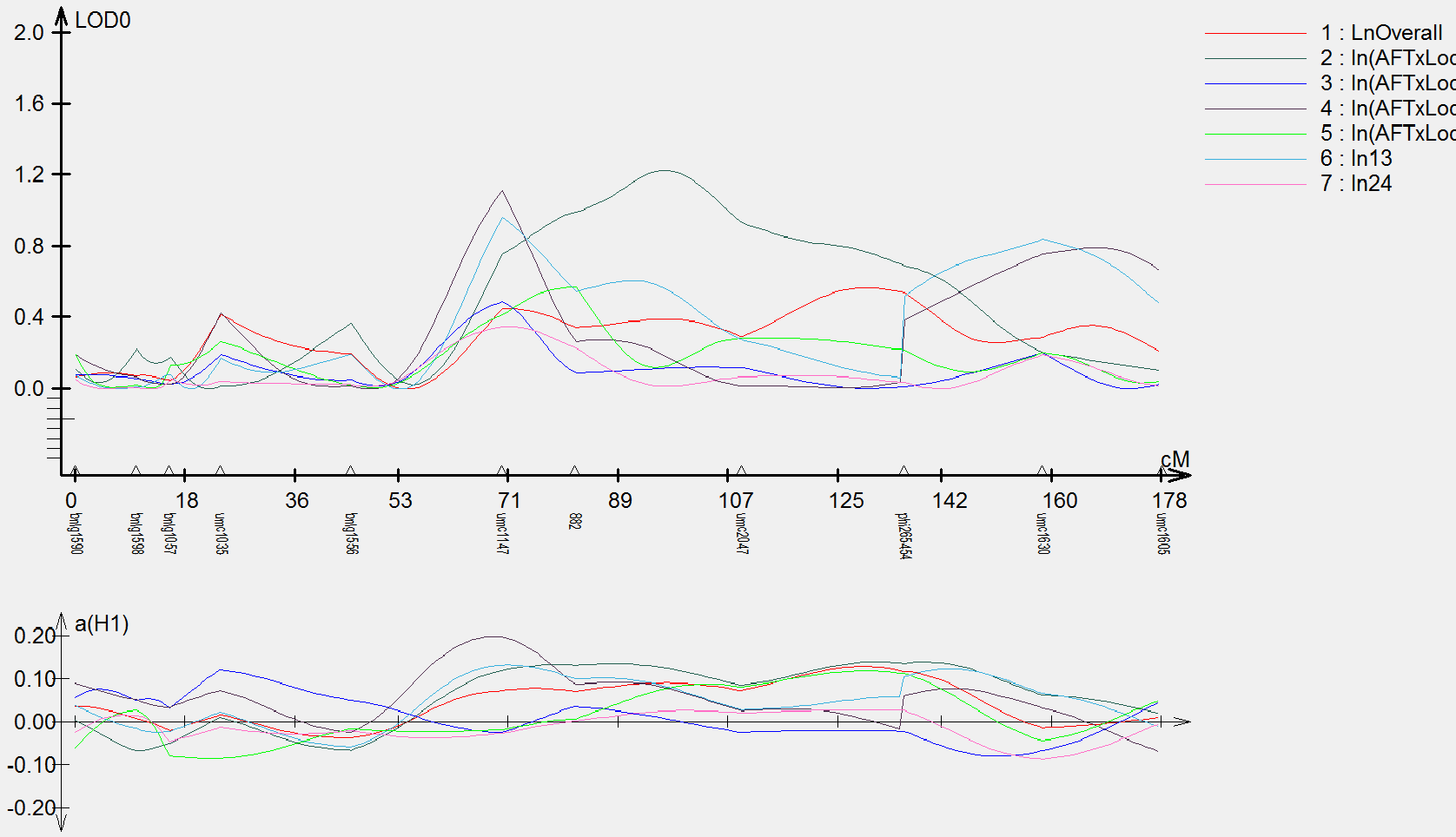
Chromosome 1 Chromosome 2


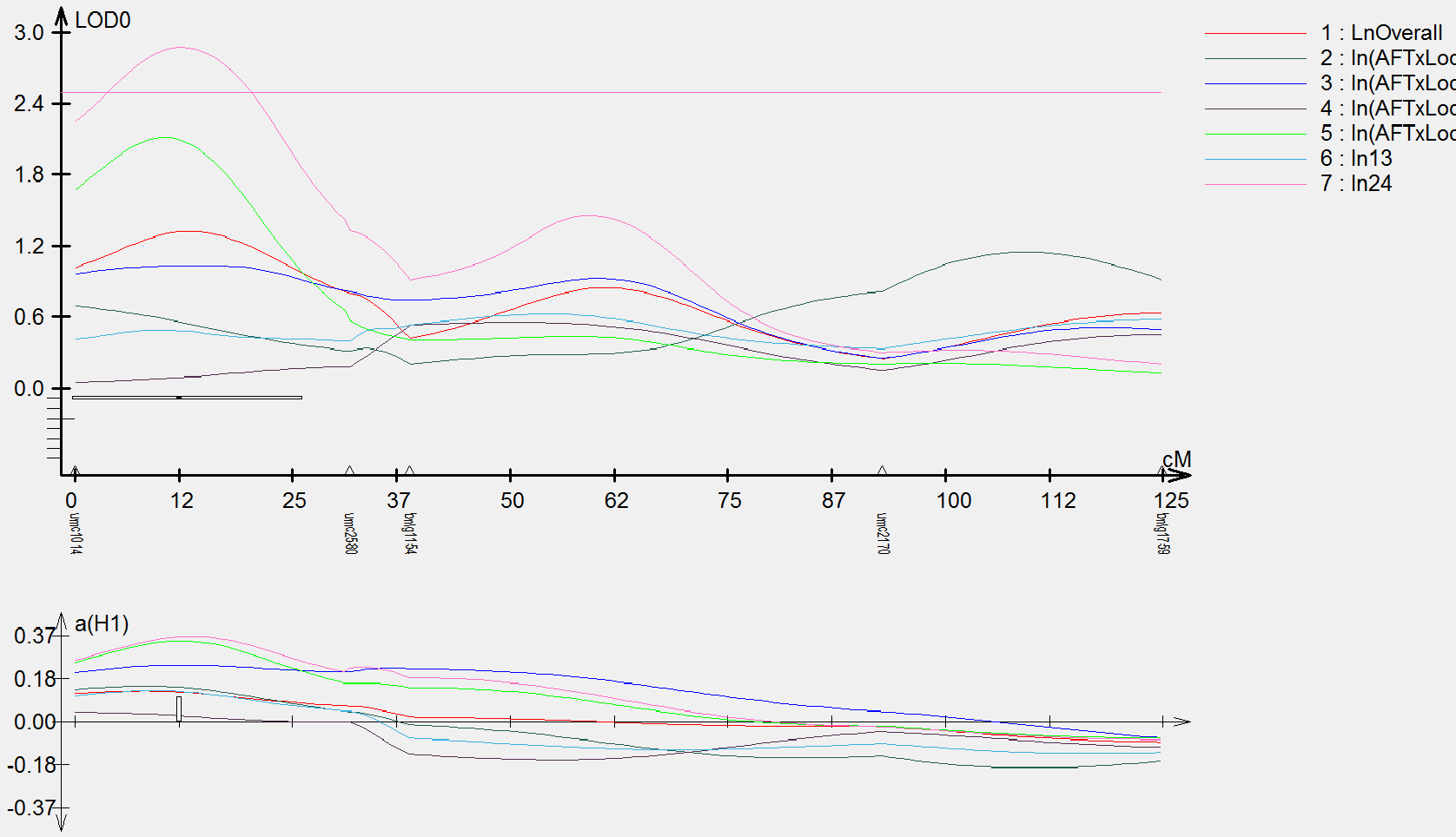

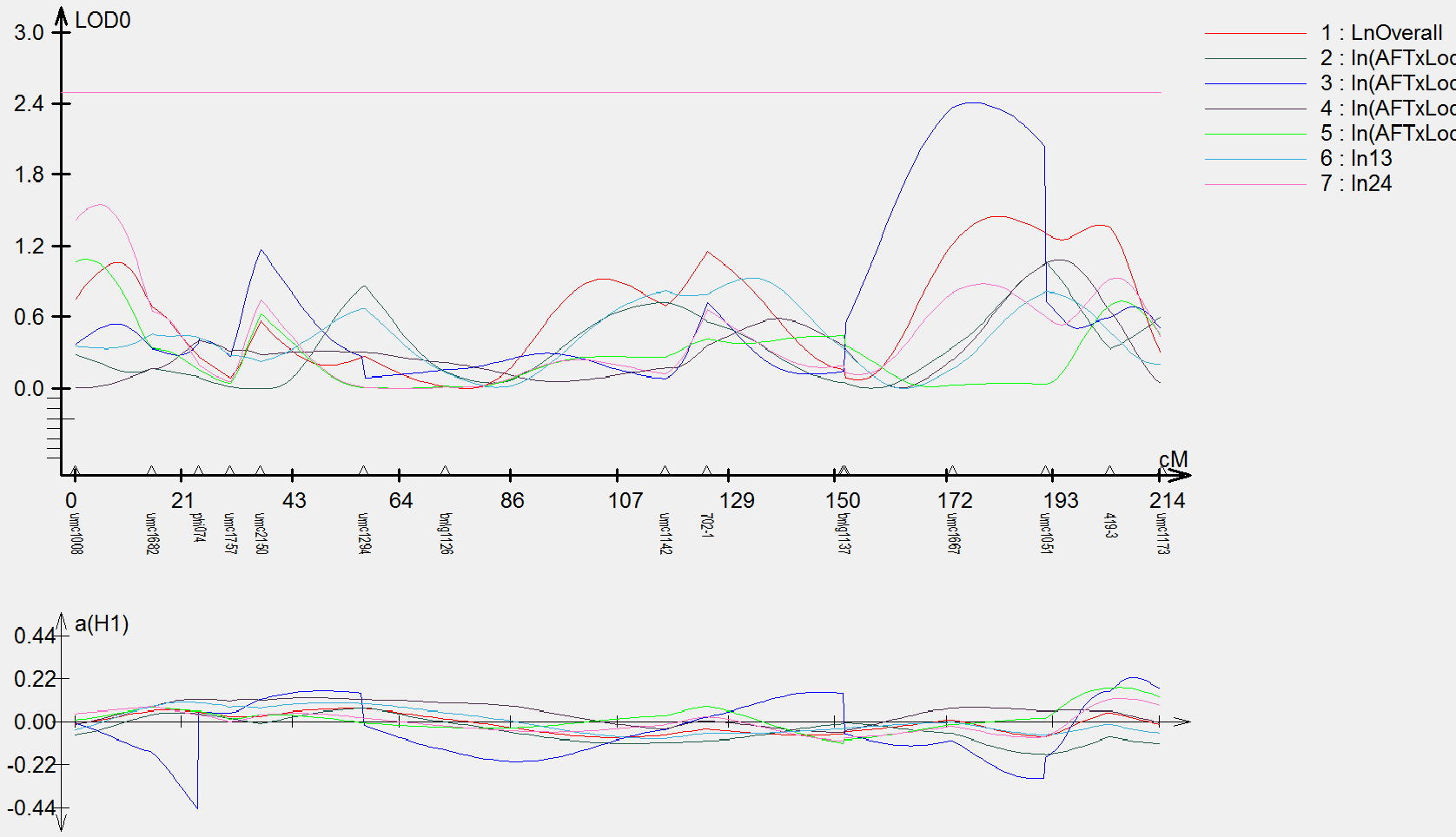

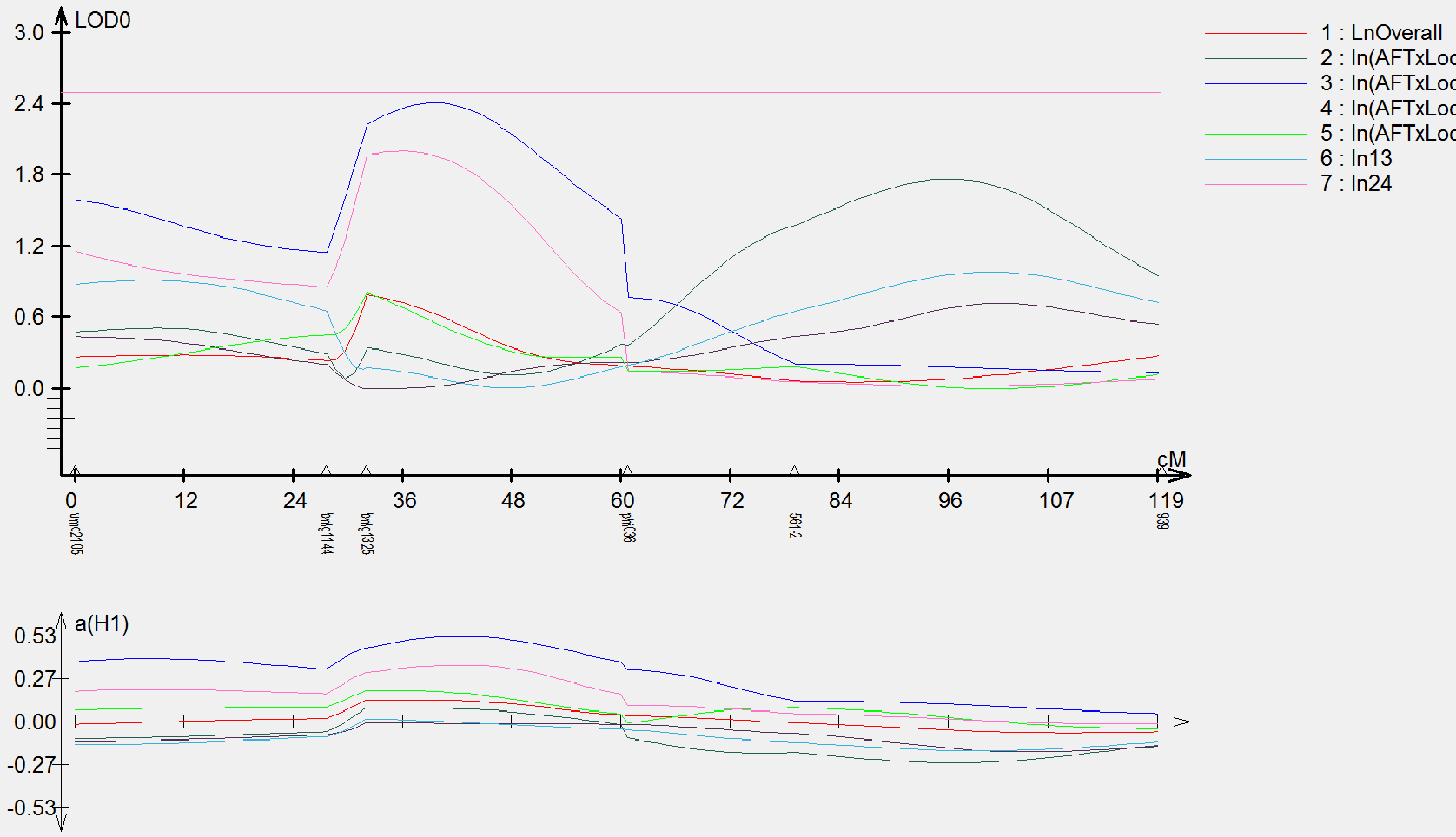
Chromosome 3 Chromosome 4


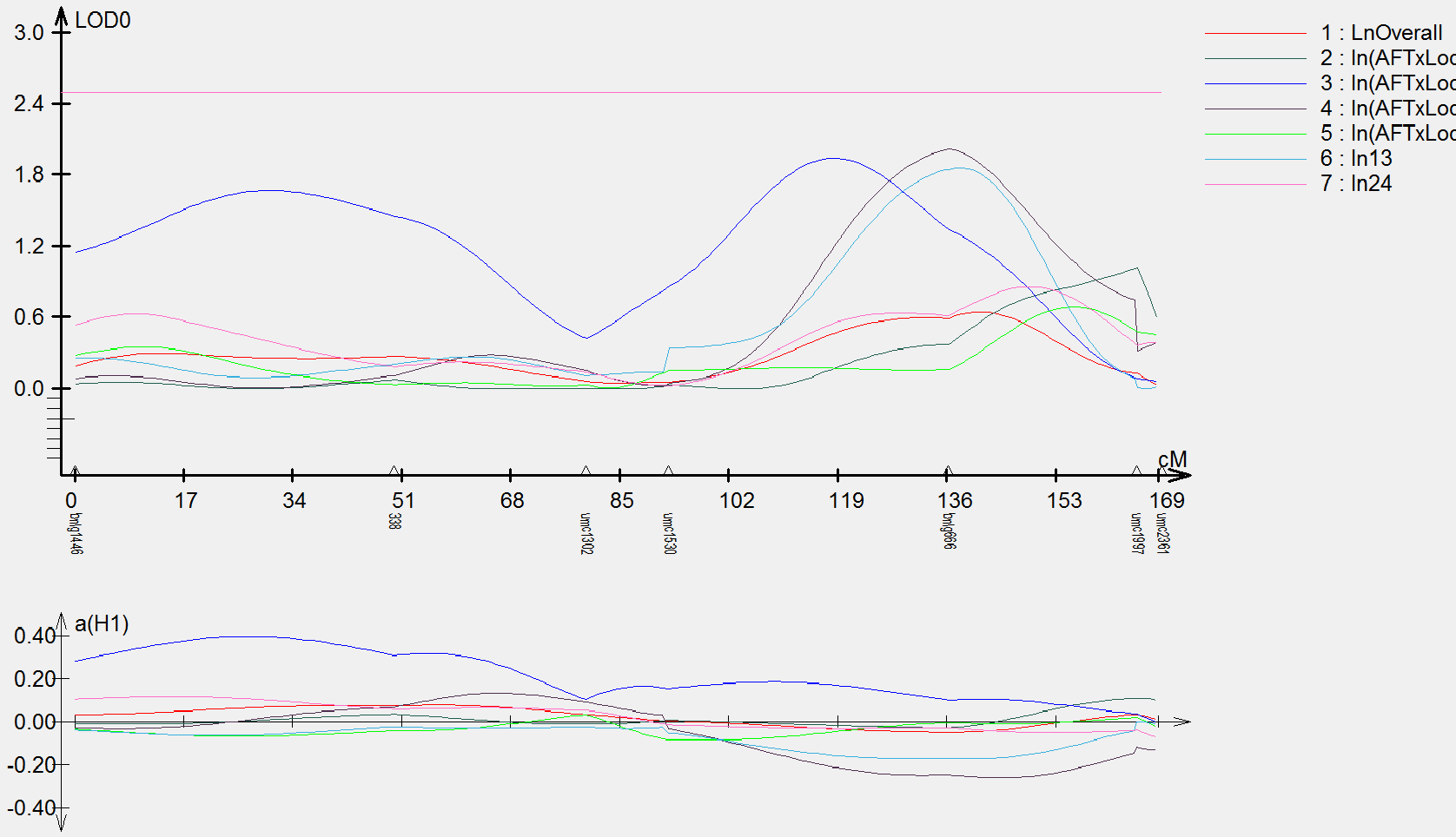

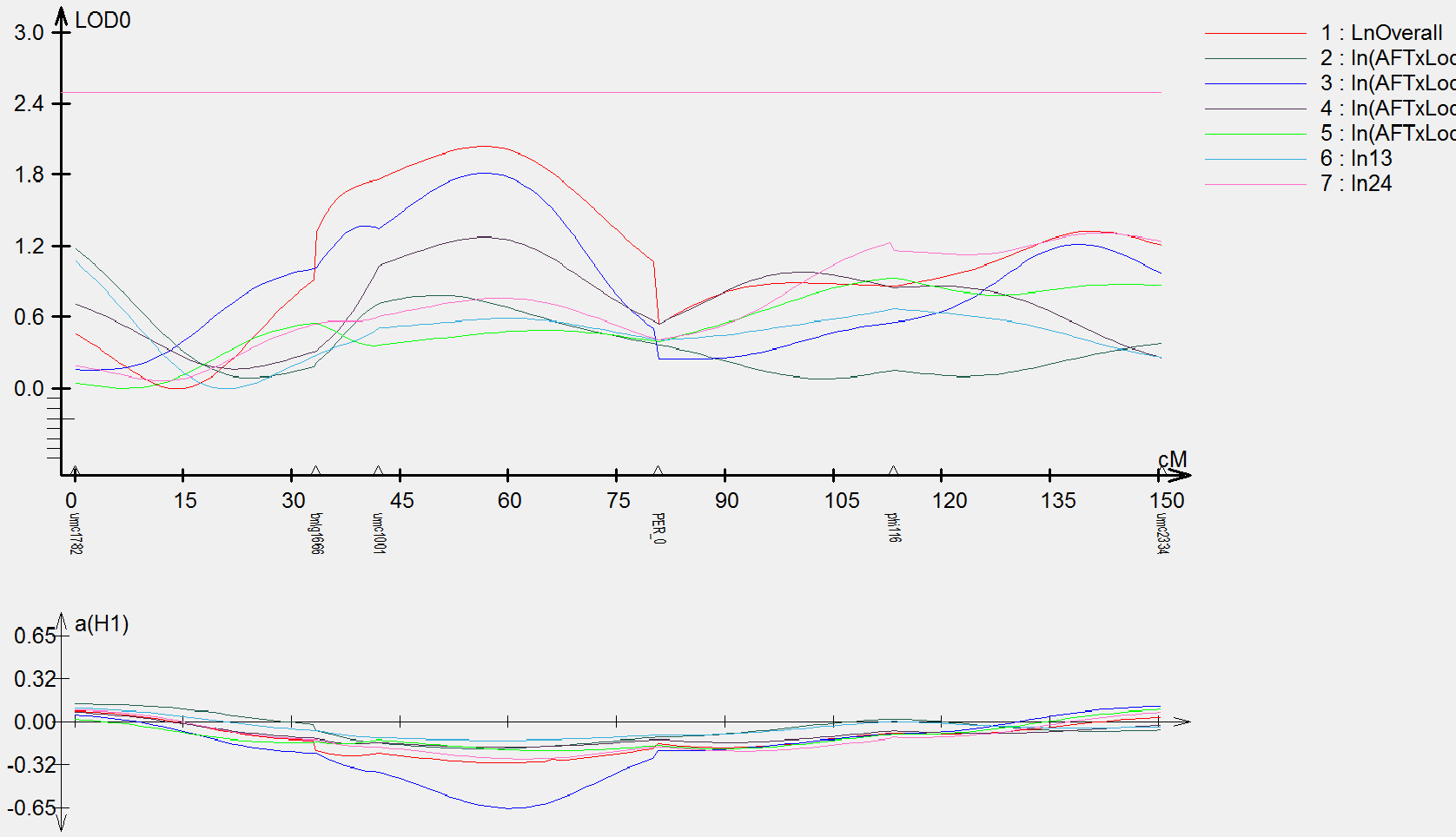

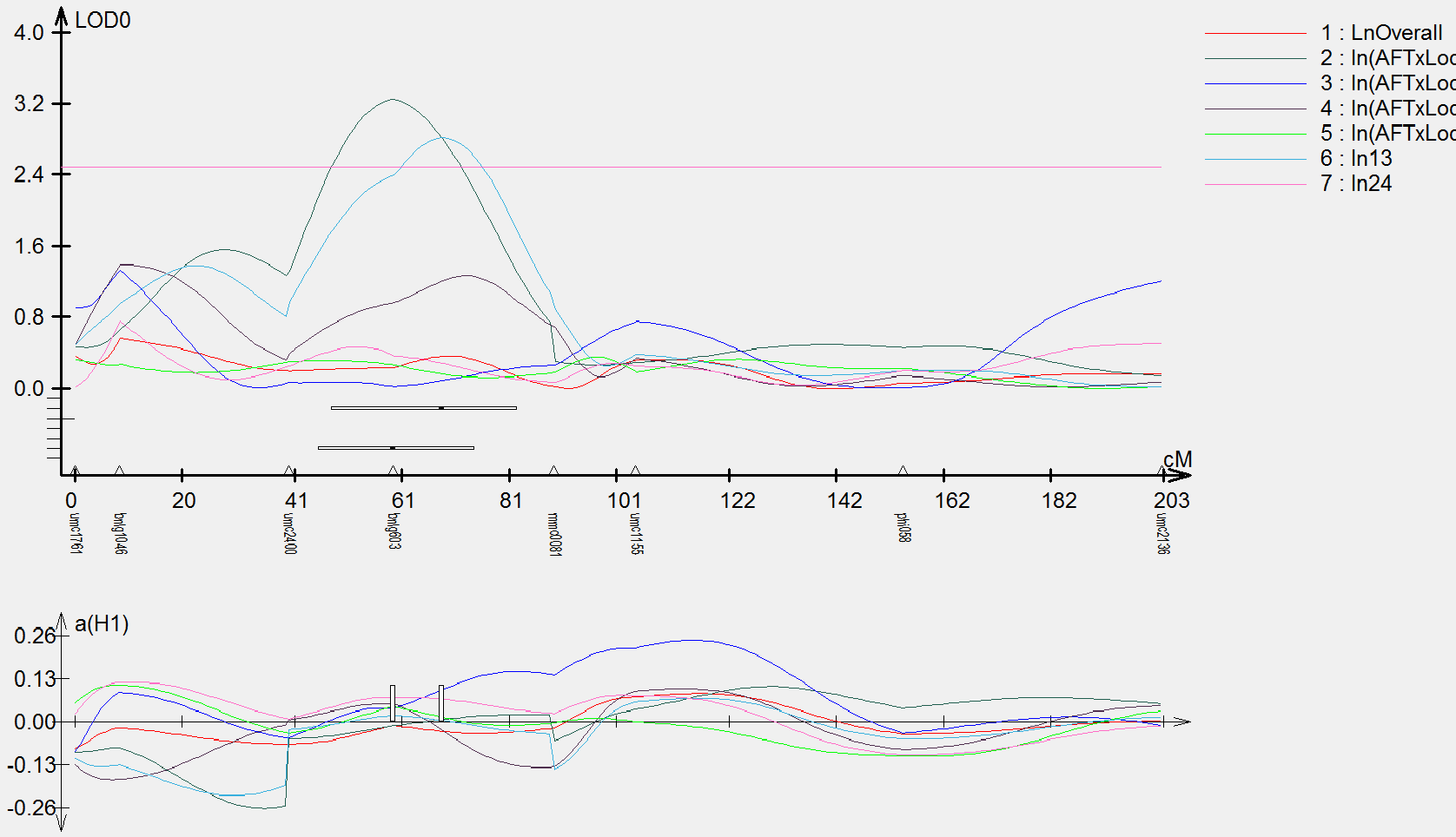
Chromosome 5 Chromosome 6

Chromosome 7 Chromosome 8


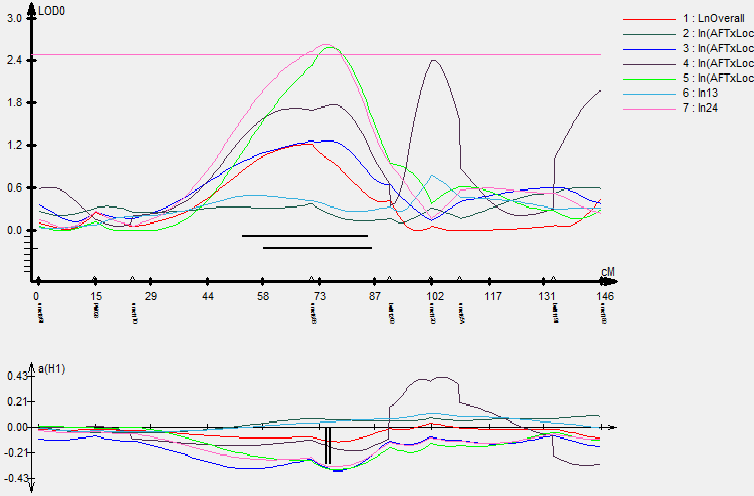

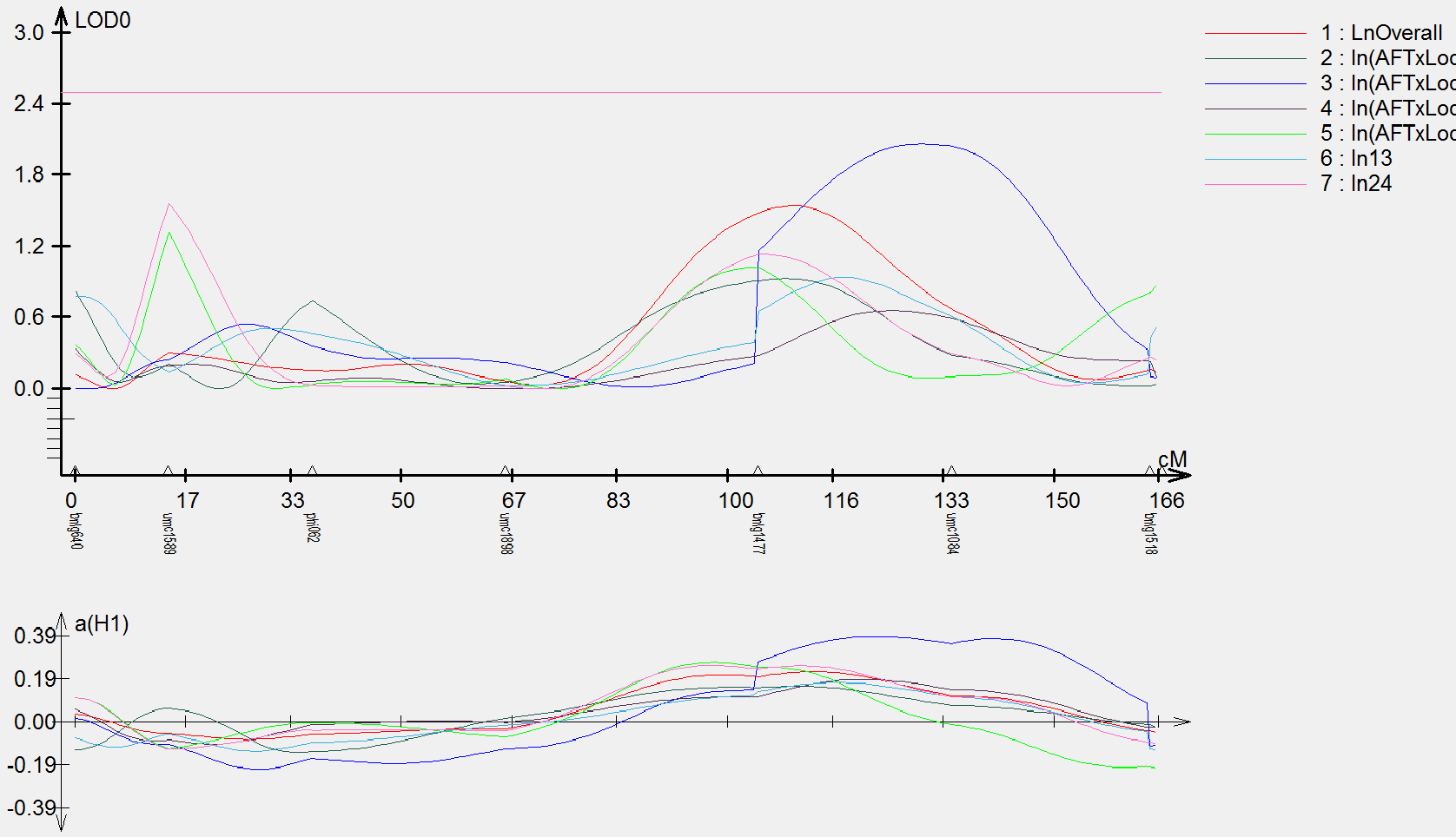
Chromosome 9 Chromosome 10
